# Supplementary material for: Rapid one-step 18F-radiolabeling of biomolecules in aqueous media by organophosphine fluoride acceptors
Source: Nat Commun. 2019 Mar 1;10:989. doi: 10.1038/s41467-019-08953-0 (PMC6397219; doi:10.1038/s41467-019-08953-0)
Supplement: Supplementary file 1 — Supplementary Information [file 41467_2019_8953_MOESM1_ESM.pdf]

Supplementary Information for

Rapid One-Step ^{18}F-Radiolabeling of Biomolecules in Aqueous Media by Organophosphine Fluoride Acceptors

Hong et al.

Table of Contents

Supplementary Figures

Supplementary Figure 1. Synthetic route of the organofluorophosphine fluoride acceptors. 5

Supplementary Figure 2. Synthetic route of DBPOF-c(RGDyk). 6

Supplementary Figure 3. Synthetic route of DBPOF-HSA....7

Supplementary Figure 4. Automated radiosynthesis module setup for ^{18}F-DBPOF-c(RGDyk). 8

Supplementary Figure 5. *In vitro* stabilities of ^{18}F labeled organofluorophosphine.. 10

Supplementary Figure 6. Metabolic stability of ^{18}F labeled organofluorophosphine in normal ICR mice....11

Supplementary Figure 7. MicroPET/CT analysis of *in vivo* stabilities of [^{18}F]**4** 12

Supplementary Figure 8. UV standard curve of DBPOF-c(RGDyk) 13

Supplementary Figure 9. Radio-HPLC analysis of RCP of ^{18}F-DBPOF-c(RGDyk)..14

Supplementary Figure 10. Radio-HPLC analysis of RCP of ^{18}F-DBPOF-HSA 15

Supplementary Figure 11. Radio-HPLC analysis of *in vitro* stabilities of ^{18}F-DBPOF-c(RGDyk) 16

Supplementary Figure 12. Radio-HPLC analysis of *in vitro* stabilities of ^{18}F-DBPOF-HSA....17

Supplementary Figure 13. MicroPET/CT imaging of the mice bearing U87MG tumors 18

Supplementary Figure 14. Time-activity curves of ^{18}F-DBPOF-c(RGDyk) in the control group....19

Supplementary Figure 15. Cell cytotoxic analysis of DBPOF-c(RGDyk) in U87MG cell. 20

Supplementary Figure 16. Reaction pathways and free-energy profiles....22

Supplementary Figure 17. Charge distribution of organofluorophosphine fluoride acceptors **1-5**. 23

Supplementary Figure 18. Delocalized molecular orbital of organofluorophosphine fluoride acceptors **1-5**. 24

Supplementary Figure 19-30. Radio-HPLC profiles 24

Supplementary Figure 31-63. Characteration spectra of synthesied compounds....24

Supplementary Tables

Supplementary Table 1. RCYs of selected flourophosphine fluoride acceptors. 70

Supplementary Table 2. Summary of radiosynthesis of ^{18}F-DBPOF-c(RGDyk) 71

Supplementary Methods

General information 72
Synthetic methods of organofluorophosphine fluoride acceptors 72-77
Synthesis of DBPOF-biomolecule precursors 77-77
Radiosynthesis of ^{18}F-labeled organofluorophosphine 78
^{18}F-labeling condition optimization for organofluorophosphine 78
In vitro stabilities of ^{18}F-labeled organofluorophosphines 79
Metabolic stabilities of ^{18}F-labeled organofluorophosphines in normal mice 80
MicroPET/CT imaging with [^{18}F]**4** 80
Automated radiosynthesis of ^{18}F-DBPOF-c(RGDyk) 80
Quality control of ^{18}F-DBPOF-c(RGDyk) 81-83
The standard UV curve of DBPOF-c(RGDyk) 83
Calculation of molar activities of ^{18}F-DBPOF-c(RGDyk) 83
In vitro stabilities of ^{18}F-labeled DBPOF-biomolecules 84
Glioblastoma tumor mouse model 85
MicroPET/CT imaging with ^{18}F-labeled DBPOF-biomolecules 85
Log *D* of ^{18}F-DBPOF-c(RGDyk) 86
Cell cytotoxic analysis of DBPOF-c(RGDyk) 86
Theoretical calculation 87

Supplementary References 87

Supplementary Figures

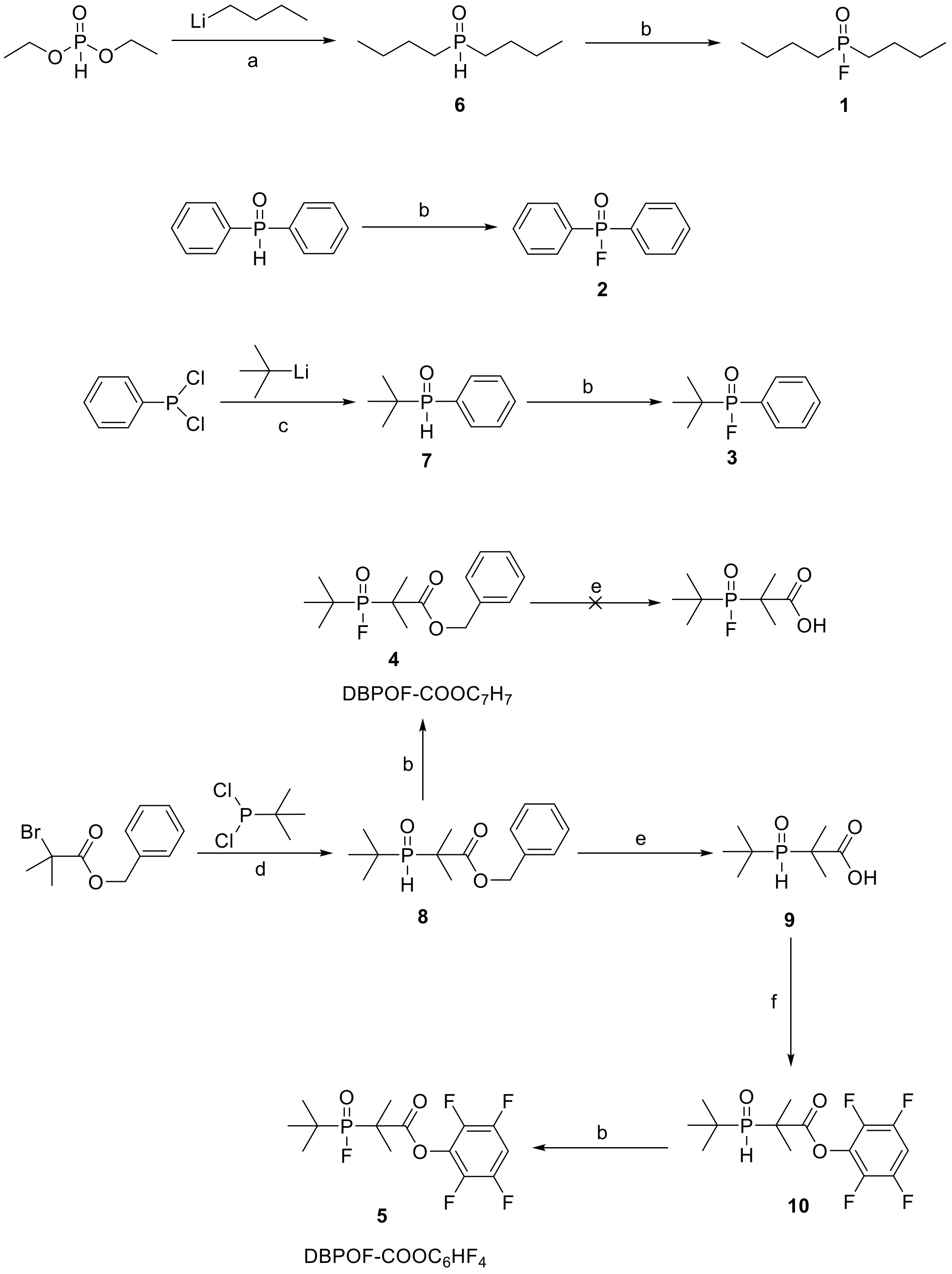

Supplementary Figure 1. Synthetic routes of the organofluorophosphine fluoride acceptors. Reagents and conditions: (a) tetrahydrofuran, -80 °C→RT, 5 h; (b) CsF, $CuCl_2$, acetone, RT, 10 h; (c) tetrahydrofuran, -80 °C→RT, overnight; (d) Zn, tetrahydrofuran, RT, 10 h; (e) H_2, Pd/C, MeOH, RT, 12 h; (f) DCC, DMAP, 2,3,5,6-tetrafluorophenol, tetrahydrofuran, RT, 12 h.

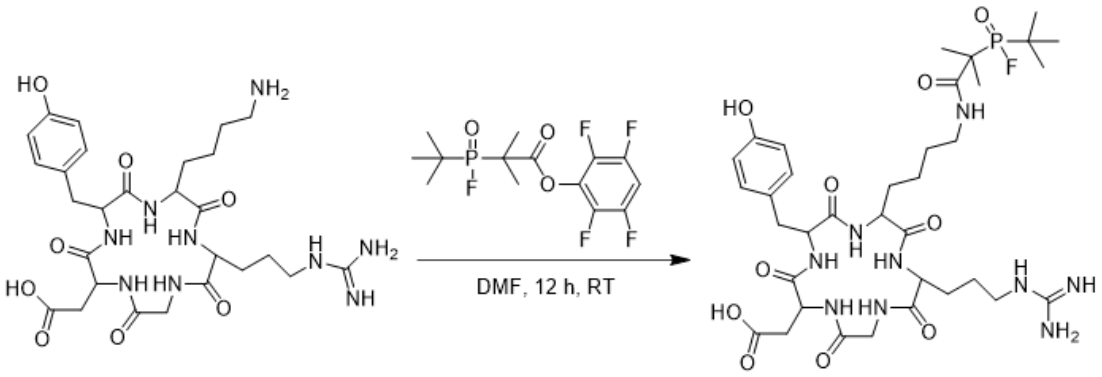

Supplementary Figure 2. Synthetic route of DBPOF-c(RGDyk). Reaction condition: anhydrous DMF, triethylamine, 12 h, RT.

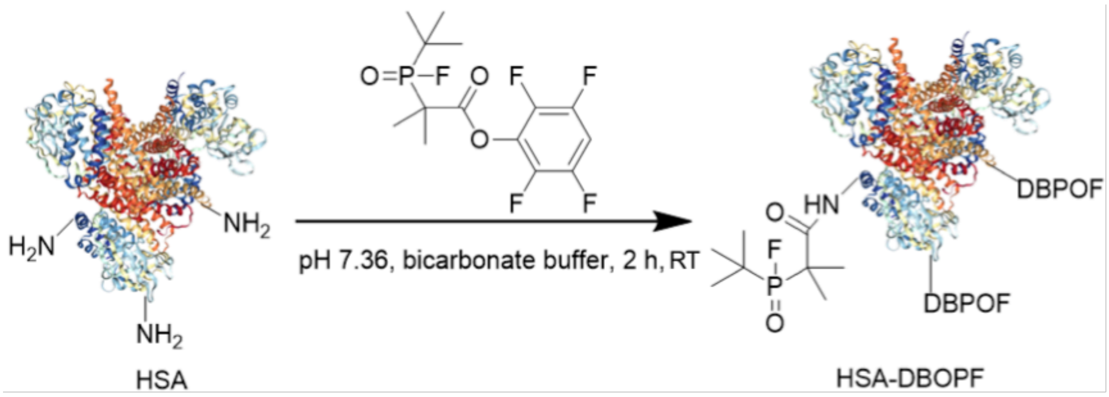

Supplementary Figure 3. Synthetic route of DBPOF-HSA. Reaction condition: 0.01 M sodium bicarbonate (pH 7.36), 2 h, RT.

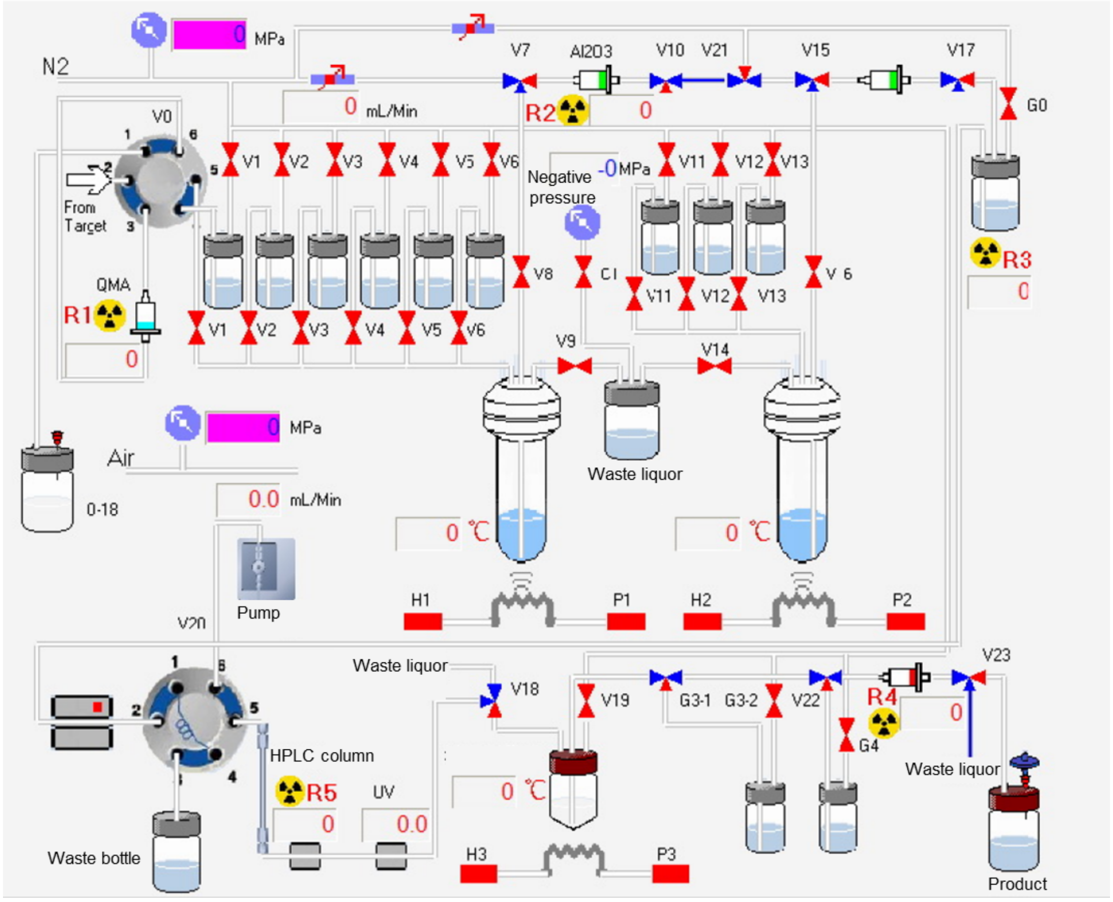

Supplementary Figure 4. Automated radiosynthesis module setup for ^{18}F-DBPOF-c(RGDyk). PET-MF-2V-IT-I module (Beijing PET Technology); V3, precursor DBPOF-c(RGDyk) [0.8-2.4 mg/0.5 mL solvent, DMSO/H2O = 1/1(v/v)]; V4, water (10.0 mL); V5, water (10.0 mL); V11, ethanol (1.0 mL); V12, empty bottle; product bottle: 0.9% sodium chloride for injection (9.0 mL); reaction temperature, 25 °C; starting activity, 0.6-1.2 Ci; reaction time, 15 min.

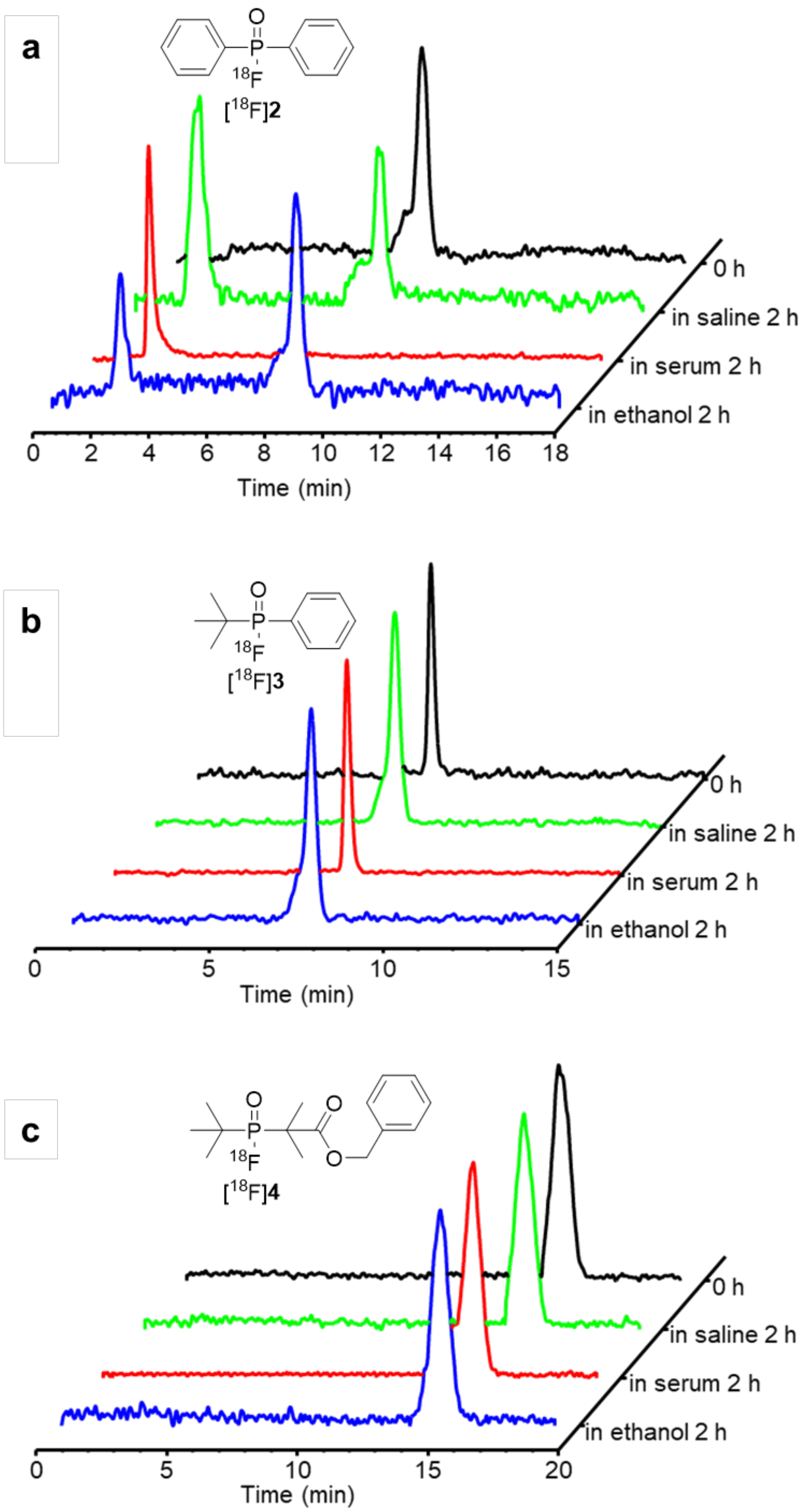
a
[18F]2
0 h
in saline 2 h
in serum 2 h
in ethanol 2 h
0 2 4 6 8 10 12 14 16 18
Time (min)
b
[18F]3
0 h
in saline 2 h
in serum 2 h
in ethanol 2 h
0 5 10 15
Time (min)
c
[18F]4
0 h
in saline 2 h
in serum 2 h
in ethanol 2 h
0 5 10 15 20
Time (min)

Supplementary Figure 5. *In vitro* stabilities of ^{18}F labeled organofluorophosphine fluoride acceptors. (**a**) Radio-HPLC analysis of [^{18}F]**2** after 2 h incubation in different media. (**b**) Radio-HPLC analysis of [^{18}F]**3** after 2 h incubation in different media. (**c**) Radio-HPLC analysis of [^{18}F]**4** after 2 h incubation in different media. Source data are provided as a Source Data file.

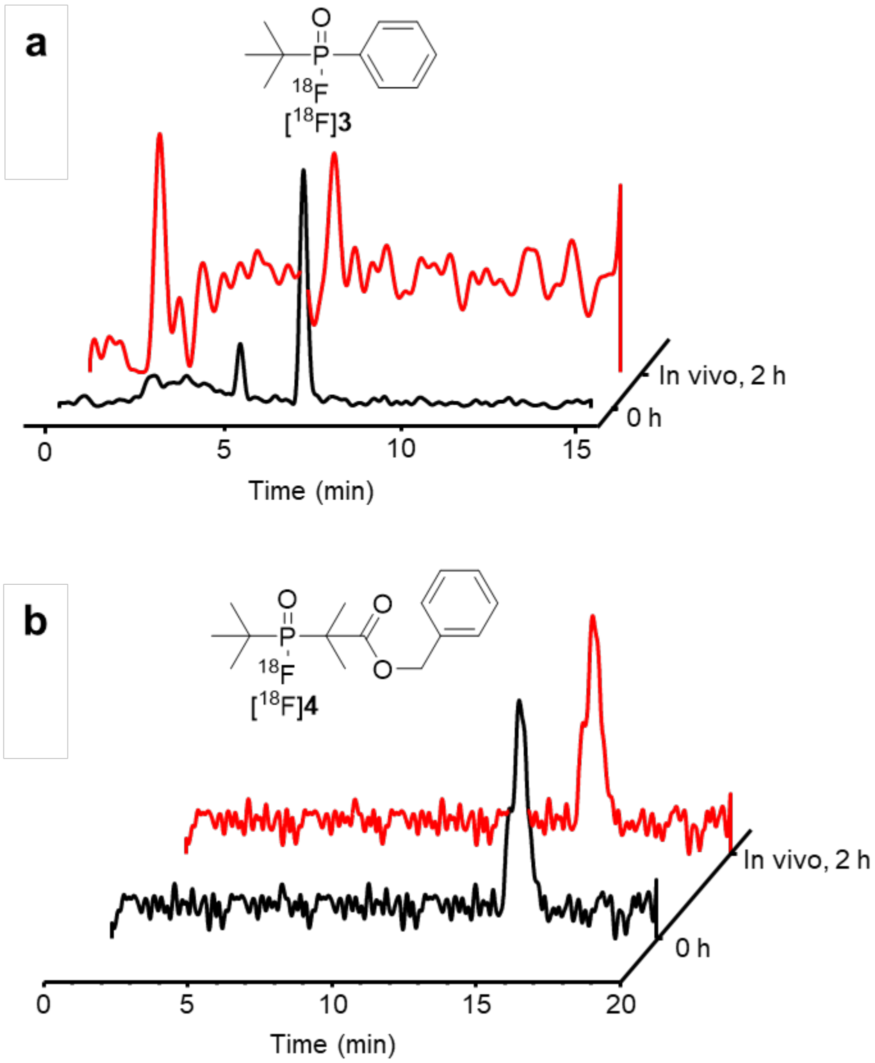

Supplementary Figure 6. Metabolic stability of ^{18}F labeled organofluorophosphine fluoride acceptors in normal ICR mice. Normal ICR mice were intravenously injected with [^{18}F]**3** or [^{18}F]**4** (2 mCi in 100 μL saline), respectively. The animals were sacrificed 2 h after injection. Blood was collected and treated with acetonitrile to precipitate insoluble proteins from the solution. The blood sample was immediately centrifuged for 5 min at 16, 543 xg. The supernatants were collected and passed through a 0.22 μm Millipore filter. Then 20 μL of the supernatants were analyzed by a radio-HPLC. (**a**) Radio-HPLC analysis of *in vivo* stabilities of [^{18}F]**3** 2 h post injection. (n = 3) (**b**) Radio-HPLC analysis of *in vivo* stabilities of [^{18}F]**4** 2 h post injection. (n = 3) Source data are provided as a Source Data file.

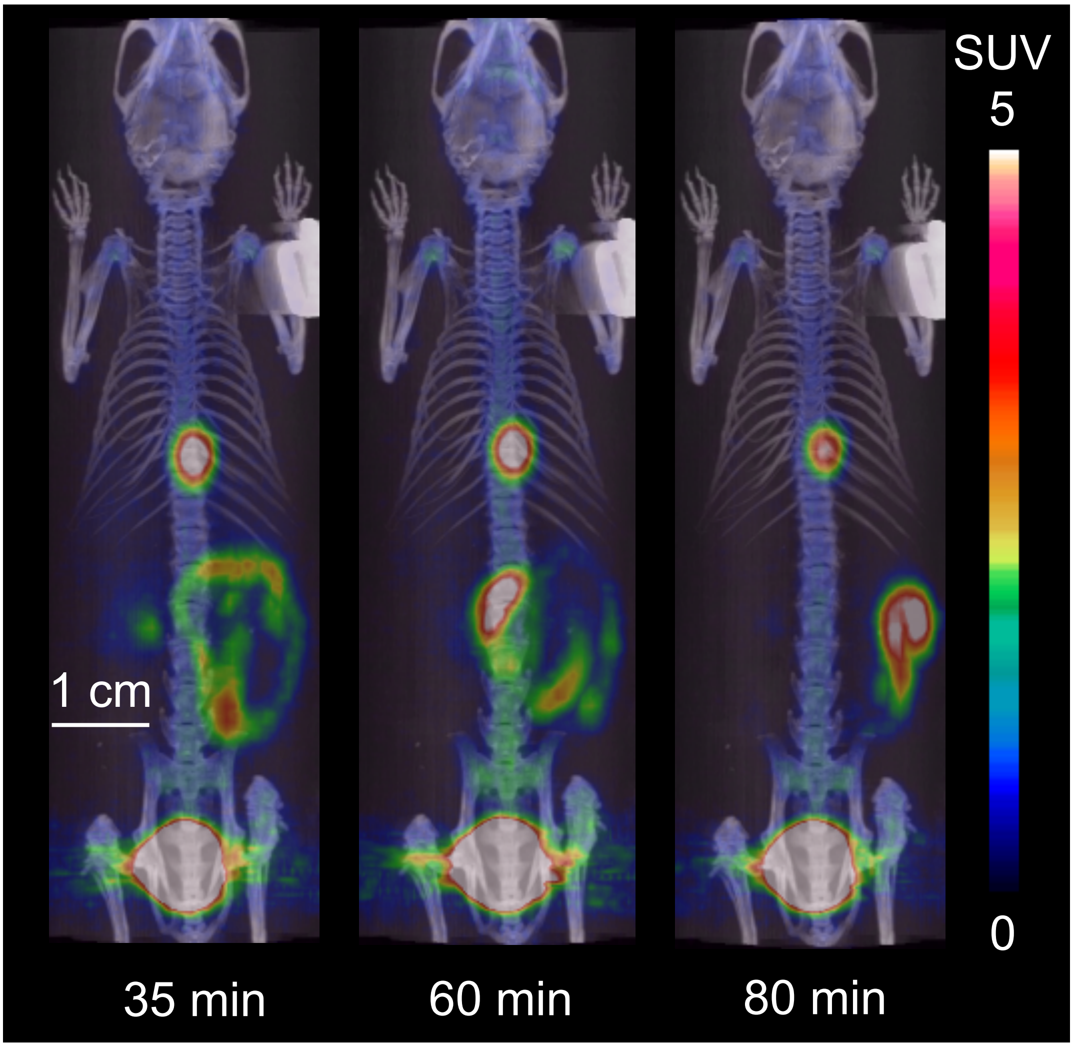

Supplementary Figure 7. MicroPET/CT analysis of *in vivo* stabilities of [^{18}F]**4** in normal ICR mice. The radiochemical purity of the [^{18}F]**4** was ~95%. (n = 3) The scale bar was set from zero to reflect the distribution of all signals clearly.

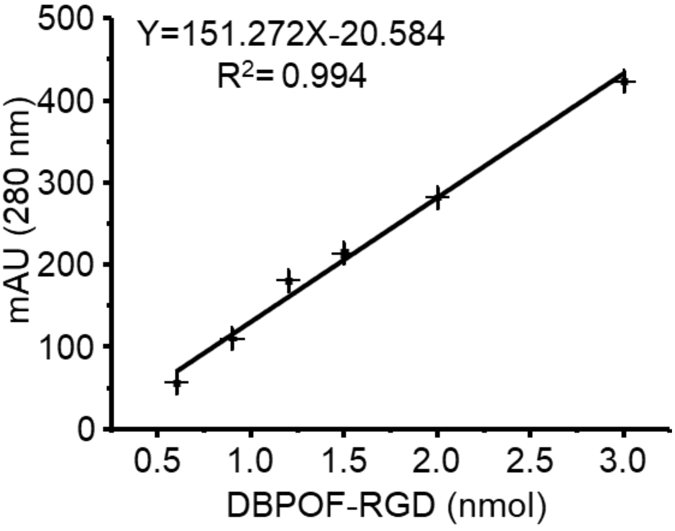

Supplementary Figure 8. UV standard curve of DBPOF-c(RGDyk). A linear standard curve was generated by correlating the HPLC UV signal (280 nm, mAU) and the injected amount of DBPOF-c(RGDyk) (pmol). The HPLC signal was obtained by injecting 600 (56 mAU), 900 (109 mAU), 1200 (184 mAU), 1500 (213 mAU), 2000 (281 mAU) and 3000 (423 mAU) pmol of DBPOF-c(RGDyk) into the HPLC (eluent A: H_2O, eluent B: acetonitrile, gradient started at 90% (v/v) A, ramping up to 80% B over 25 min then held constant throughout the run, flow rate: 1.0 mL/min, UV = 280 nm). UV absorption results are presented by means ± standard deviations for each concentration (n = 3). Source data are provided as a Source Data file.

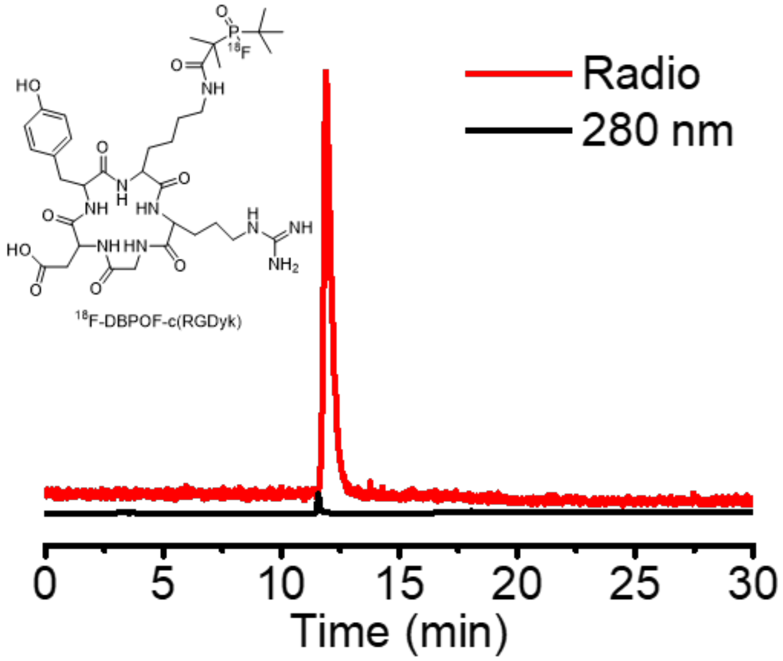

Supplementary Figure 9. Radio-HPLC analysis of RCP of ^{18}F-DBPOF-c(RGDyk). Samples were collected after radiolabeling and Sep-Pak C18 light cartridge (Waters, USA) purification. (n = 3)

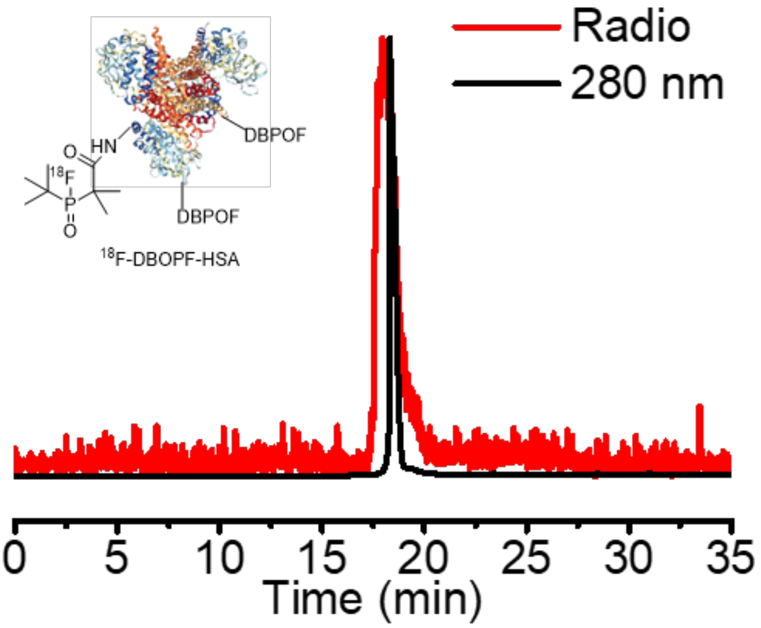

Supplementary Figure 10. Radio-HPLC analysis of RCP of ^{18}F-DBPOF-HSA. Samples were collected after radiolabeling and size exclusion chromatography (SEC) purification on a Xtimate SEC-300 column (Welch, China). (n = 3)

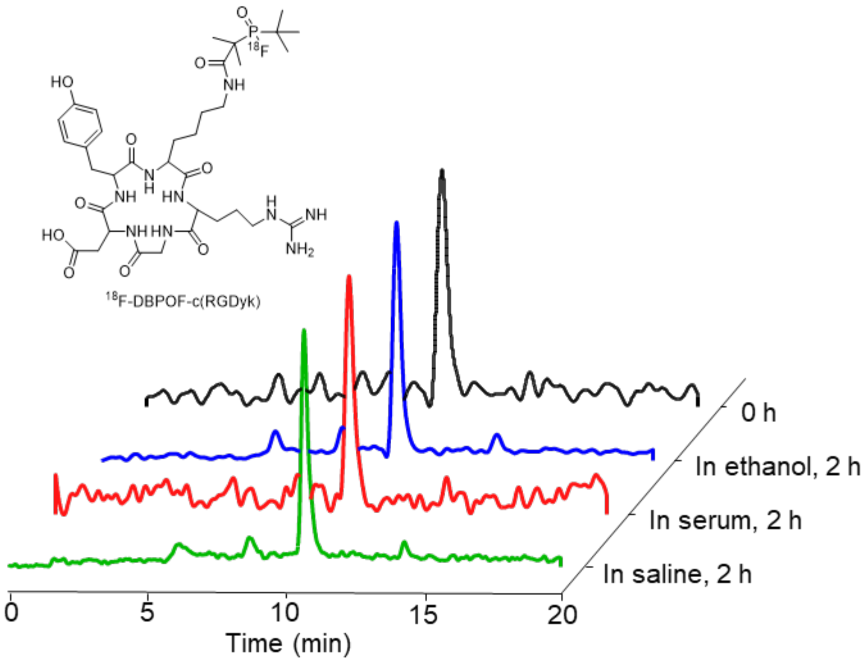

Supplementary Figure 11. *In vitro* stabilities of ^{18}F-DBPOF-c(RGDyk). Radio-HPLC analysis was performed after 2 h incubation in different media. (n = 3) Source data are provided as a Source Data file.

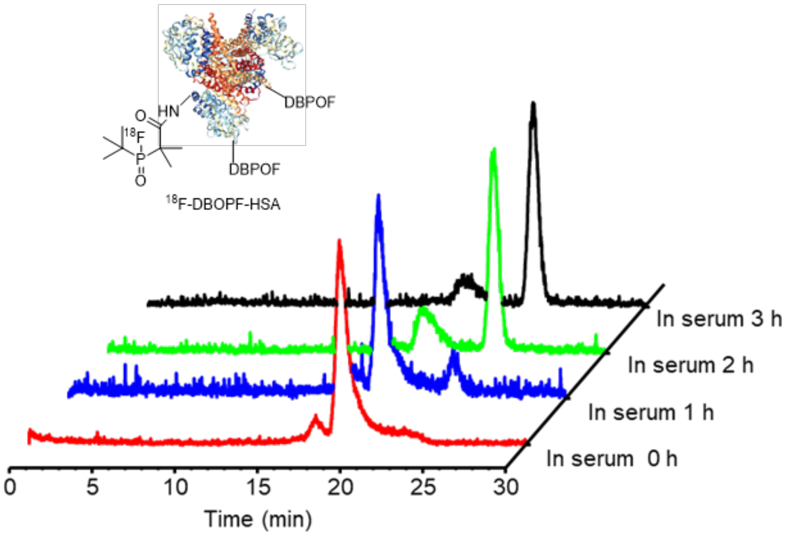

Supplementary Figure 12. *In vitro* stabilities of ^{18}F-DBPOF-HSA. Radio-HPLC analysis was performed after 2 h incubation in different media. (n = 3) Source data are provided as a Source Data file.

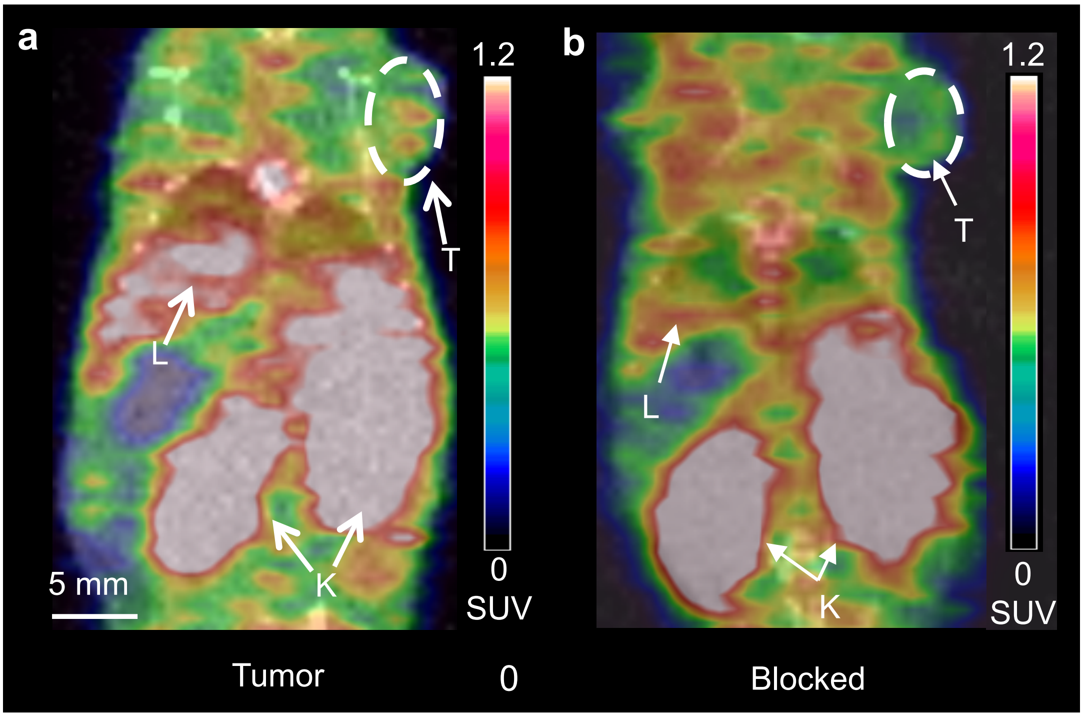

Supplementary Figure 13**.** MicroPET/CT imaging of the mice bearing U87MG tumor. (**a**) A PET image from a dynamic scan with ^{18}F-DBPOF-c(RGDyk) in U87MG nude mice bearing gliomablastoma, reconstructed at 23 min post-injection with the tumor (T), live (L), and kidney (K) indicated by white arrows. (**b**) Control group showed no specific tumor uptake indicated by a white arrow. (n = 3)

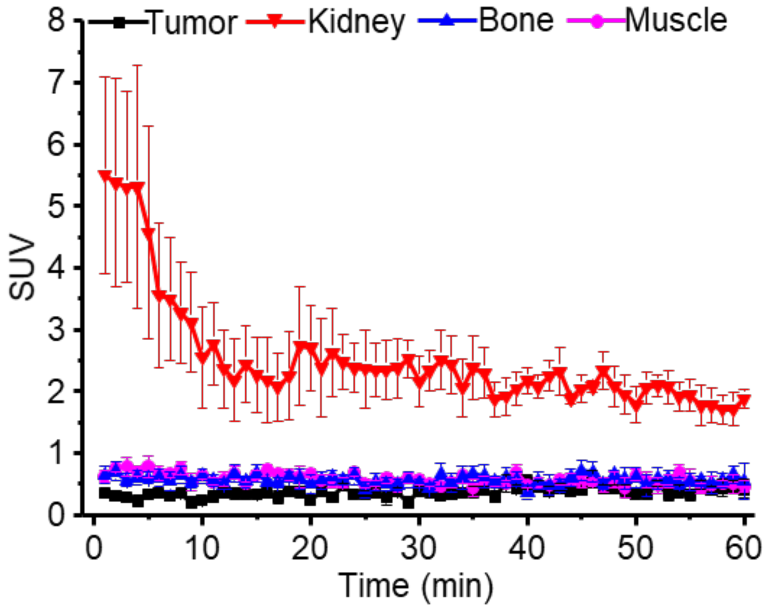

Supplementary Figure 14. Time-activity curves of ^{18}F-DBPOF-c(RGDyk). Uptakes in the tumor, kidney, bone, and muscle in the control group are demonstrated. Results of time-activity curves of ^{8}F-DBPOF-c(RGDyk) are presented by means ± stardard diveations (n = 3). Source data are provided as a Source Data file.

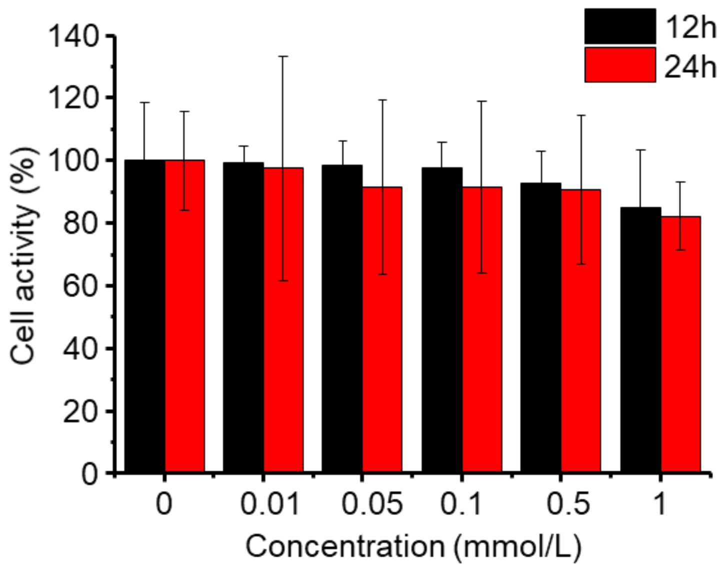

Supplementary Figure 15. Cell cytotoxic analysis of DBPOF-c(RGDyk) in U87MG cell. DBPOF-c(RGDyk) exhibited very low cytotoxicity even at a relatively high concentration of 1.0 mmol/L. Results are presented by means ± standard deviations (n = 3). Source data are provided as a Source Data file.

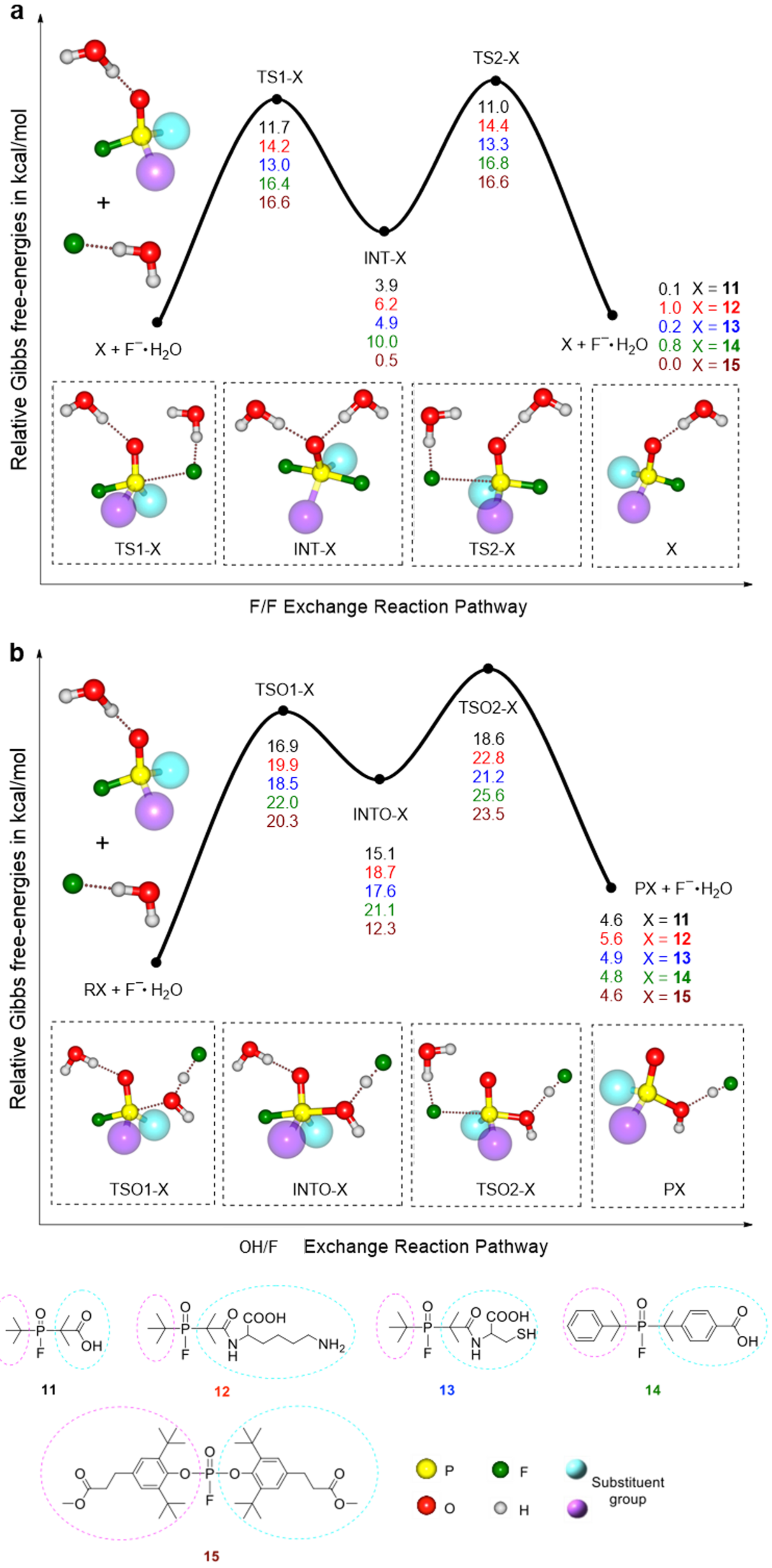
a
Relative Gibbs free-energies in kcal/mol
TS1-X
11.7
14.2
13.0
16.4
16.6
TS2-X
11.0
14.4
13.3
16.8
16.6
INT-X
3.9
6.2
4.9
10.0
0.5
X + F⁻•H2O
X + F⁻•H2O
0.1 X = 11
1.0 X = 12
0.2 X = 13
0.8 X = 14
0.0 X = 15
TS1-X
INT-X
TS2-X
X
F/F Exchange Reaction Pathway
b
Relative Gibbs free-energies in kcal/mol
TSO1-X
16.9
19.9
18.5
22.0
20.3
TSO2-X
18.6
22.8
21.2
25.6
23.5
INTO-X
15.1
18.7
17.6
21.1
12.3
RX + F⁻• H2O
PX + F⁻•H2O
4.6 X = 11
5.6 X = 12
4.9 X = 13
4.8 X = 14
4.6 X = 15
TSO1-X
INTO-X
TSO2-X
PX
OH/F Exchange Reaction Pathway
11
12
13
14
15
P
F
O
H
Substituent group

Supplementary Figure 16. Reaction pathways and free-energy (kcal/mol) profiles. (**a**) the F/F isotopic and (**b**) OH/F exchange processes of five selected reactant systems. Geometries were optimized at B3LYP/6-31+G*[1] level of theory. Single point calculations were performed at CAM-B3LYP/6-311++G**[2] level of theory. Two substituent groups on the phosphorus center were simplified as two big spheres in the molecular structures for the sake of clarity.

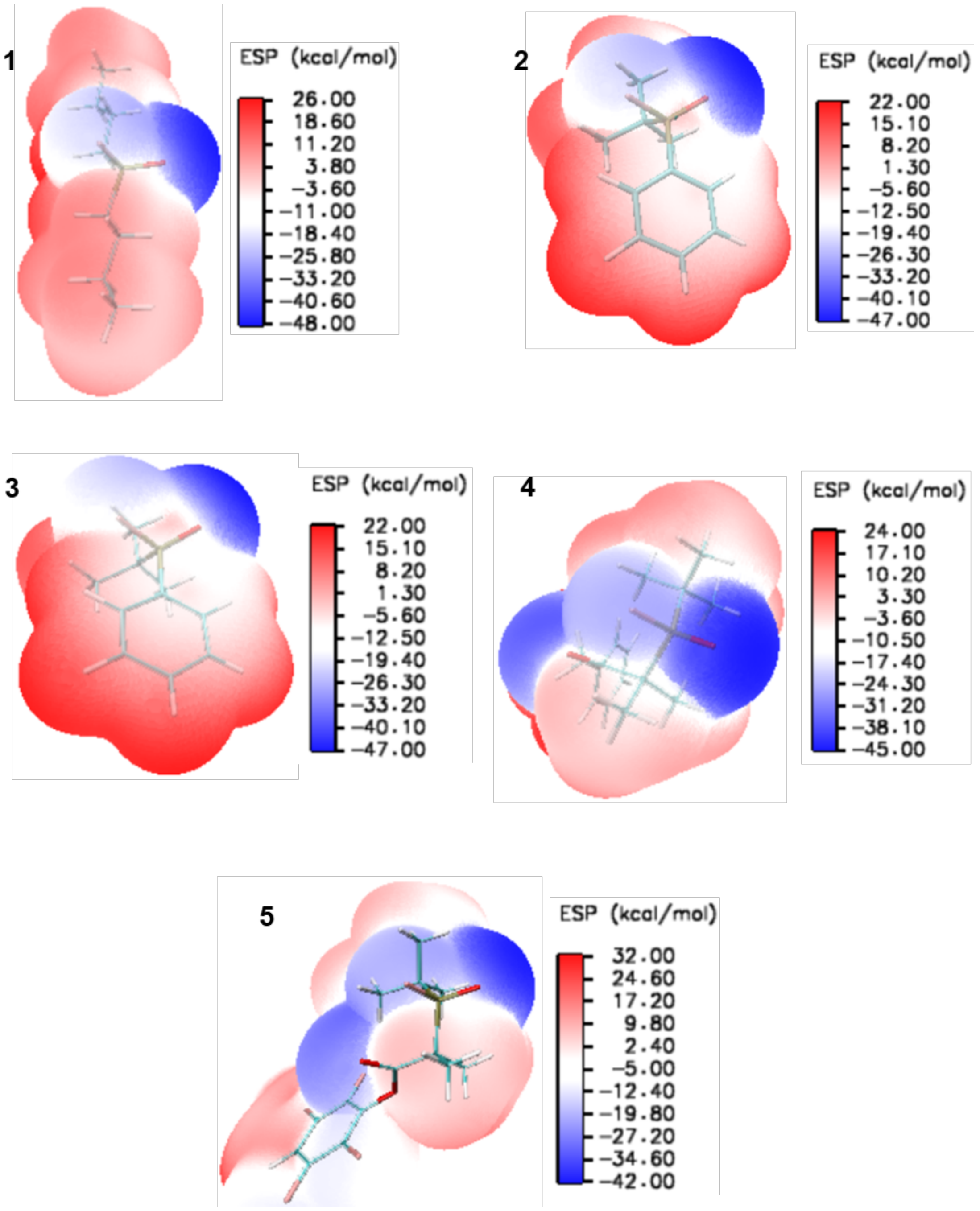

Supplementary Figure 17. Charge distribution of organofluorophosphine fluoride acceptors **1**-**5**.

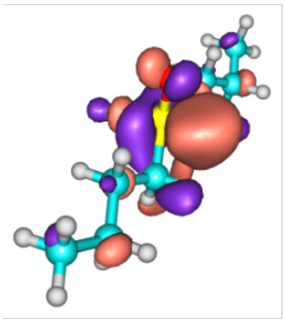

1 0.0386

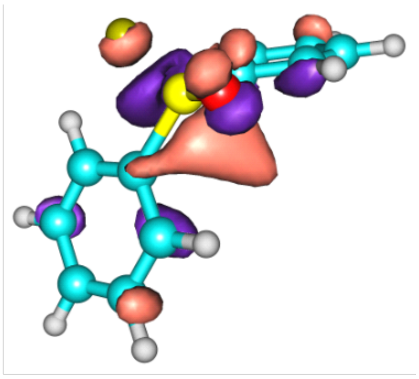

2 0.0315

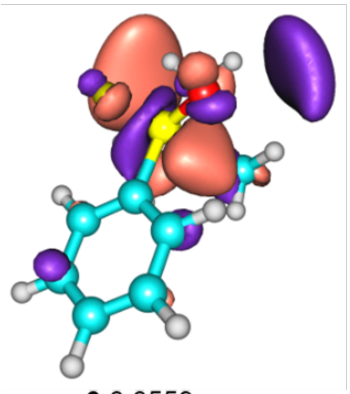

3 0.0559

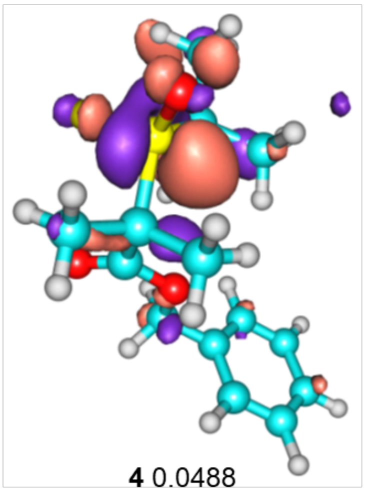

4 0.0488

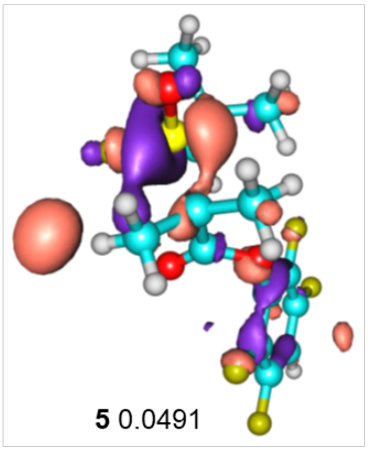

5 0.0491

Supplementary Figure 18. Delocalized molecular orbital of organofluorophosphine fluoride acceptors **1**-**5**.

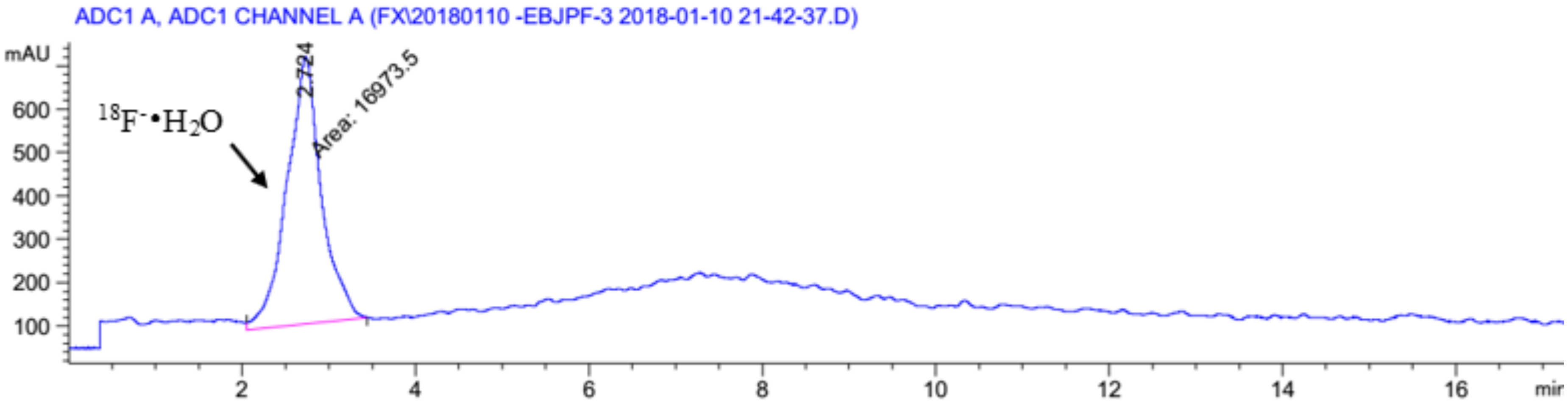

Supplementary Figure 19. Radio-HPLC analysis of RCY of [^{18}F]**2** by labeling method II, RCY = 0%.

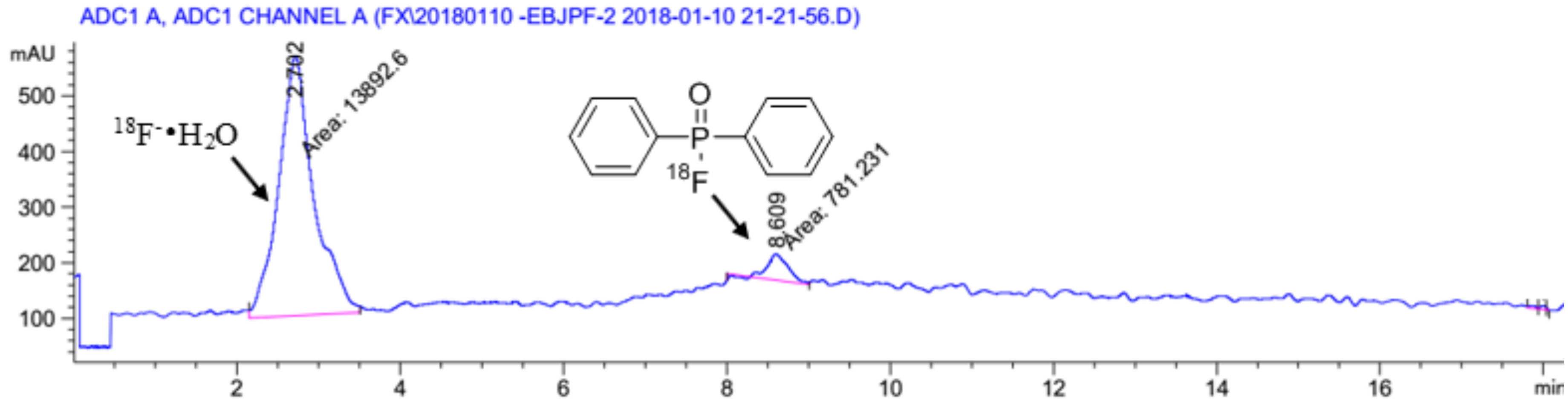

Supplementary Figure 20. Radio-HPLC analysis of RCY of [^{18}F]**2** by labeling method IV, RCY = 5%.

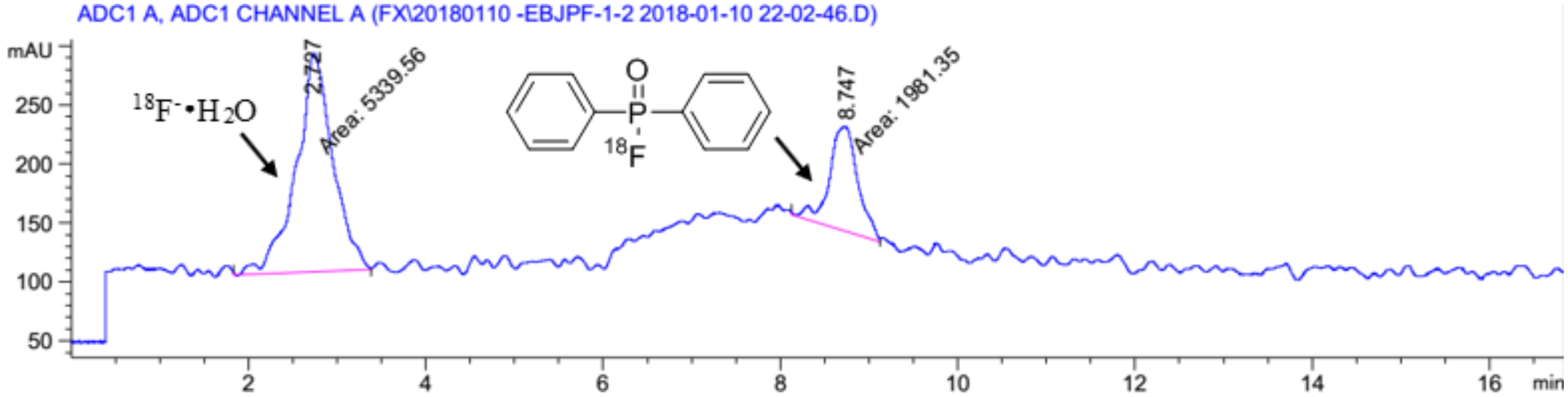

Supplementary Figure 21. Radio-HPLC analysis of RCY of [^{18}F]**2** by labeling method VI, RCY = 27%.

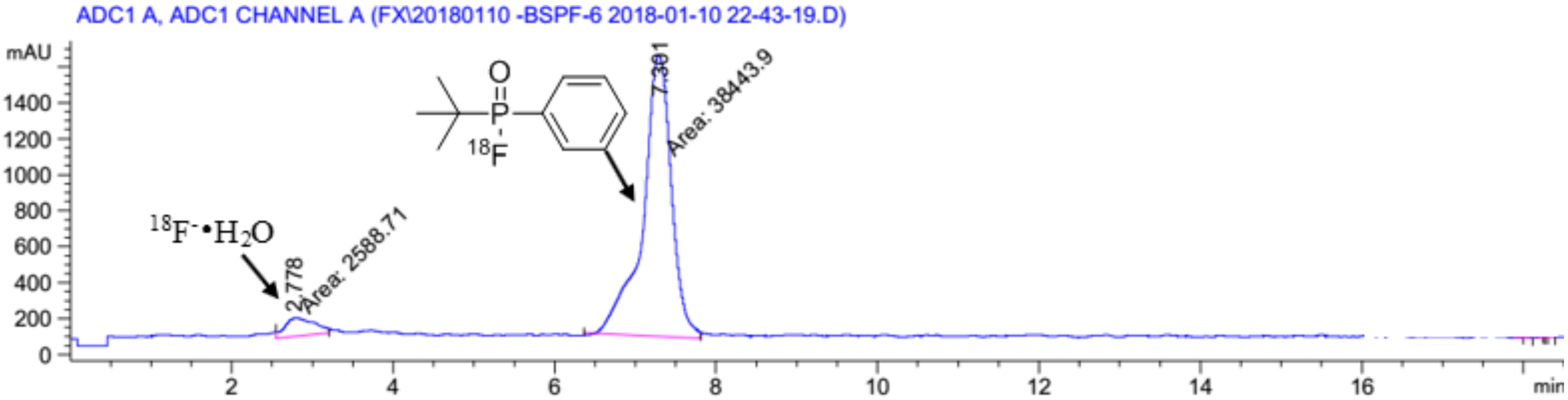

Supplementary Figure 22. Radio-HPLC analysis of RCY of [^{18}F]**3** by labeling method II, RCY = 93%.

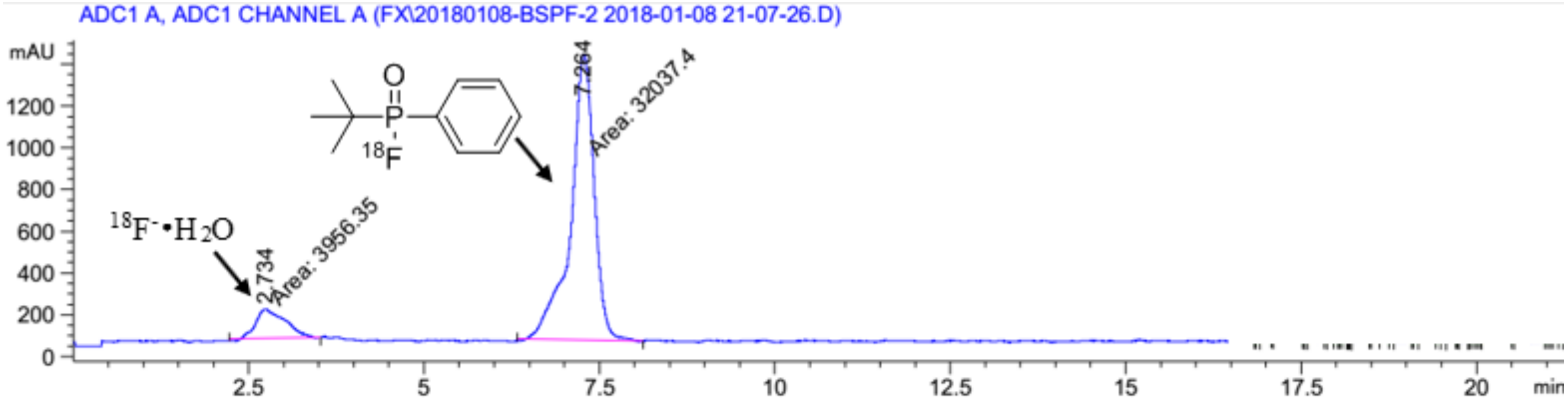

Supplementary Figure 23. Radio-HPLC analysis of RCY of [^{18}F]**3** by labeling method IV, RCY = 89%.

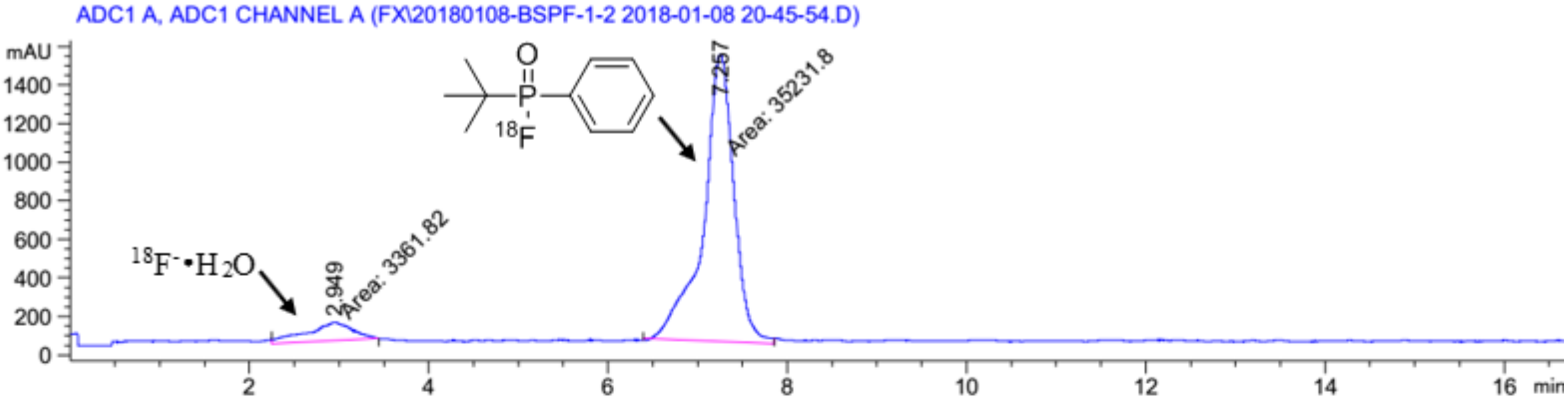

Supplementary Figure 24. Radio-HPLC analysis of RCY of [^{18}F]**3** by labeling method VI, RCY = 92%.

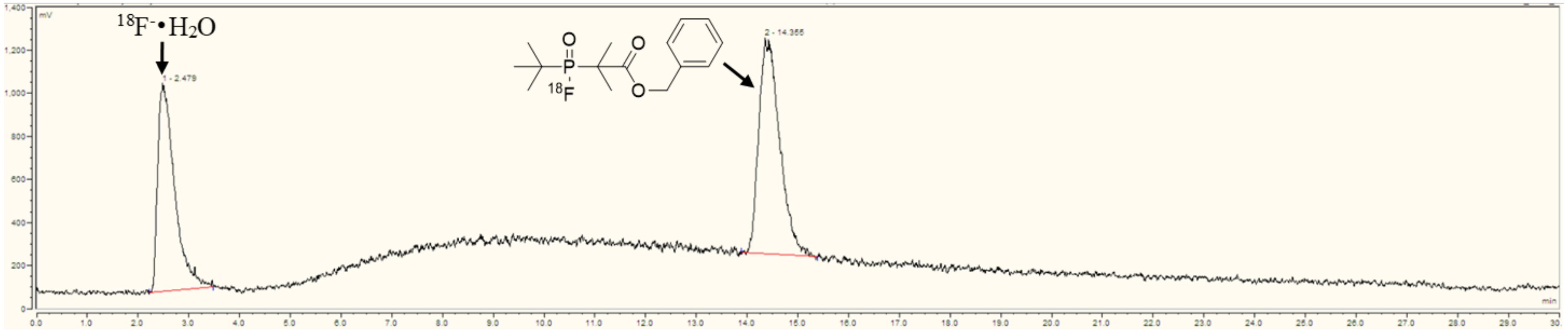

Supplementary Figure 25. Radio-HPLC analysis of RCY of [^{18}F]**4** by labeling method I, RCY = 56%.

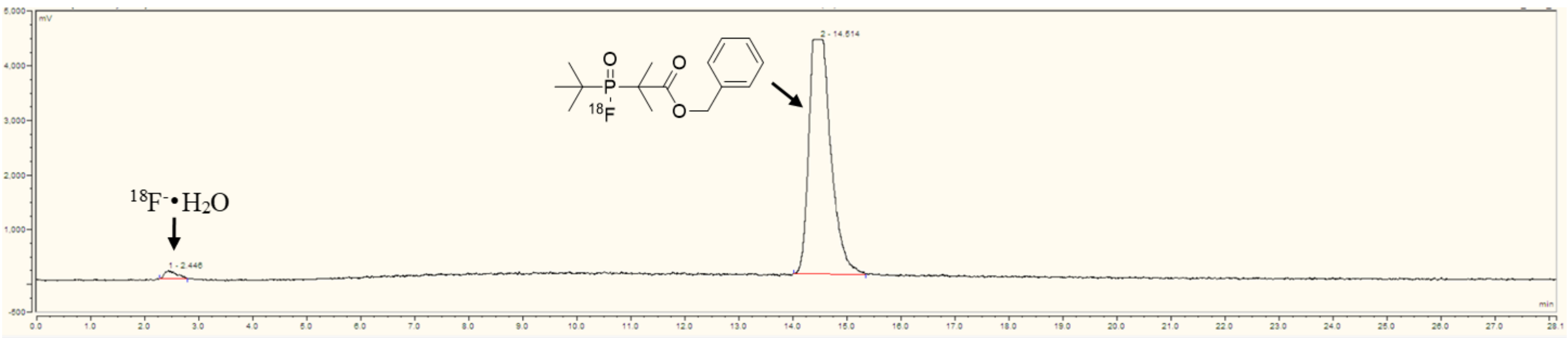

Supplementary Figure 26. Radio-HPLC analysis of RCY of [^{18}F]**4** by labeling method II, RCY = 98%.

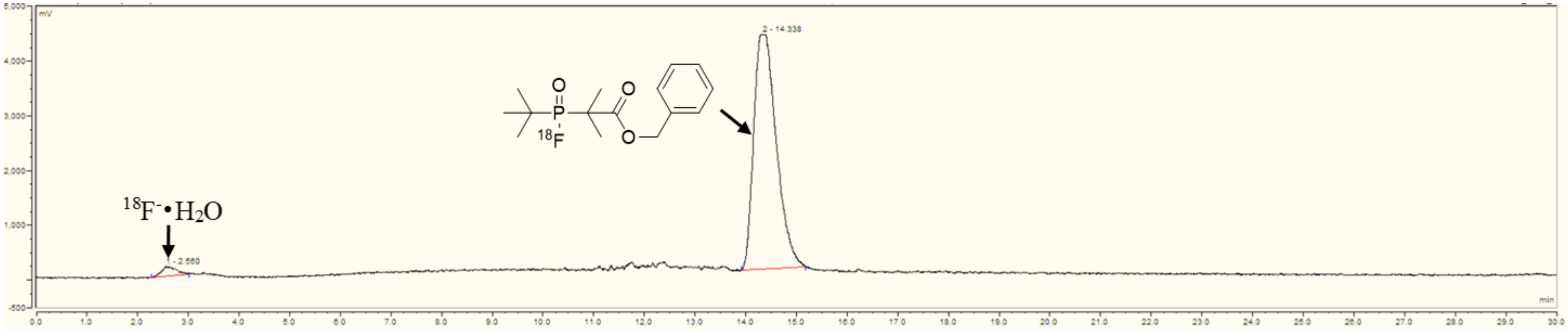

Supplementary Figure 27. Radio-HPLC analysis of RCY of [^{18}F]**4** by labeling method III, RCY = 95%.

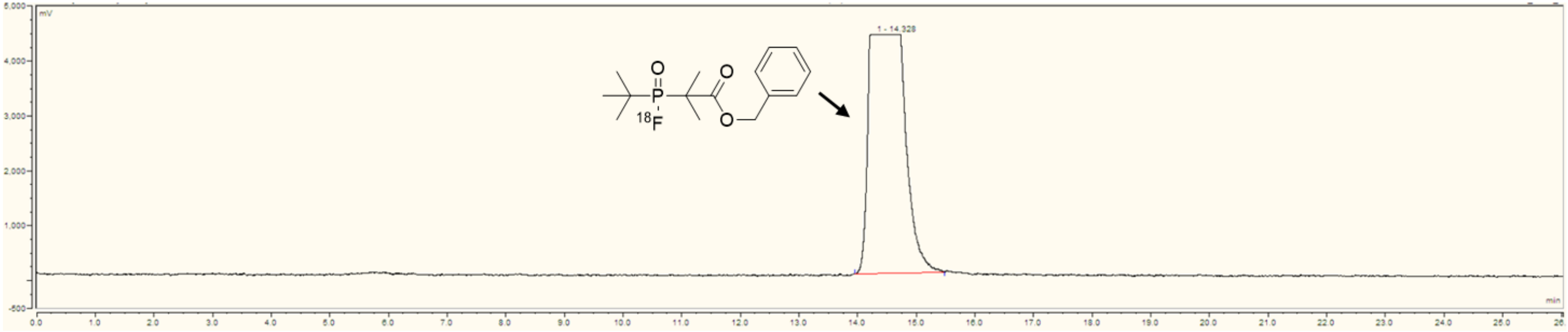

Supplementary Figure 28. Radio-HPLC analysis of RCY of [^{18}F]**4** by labeling method IV, RCY = 100%.

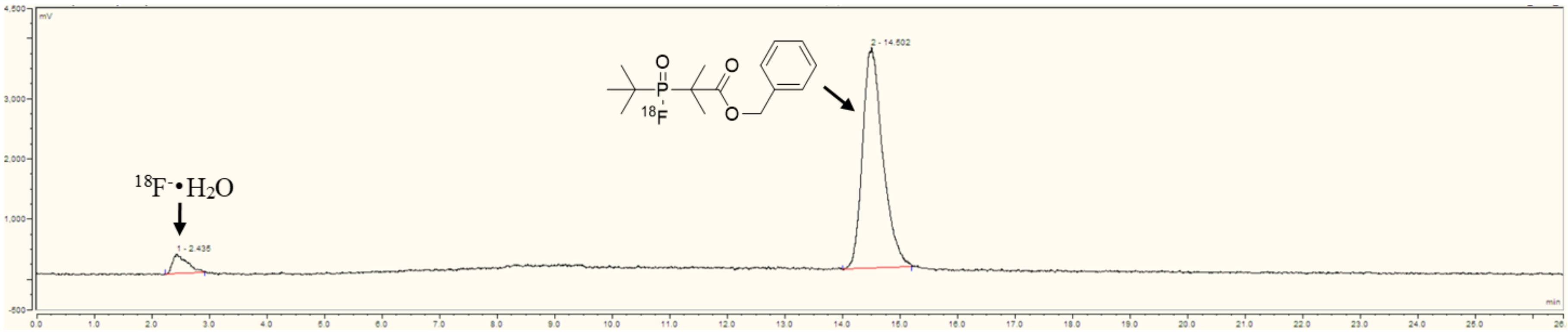

Supplementary Figure 29. Radio-HPLC analysis of RCY of [^{18}F]**4** by labeling method V, RCY = 95%.

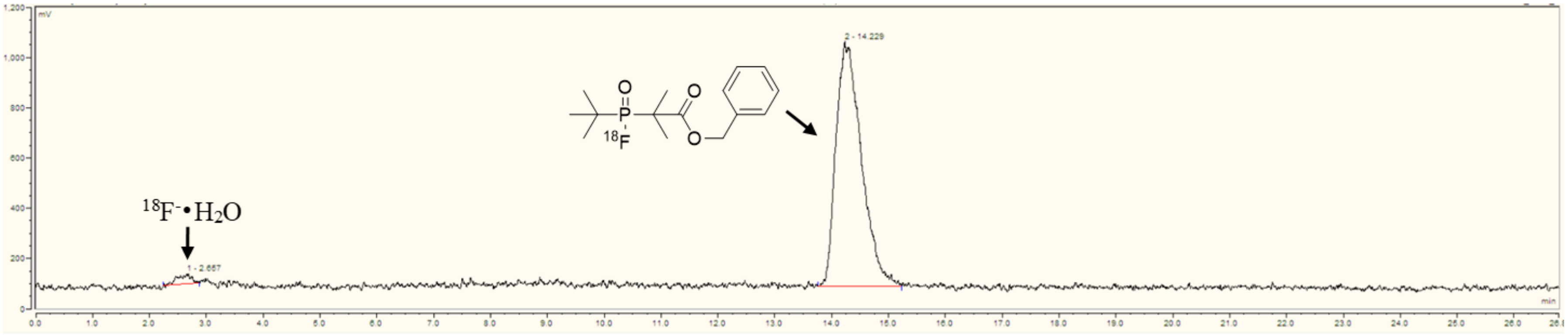

Supplementary Figure 30. Radio-HPLC analysis of RCY of [^{18}F]**4** by labeling method VI, RCY = 98%.

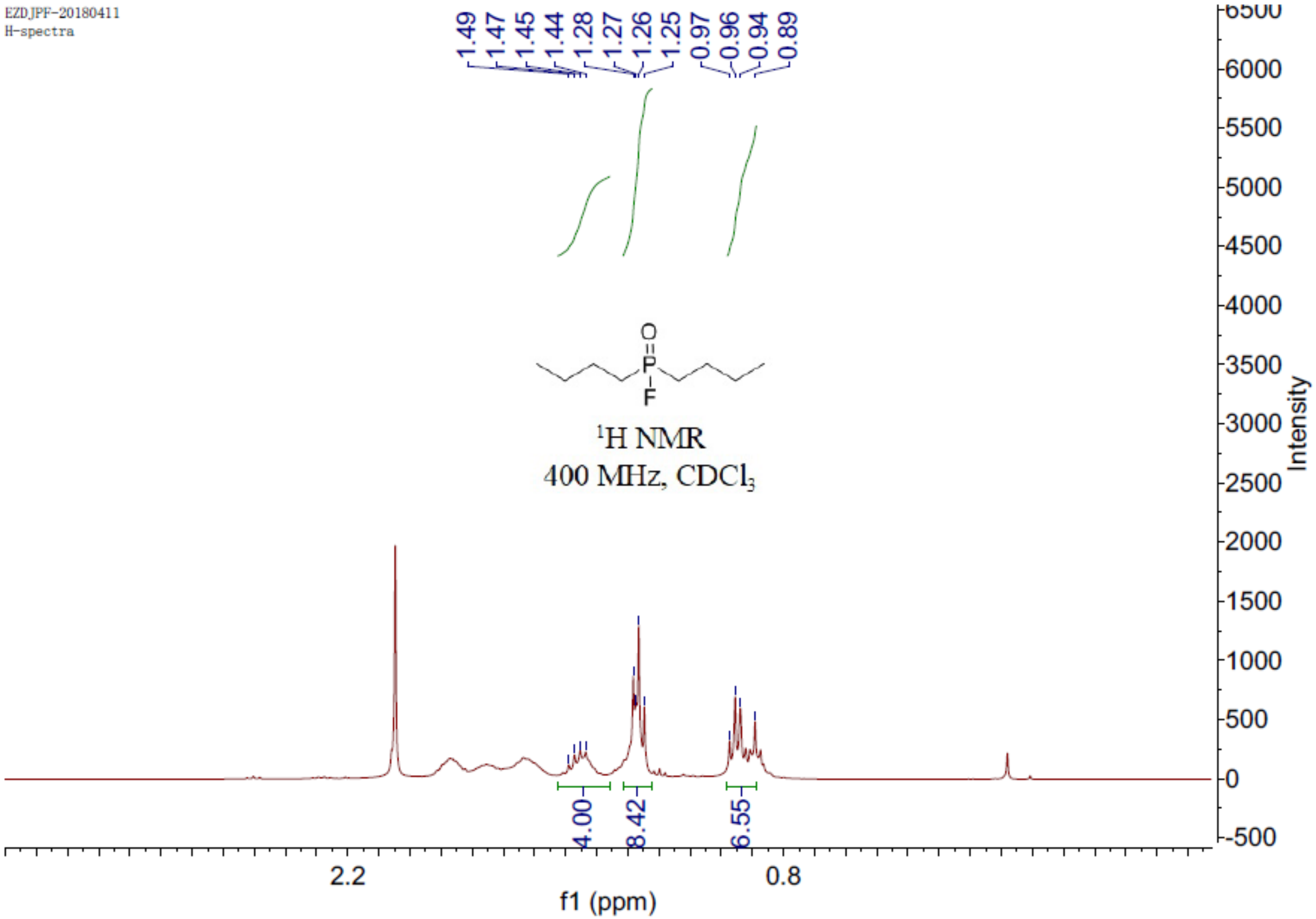

Supplementary Figure 31. ^{1}H NMR spectrum of **1**.

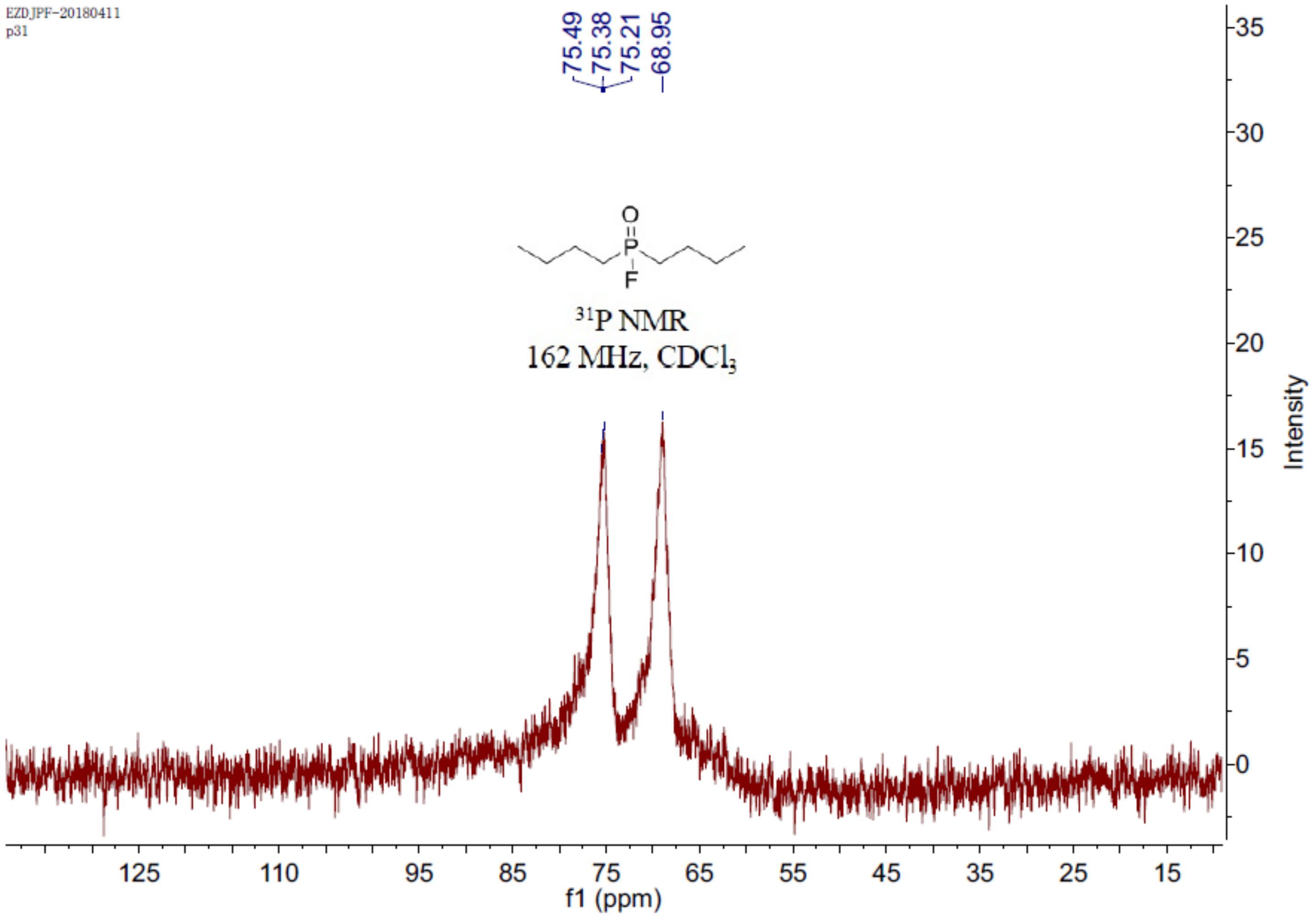

Supplementary Figure 32. ^{31}P NMR spectrum of **1**.

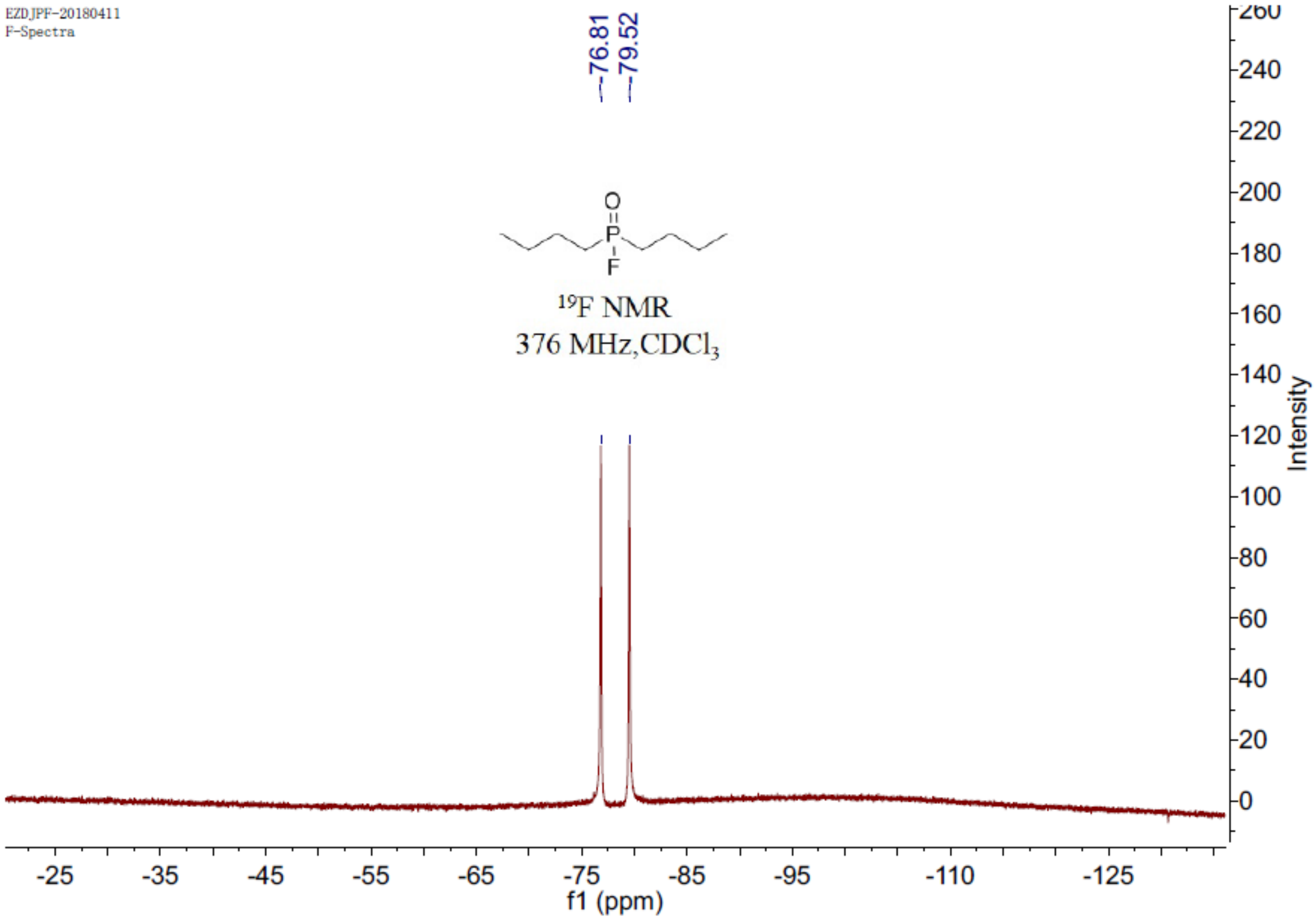

Supplementary Figure 33. ^{19}F NMR spectrum of **1**.

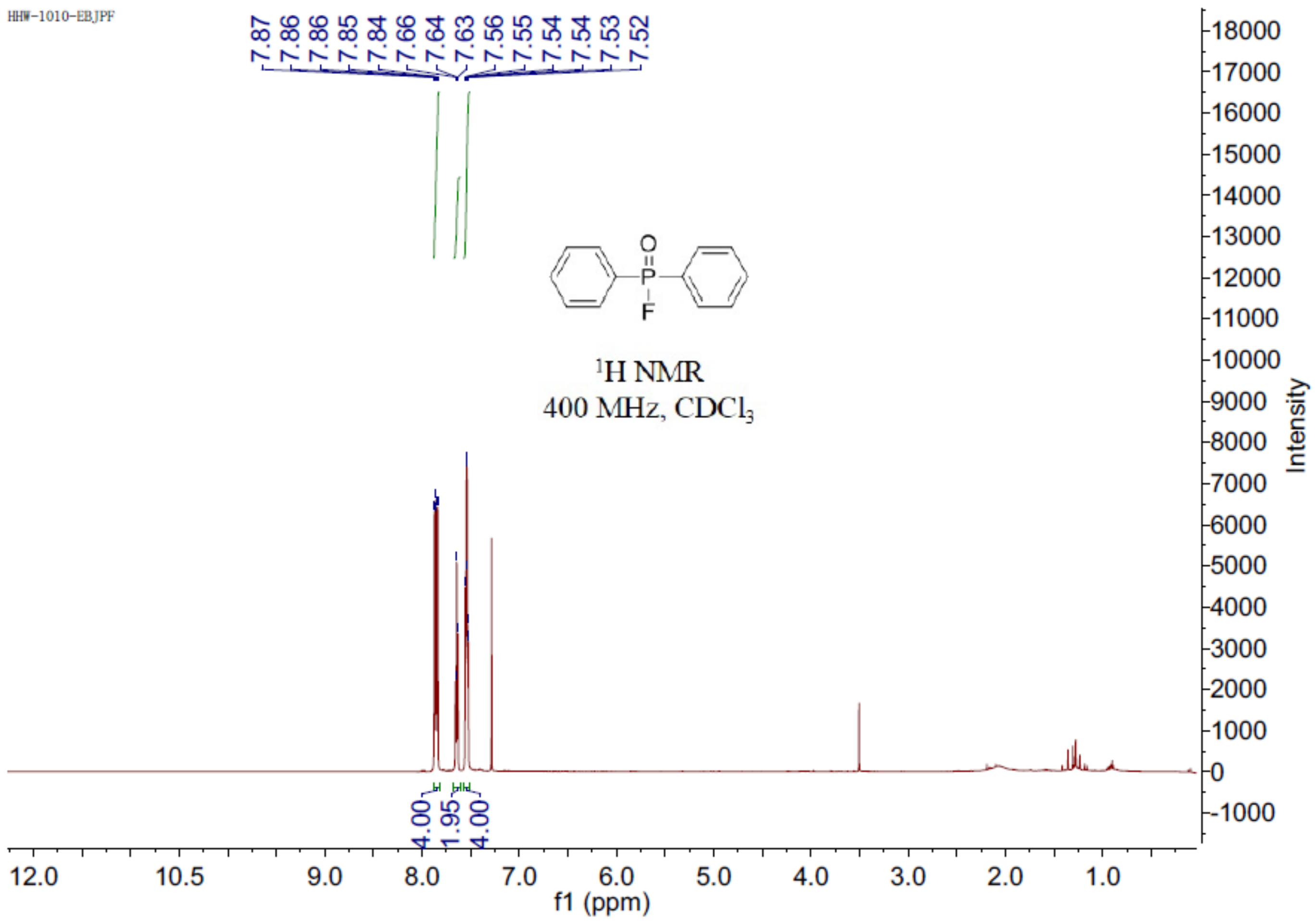

Supplementary Figure 34. ^{1}H NMR spectrum of **2**.

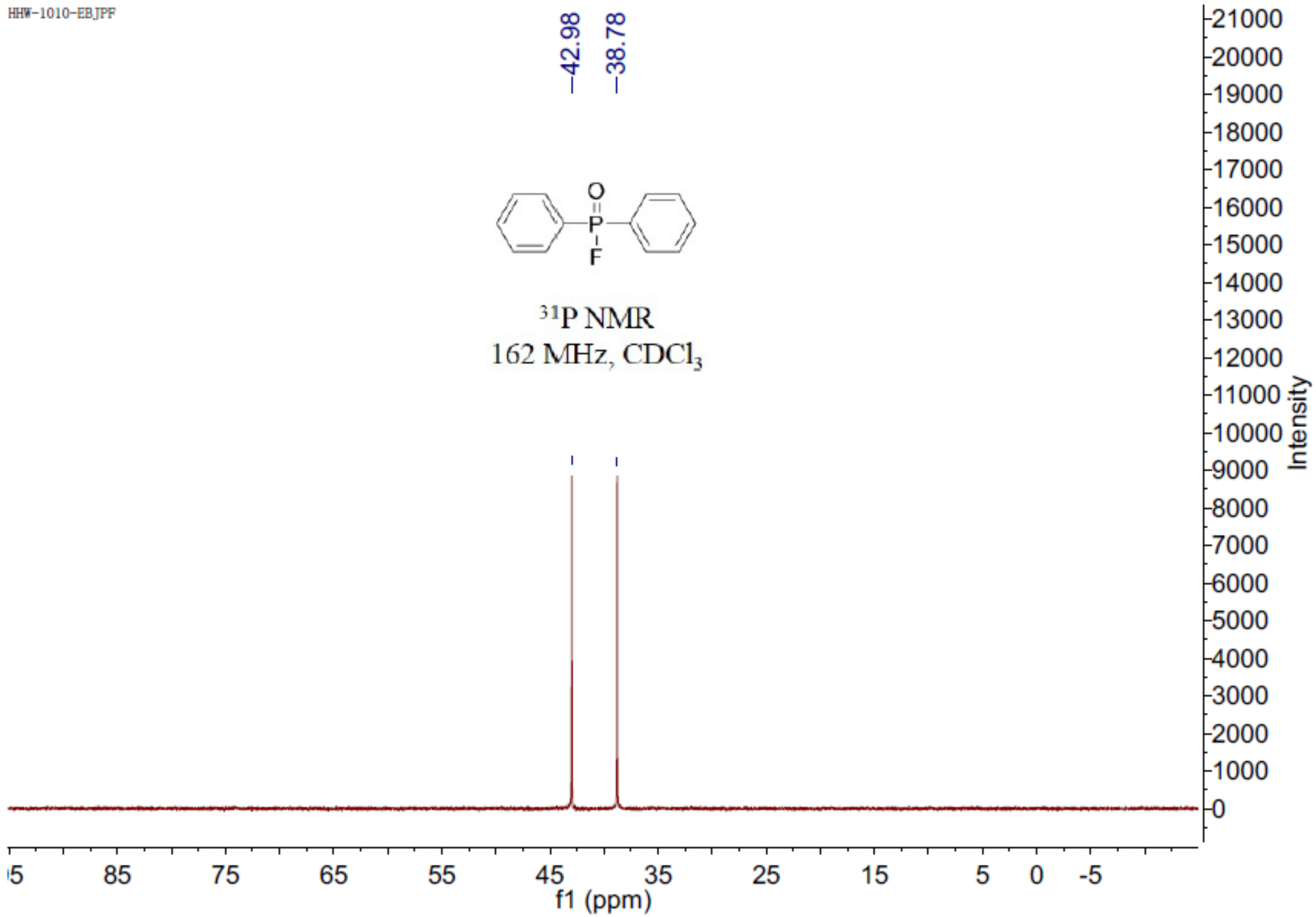

Supplementary Figure 35. ^{31}P NMR spectrum of **2**.

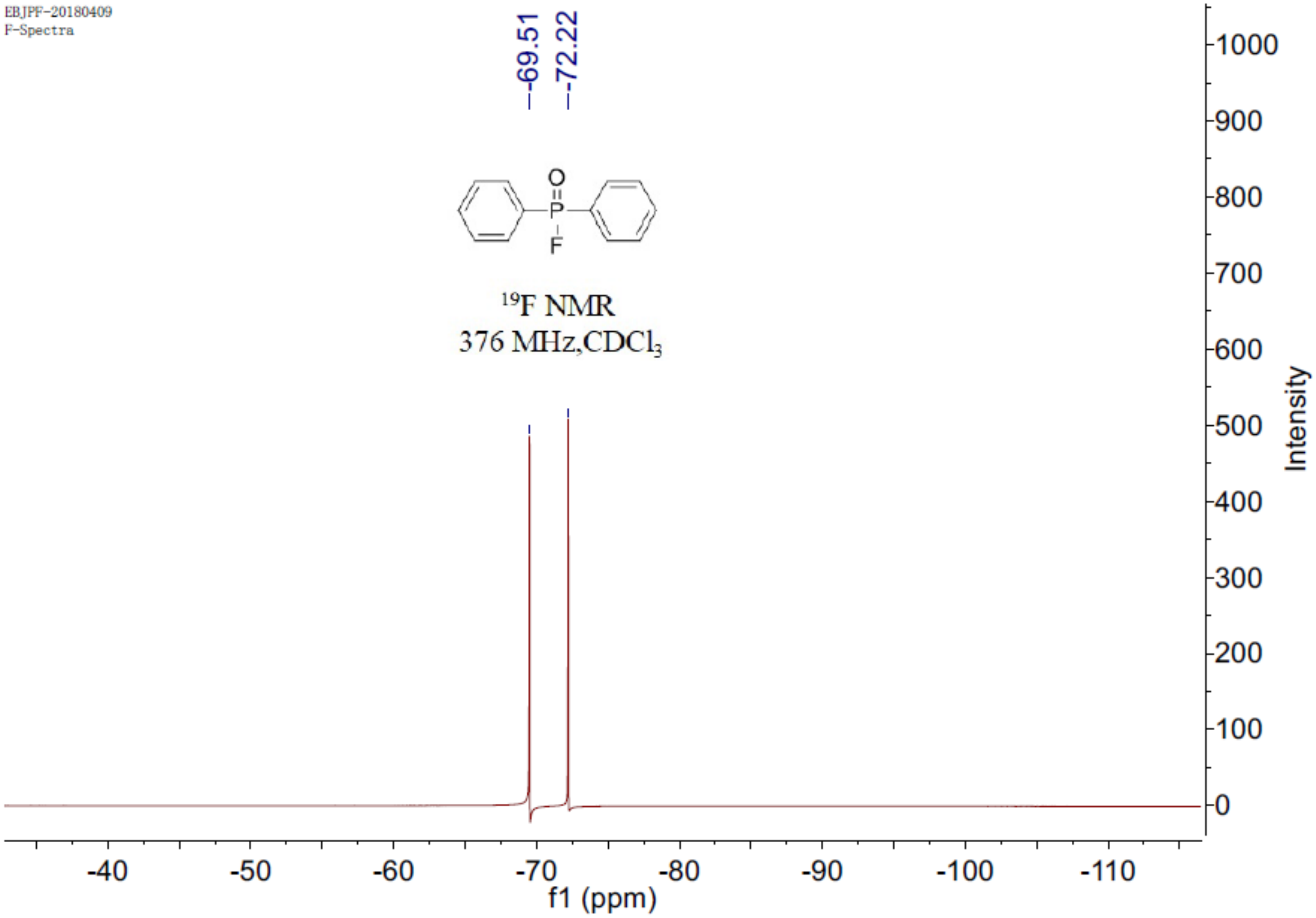

Supplementary Figure 36. ^{19}F NMR spectrum of **2**.

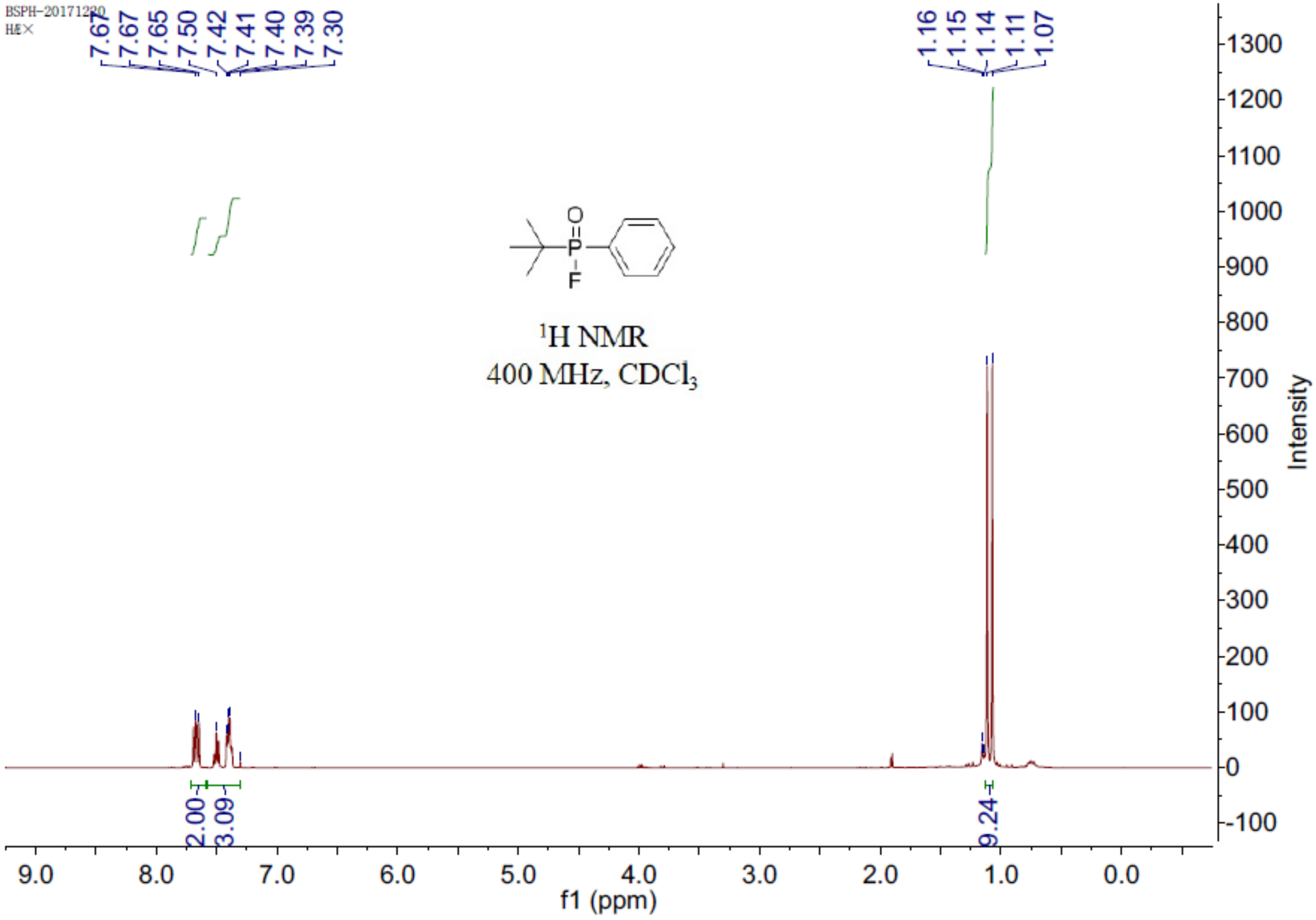

Supplementary Figure 37. ^{1}H NMR spectrum of **3**.

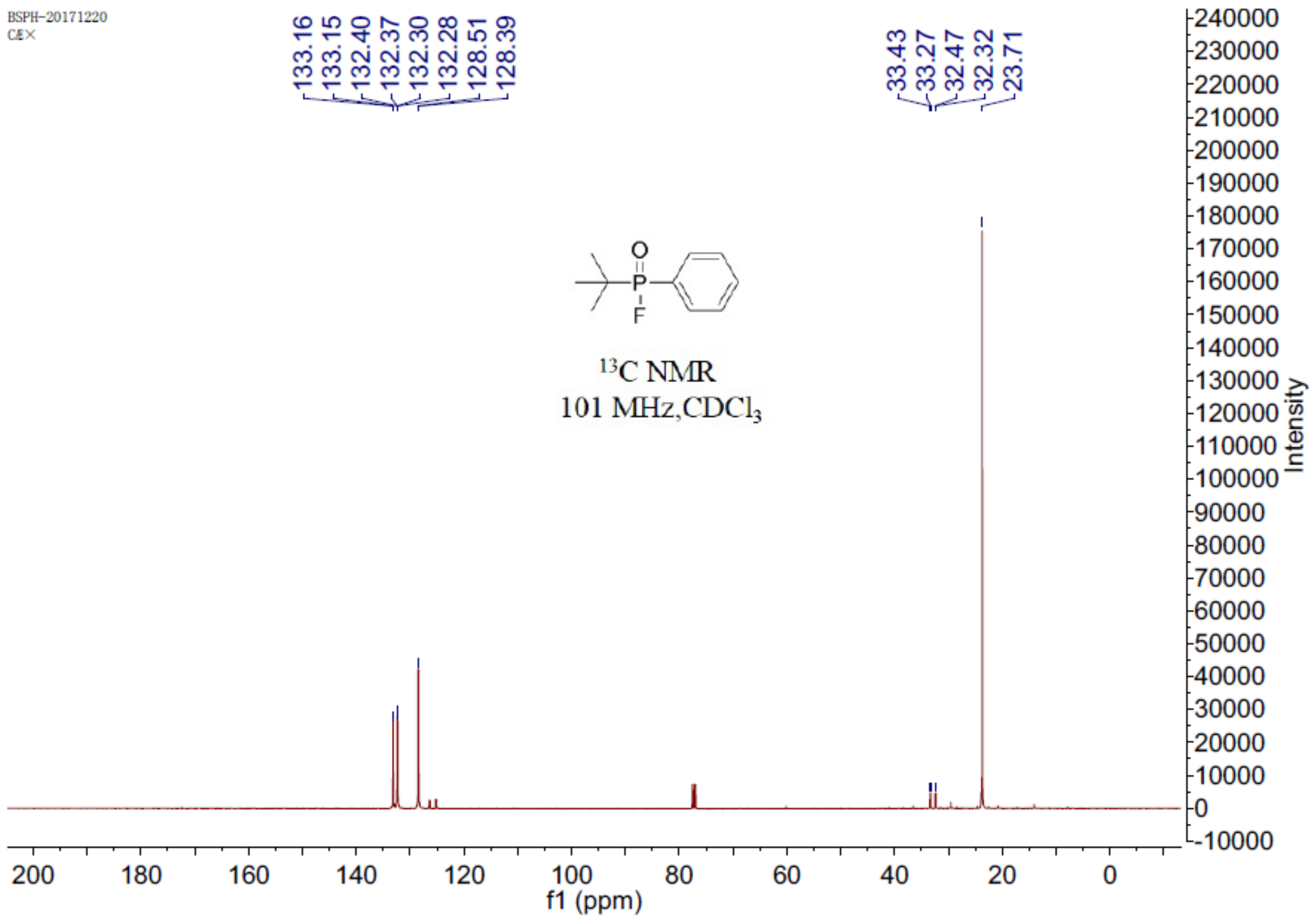

Supplementary Figure 38. ^{13}C NMR spectrum of **3**.

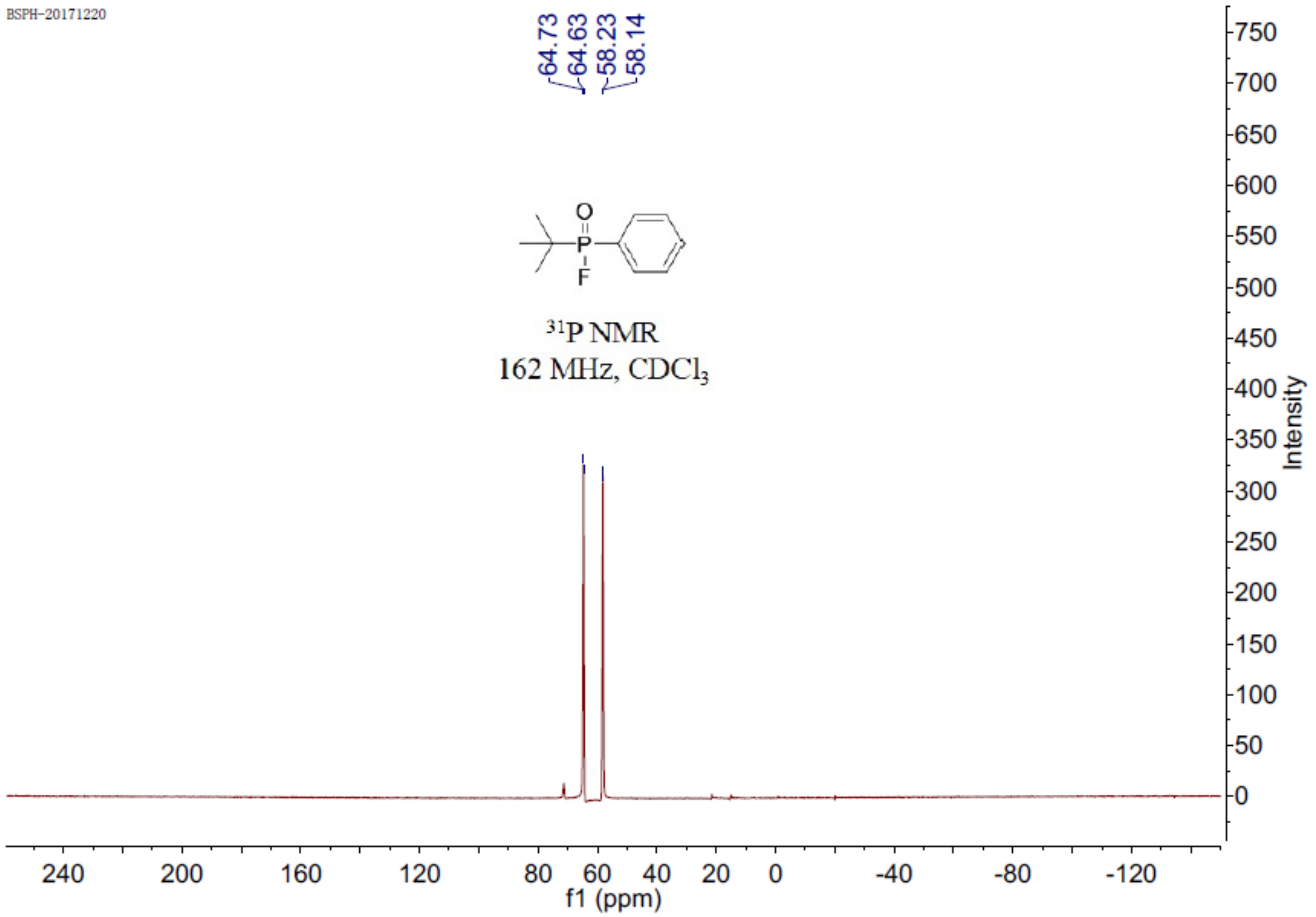

Supplementary Figure 39. ^{31}P NMR spectrum of **3**.

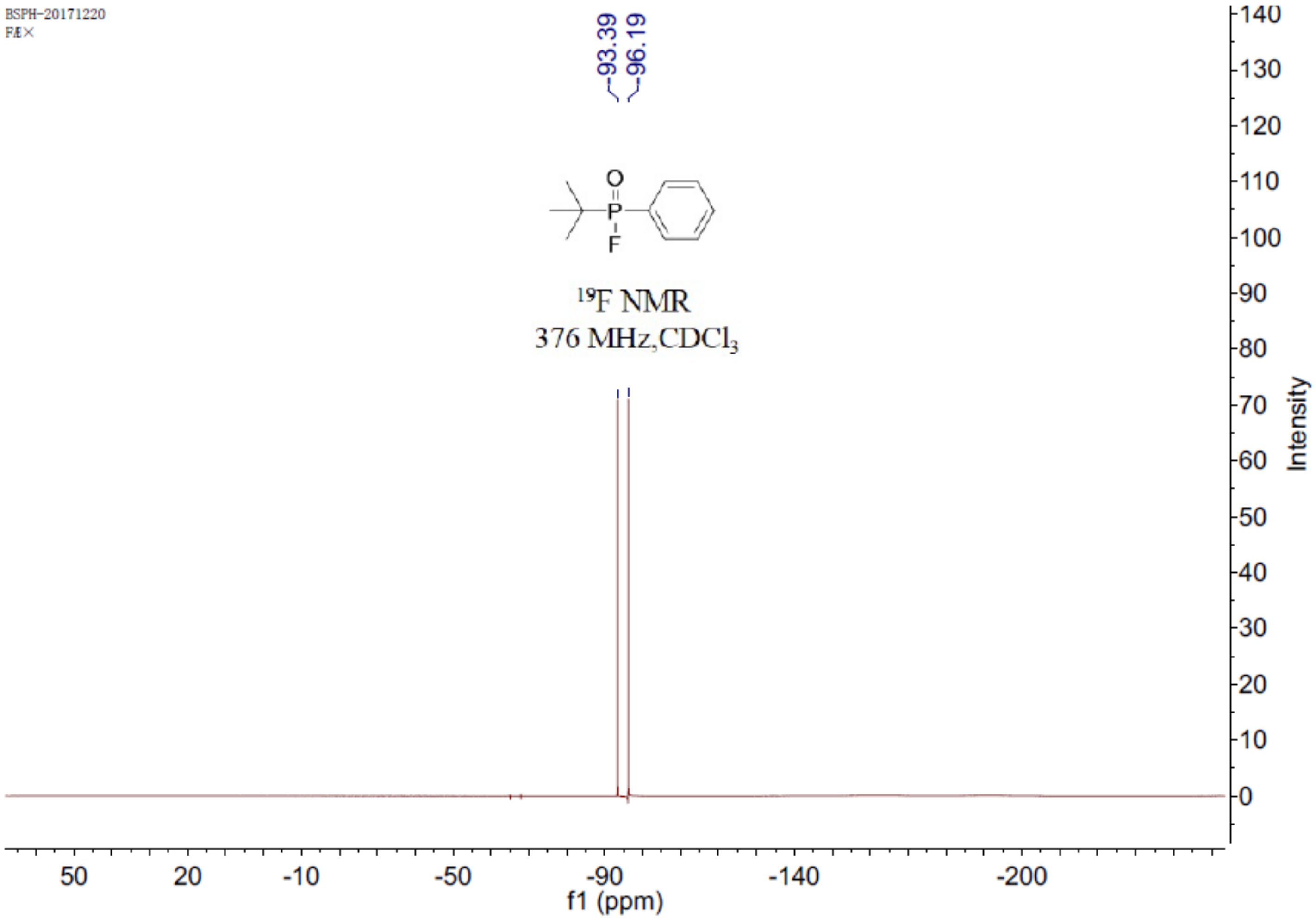

Supplementary Figure 40. ^{19}F NMR spectrum of **3**.

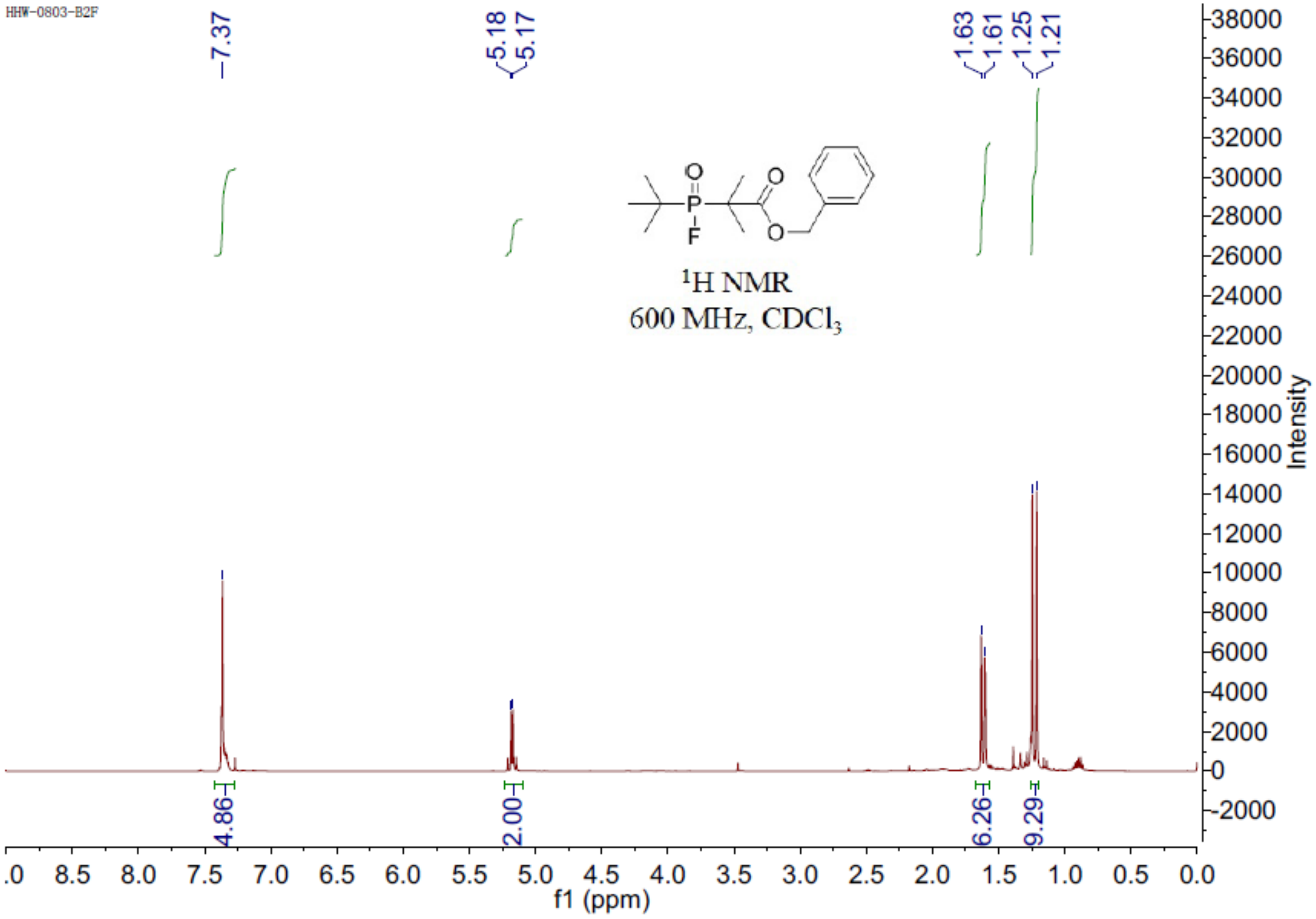

Supplementary Figure 41. ^{1}H NMR spectrum of **4**.

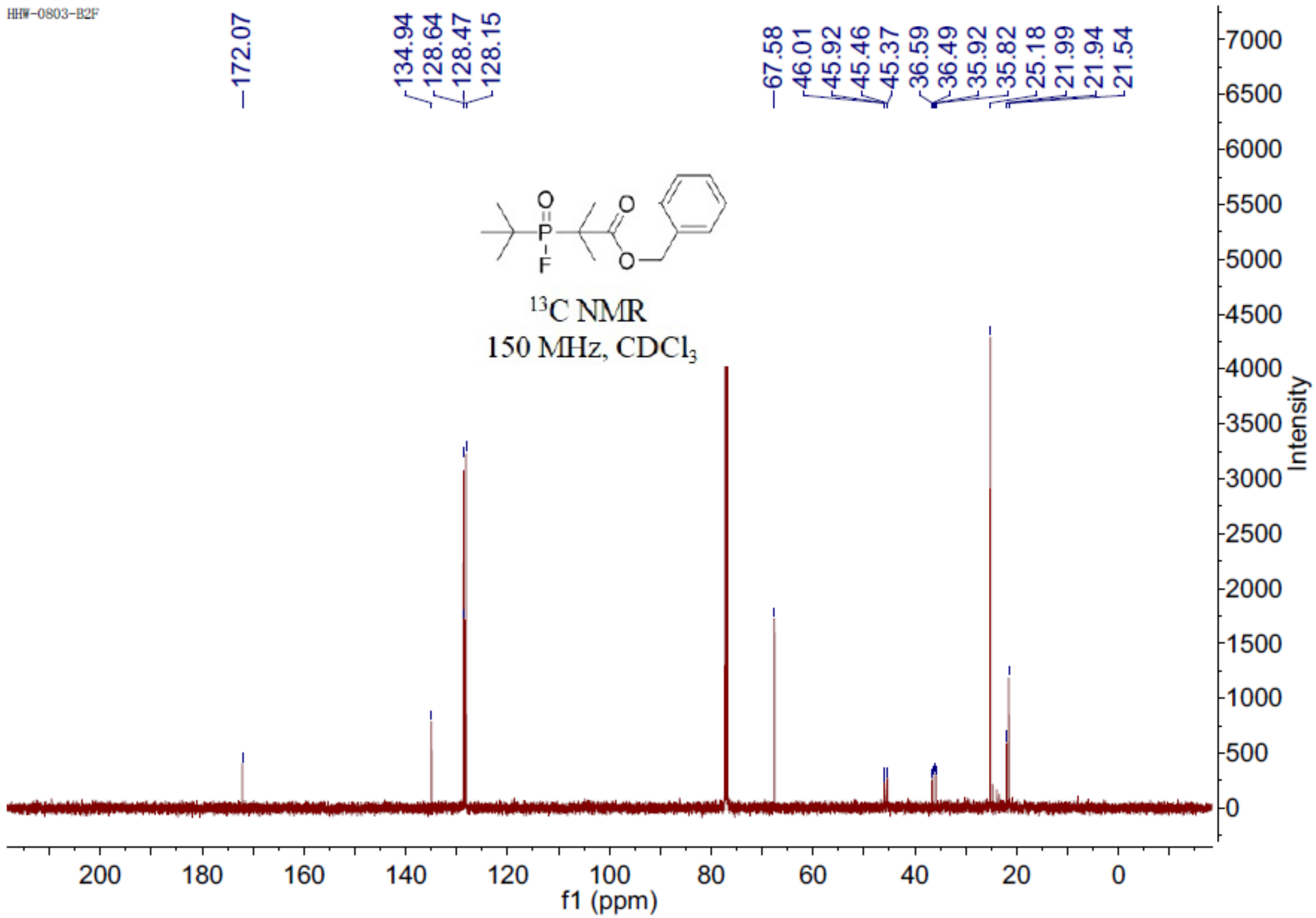

Supplementary Figure 42. ^{13}C NMR spectrum of **4**.

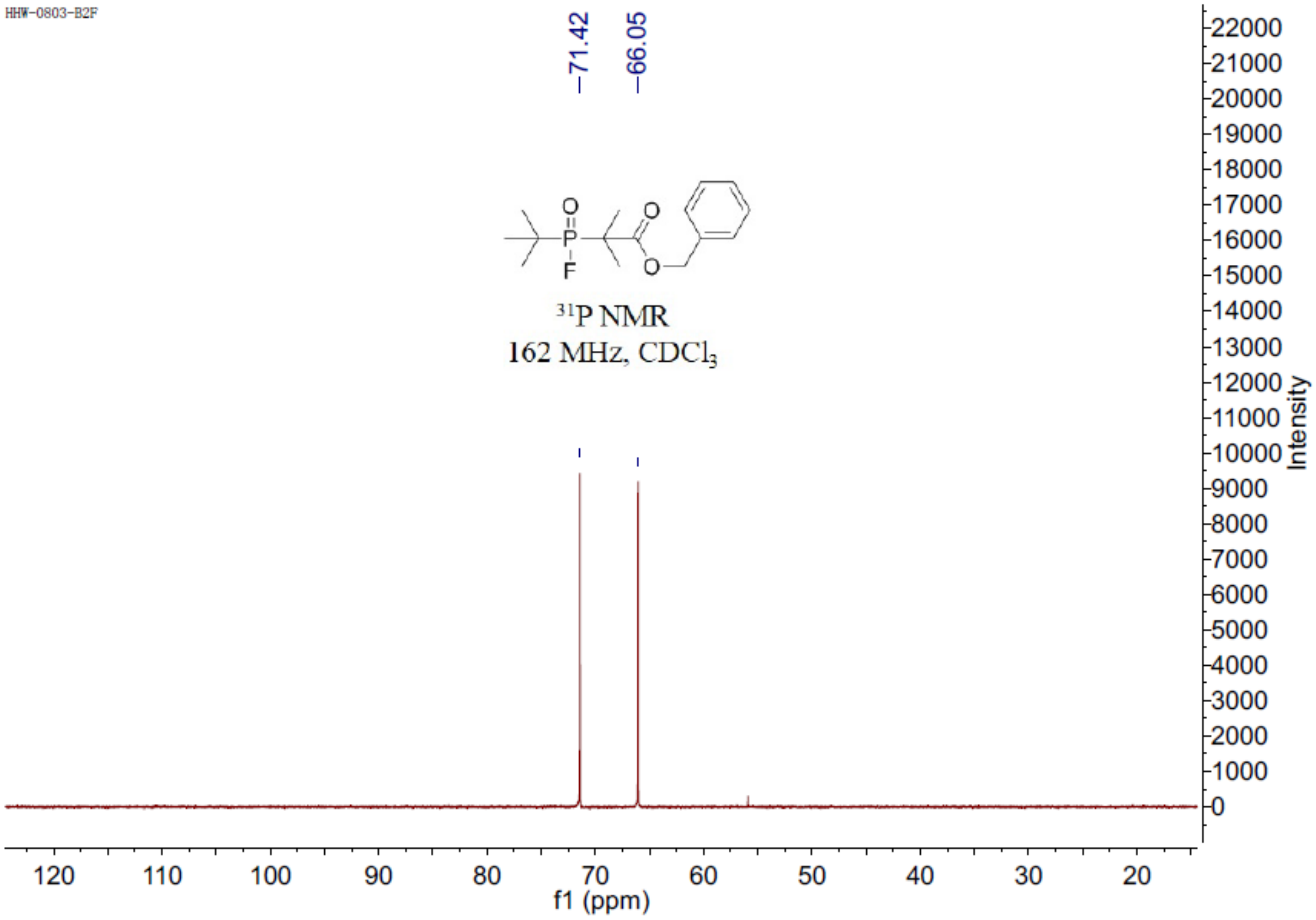

Supplementary Figure 43. ^{31}P NMR spectrum of **4**.

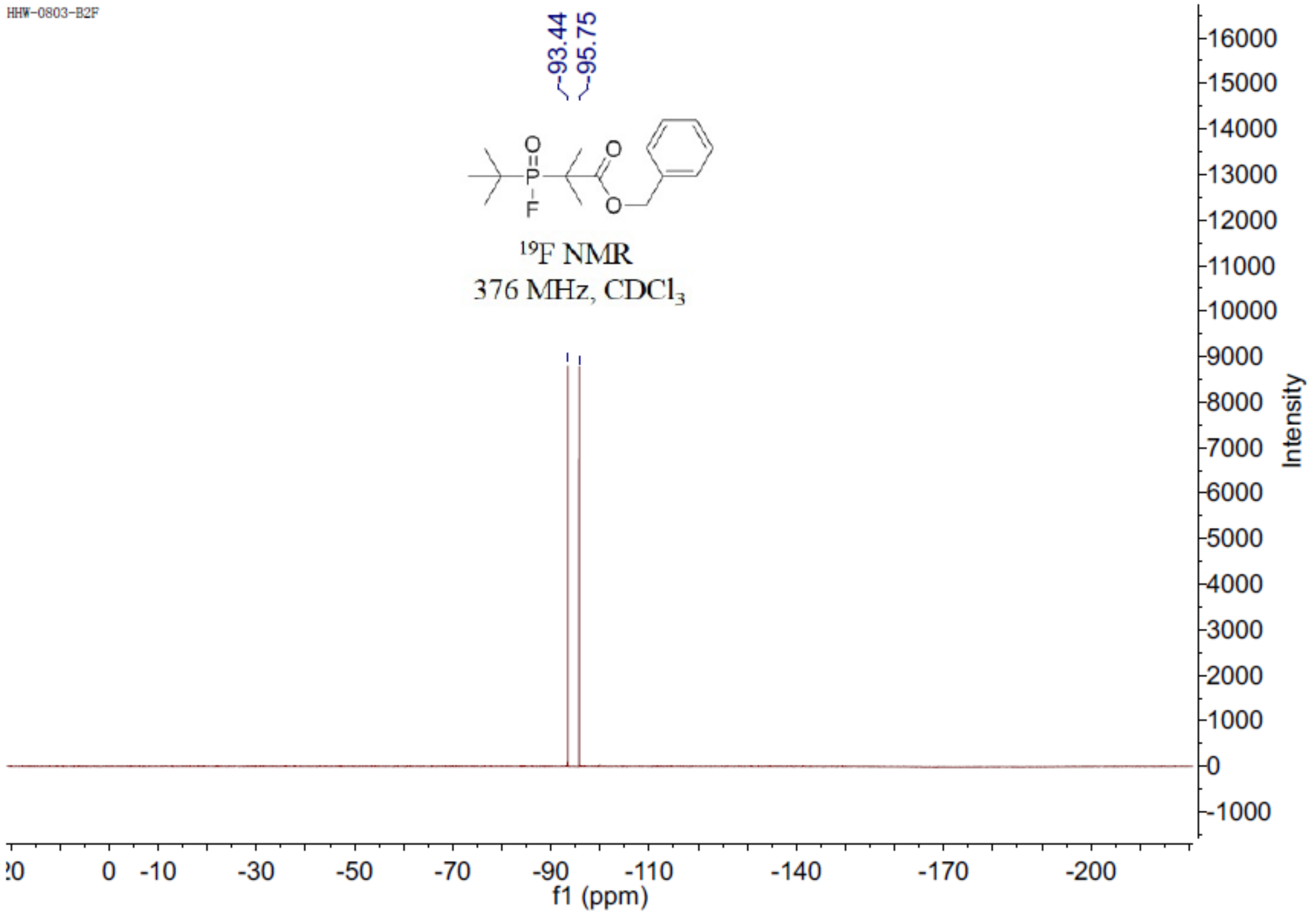

Supplementary Figure 44. ^{19}F NMR spectrum of **4**.

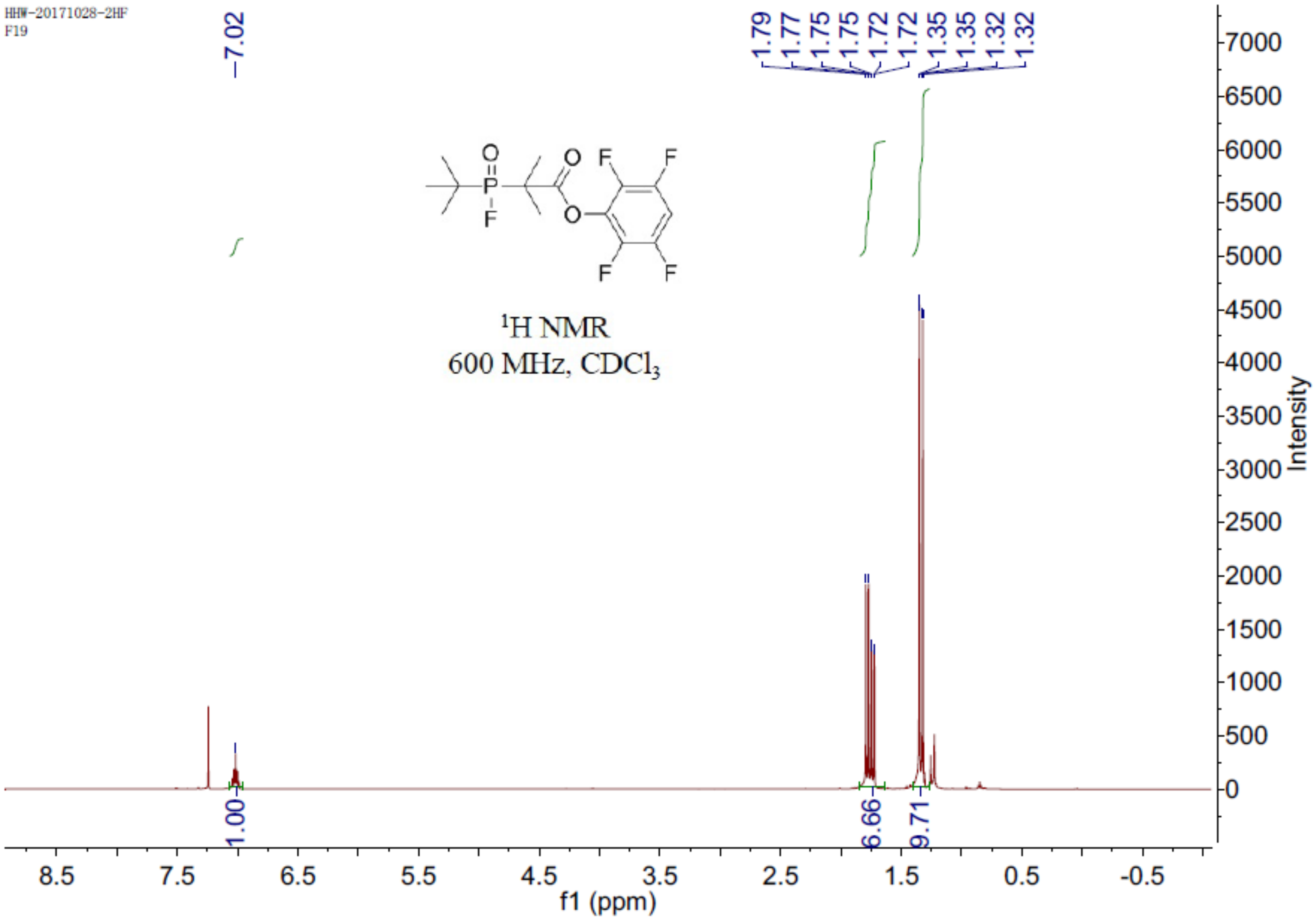

Supplementary Figure 45. ^{1}H NMR spectrum of **5**.

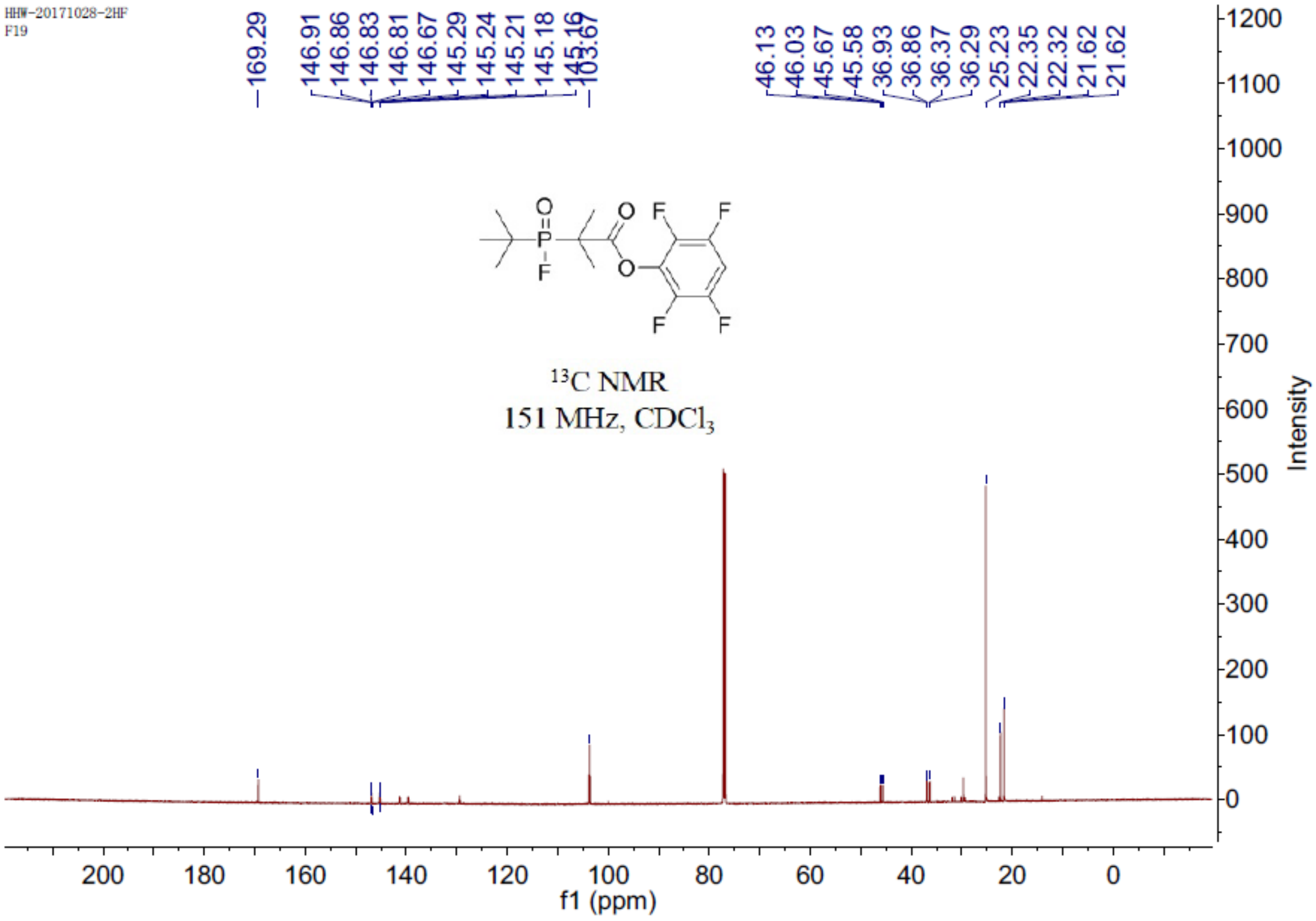

Supplementary Figure 46. ^{13}C NMR spectrum of **5**.

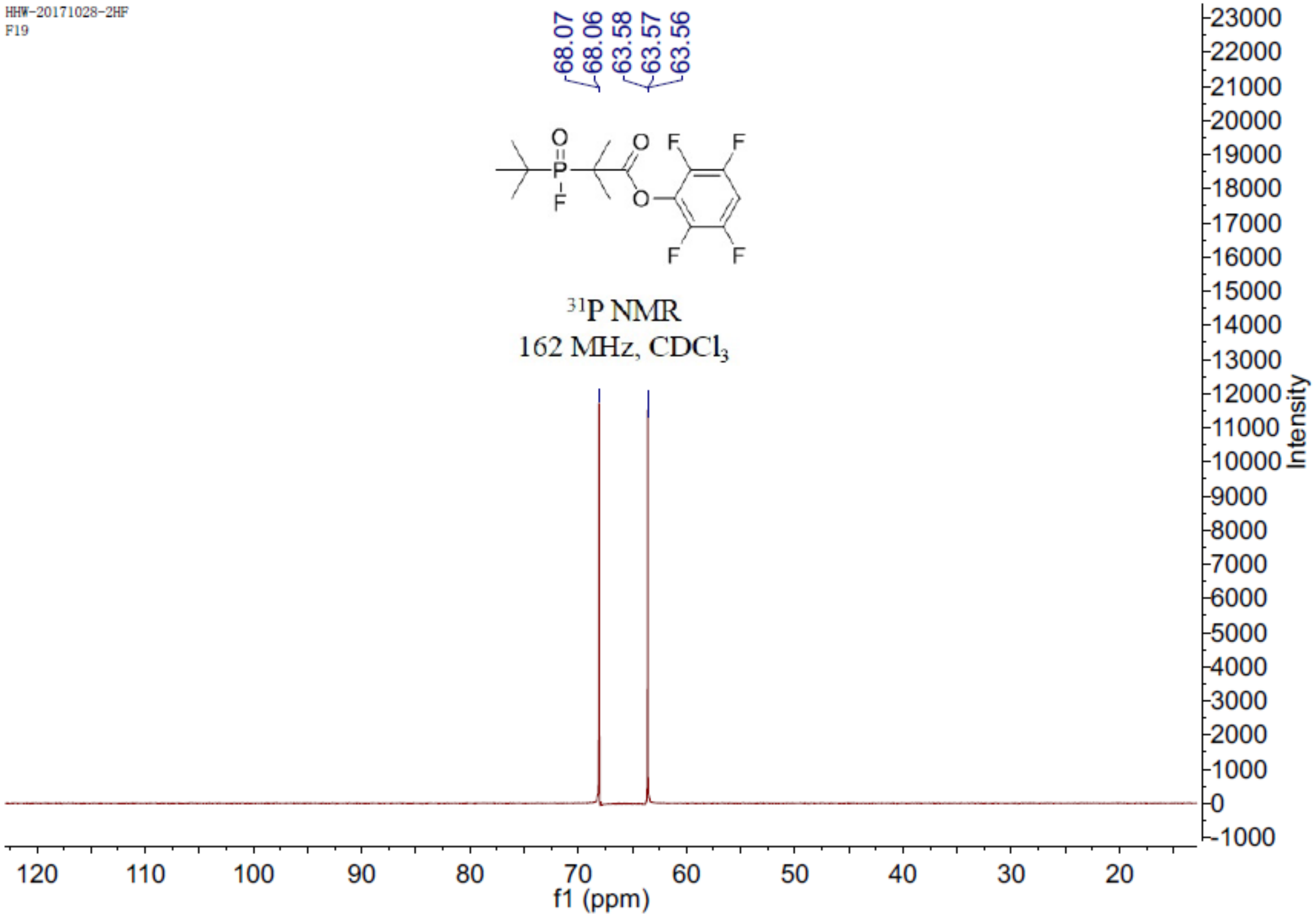

Supplementary Figure 47. ^{31}P NMR spectrum of **5**.

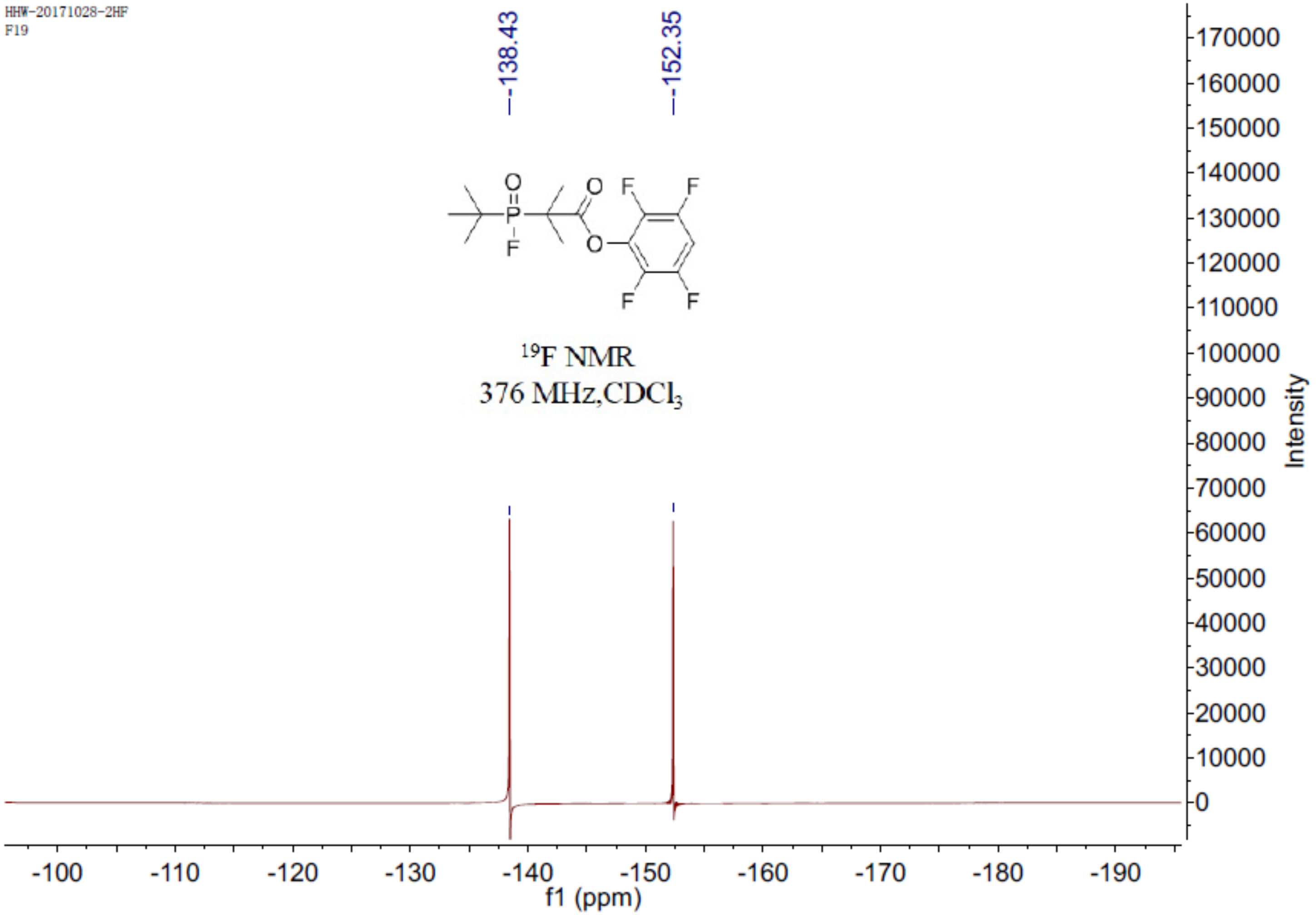

Supplementary Figure 48. ^{19}F NMR spectrum of **5**.

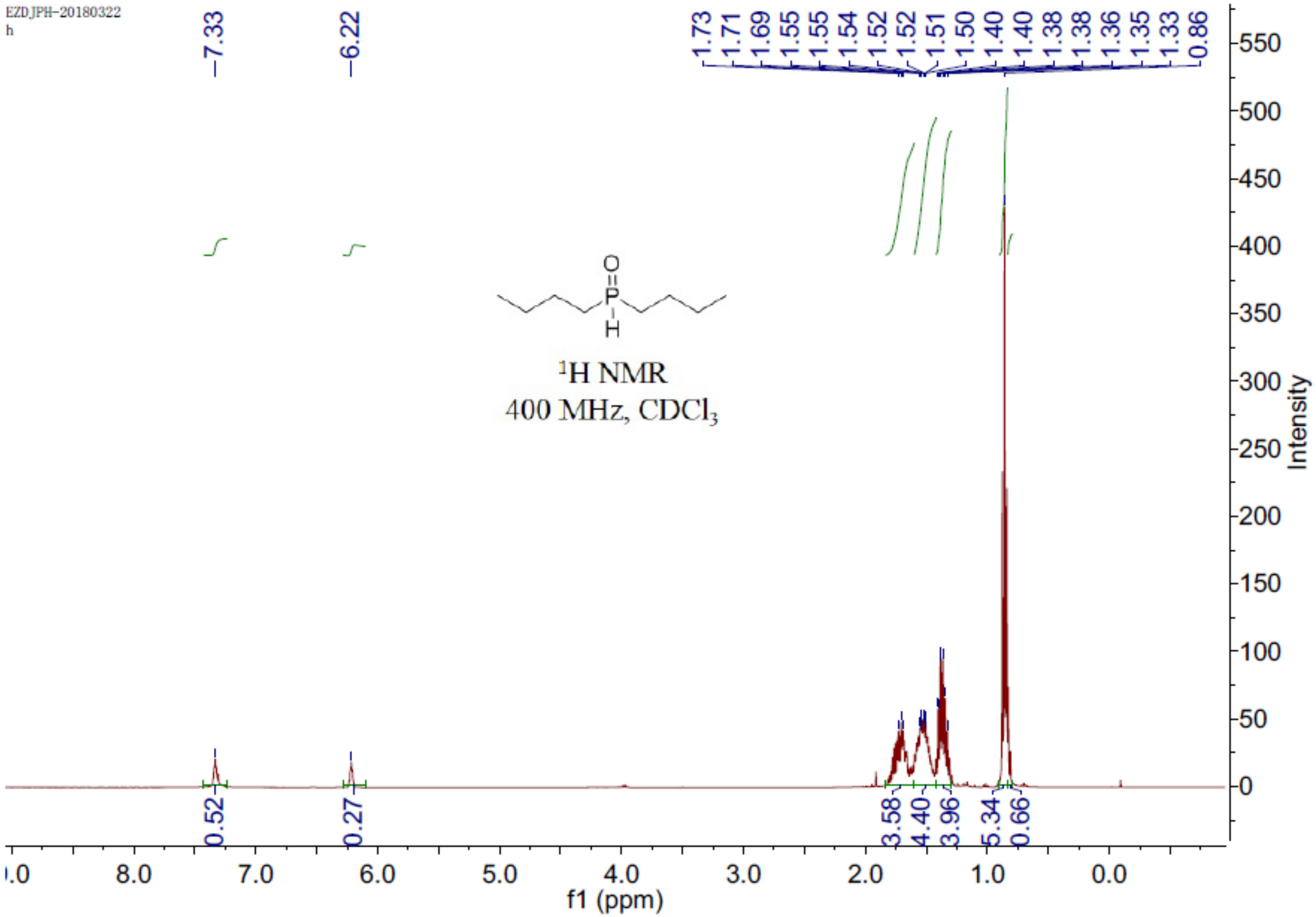

Supplementary Figure 49. ^{1}H NMR spectrum of **6**.

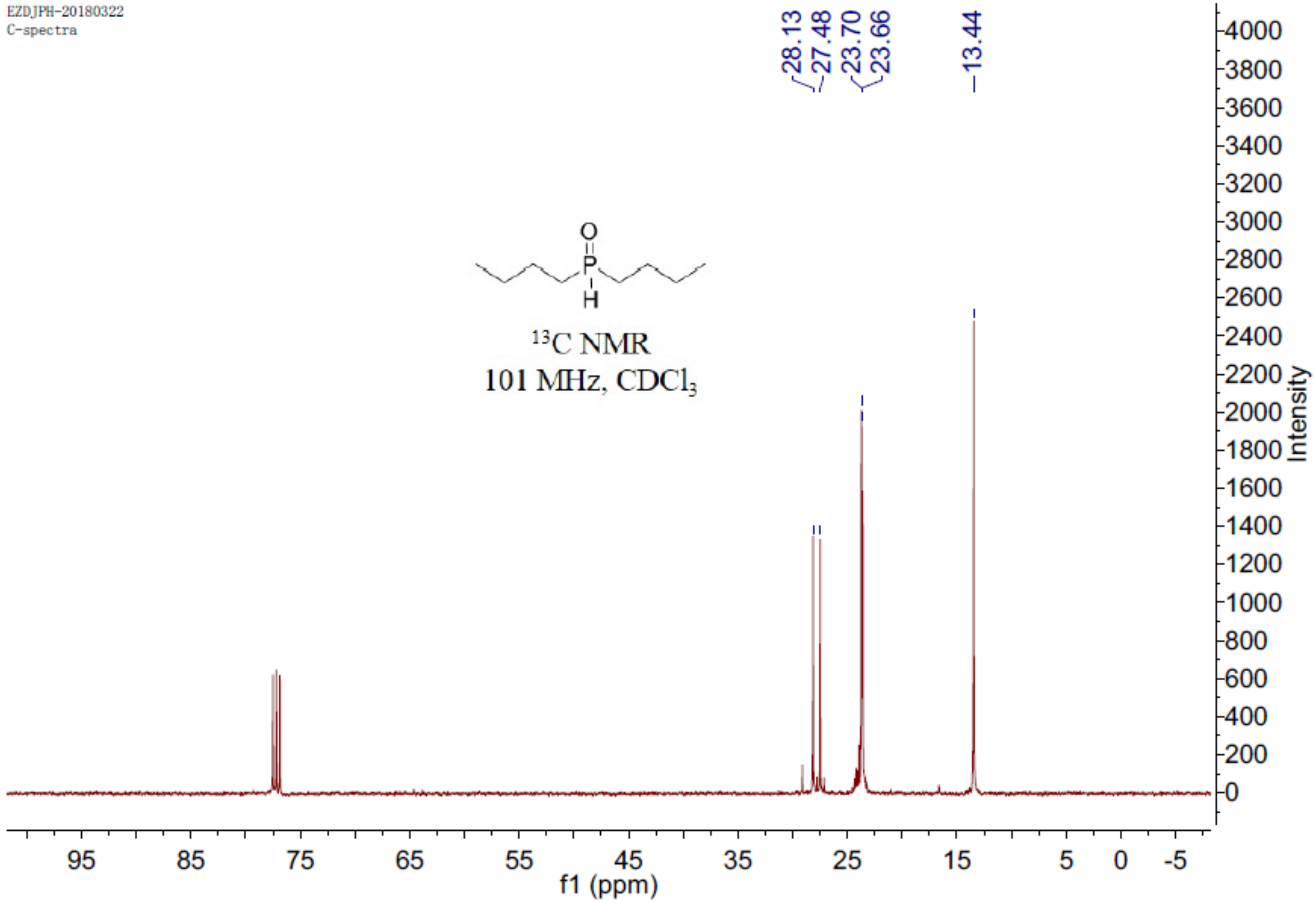

Supplementary Figure 50. ^{13}C NMR spectrum of **6**.

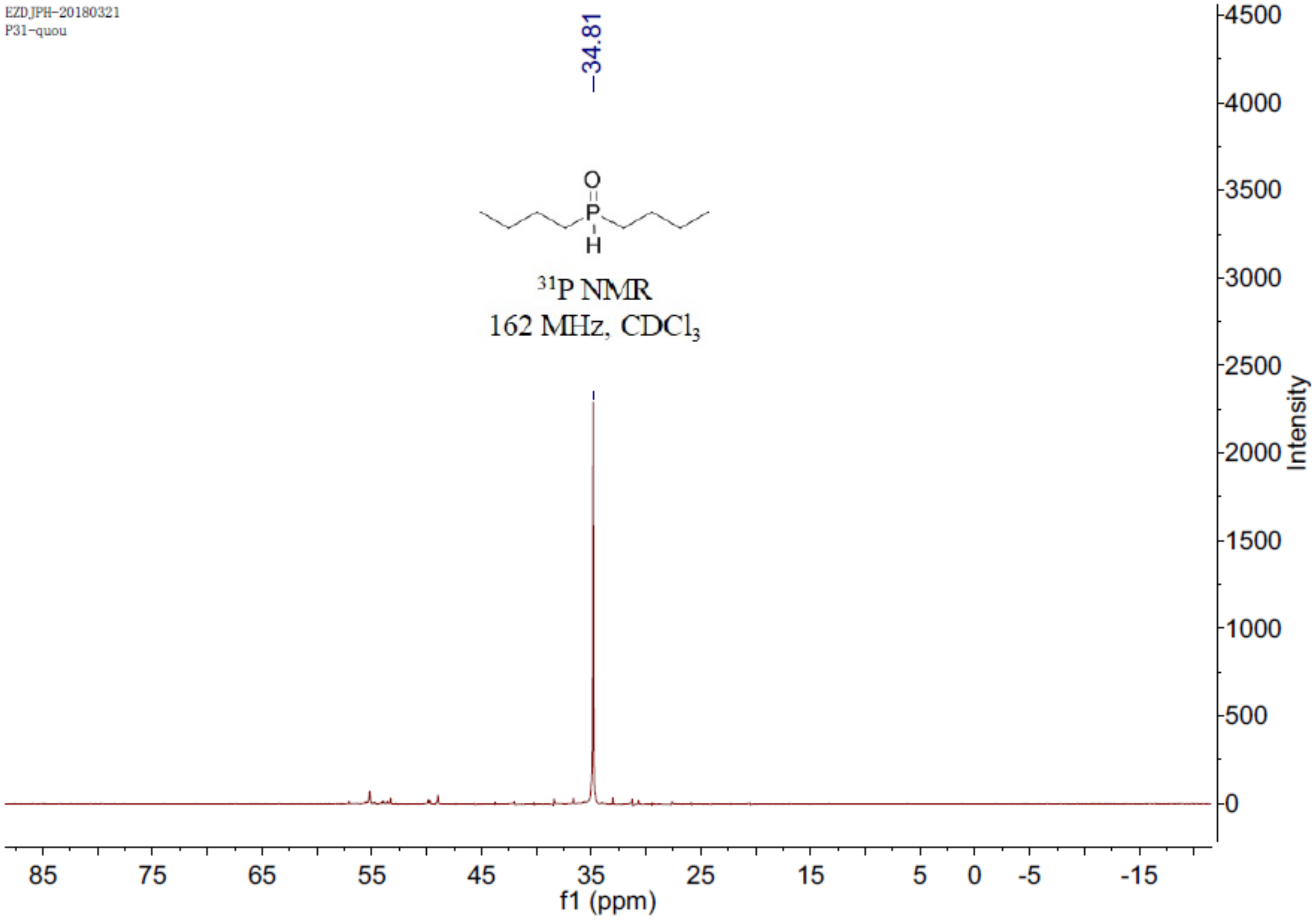

Supplementary Figure 51. ^{31}P NMR spectrum of **6**.

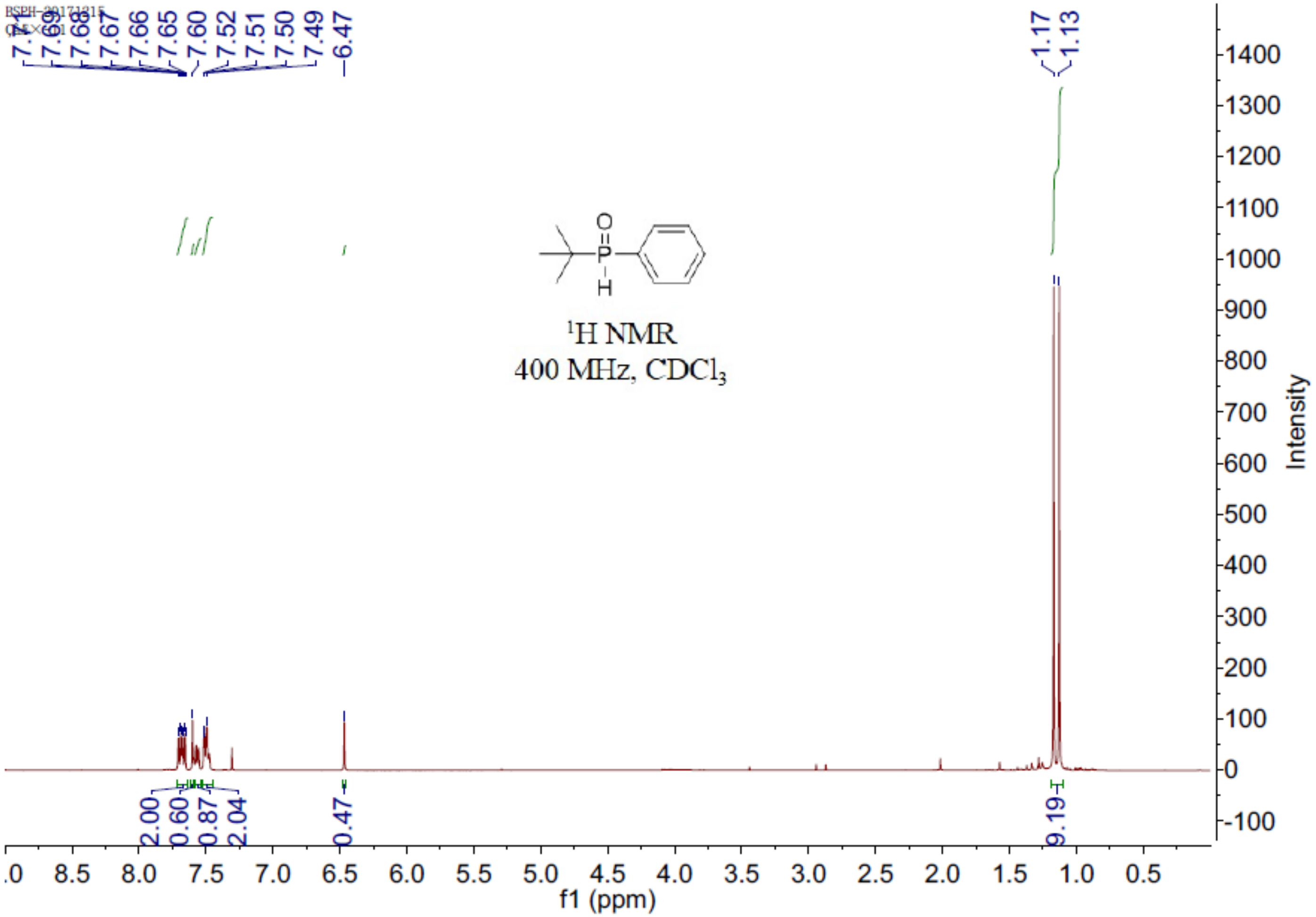

Supplementary Figure 52. ^{1}H NMR spectrum of **7**.

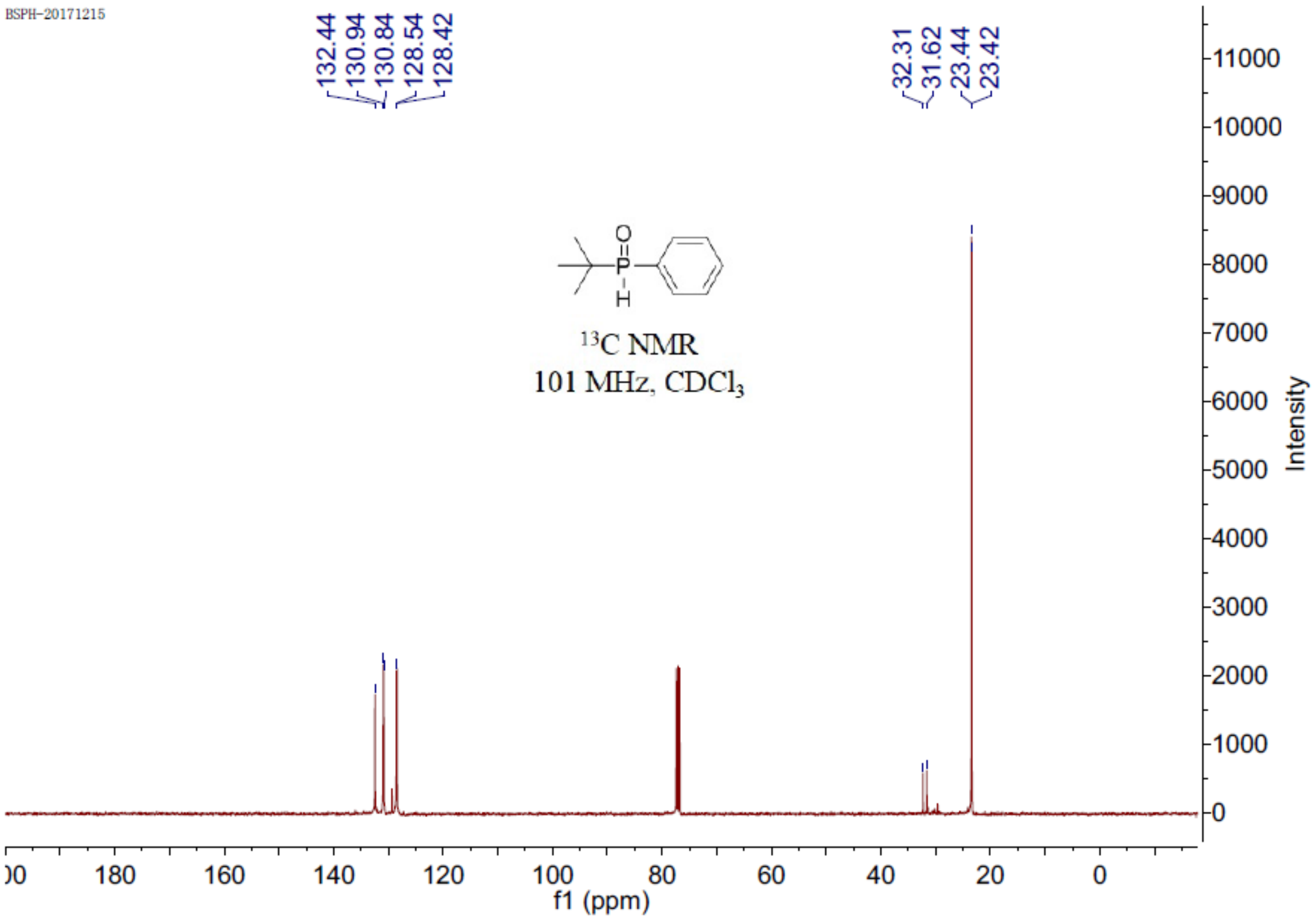

Supplementary Figure 53. ^{13}C NMR spectrum of **7**.

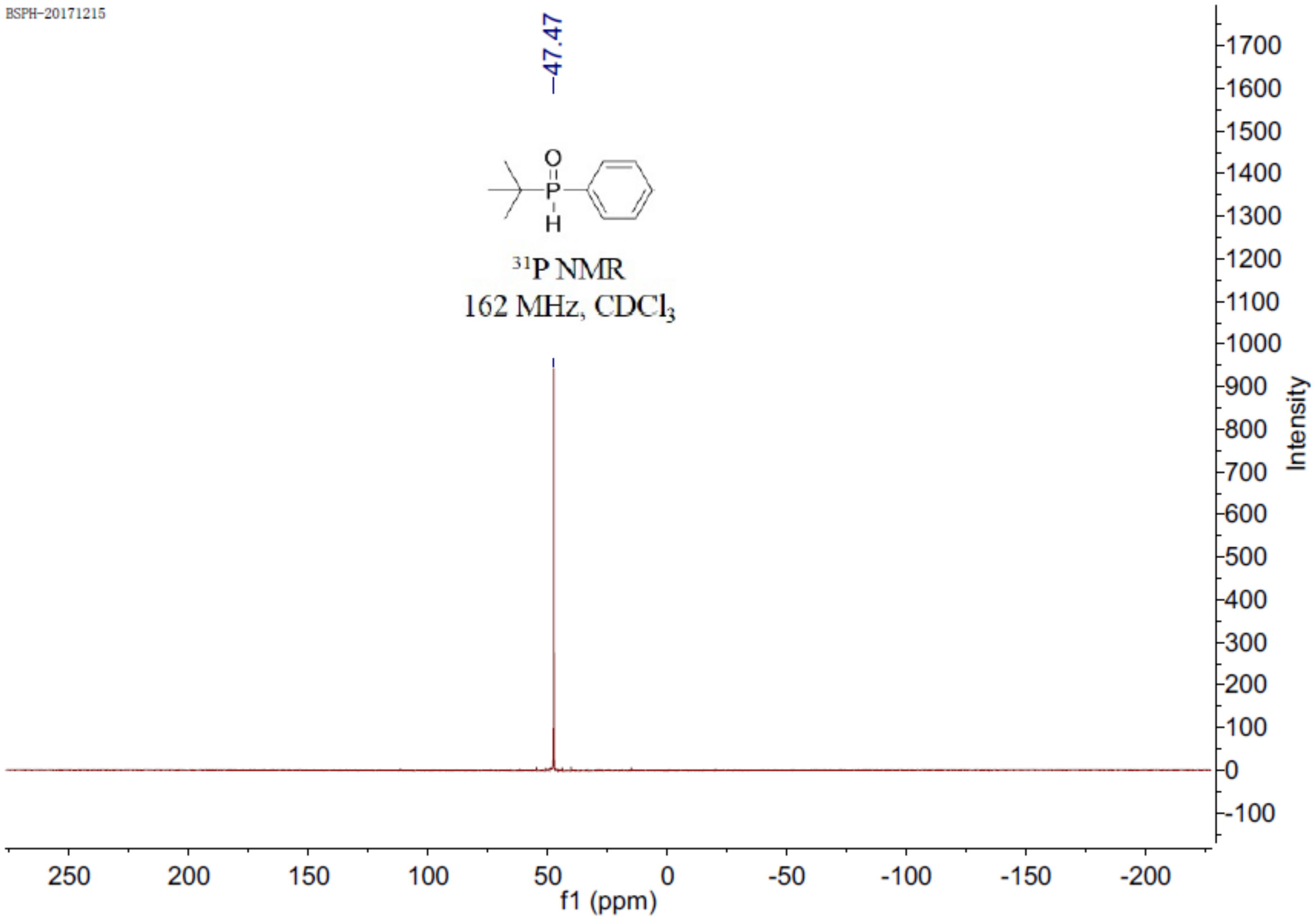

Supplementary Figure 54. ^{31}P NMR spectrum of **7**.

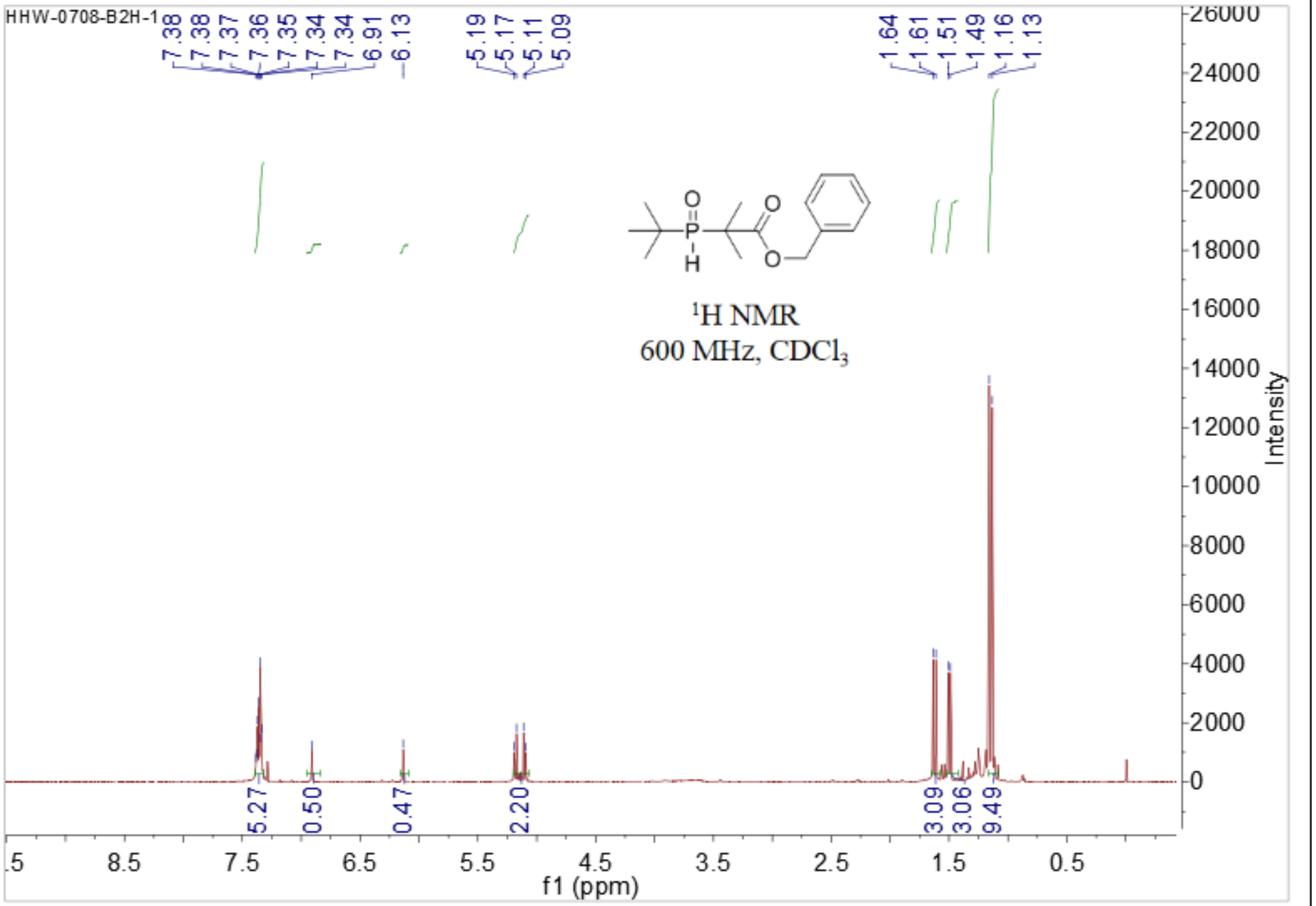

Supplementary Figure 55. ^{1}H NMR spectrum of **8**.

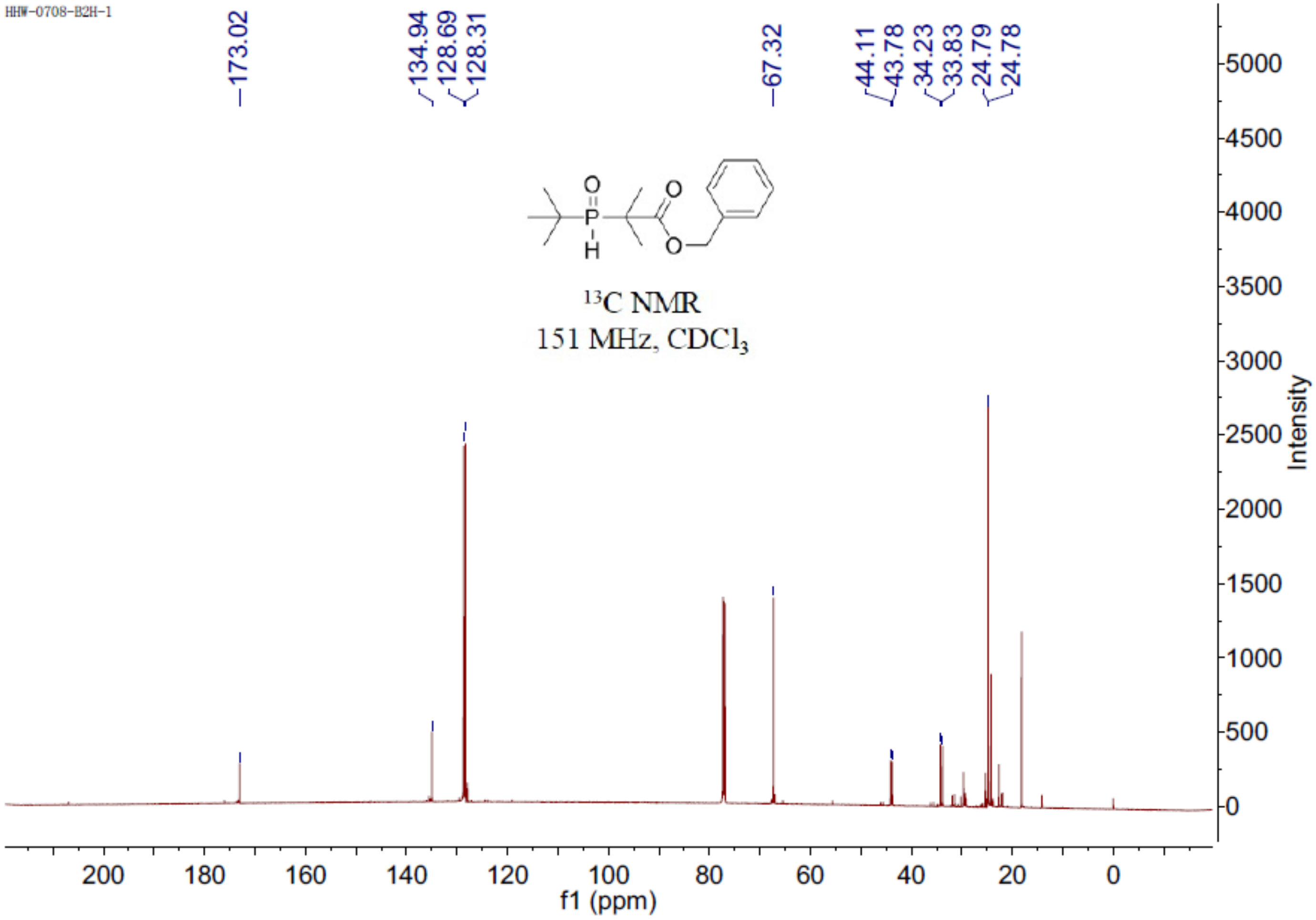

Supplementary Figure 56. ^{13}C NMR spectrum of **8**.

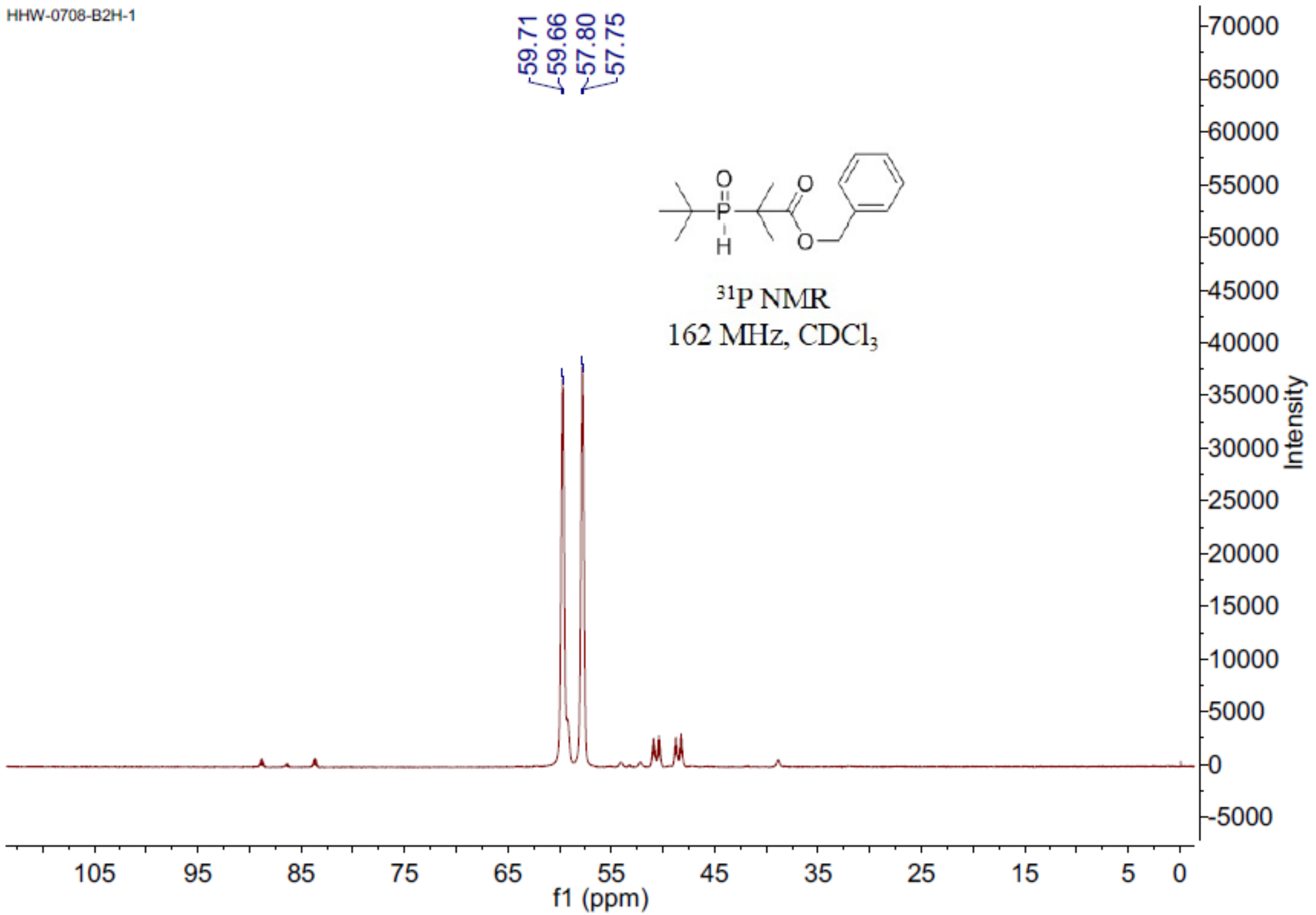

Supplementary Figure 57. ^{31}P NMR spectrum of **8**.

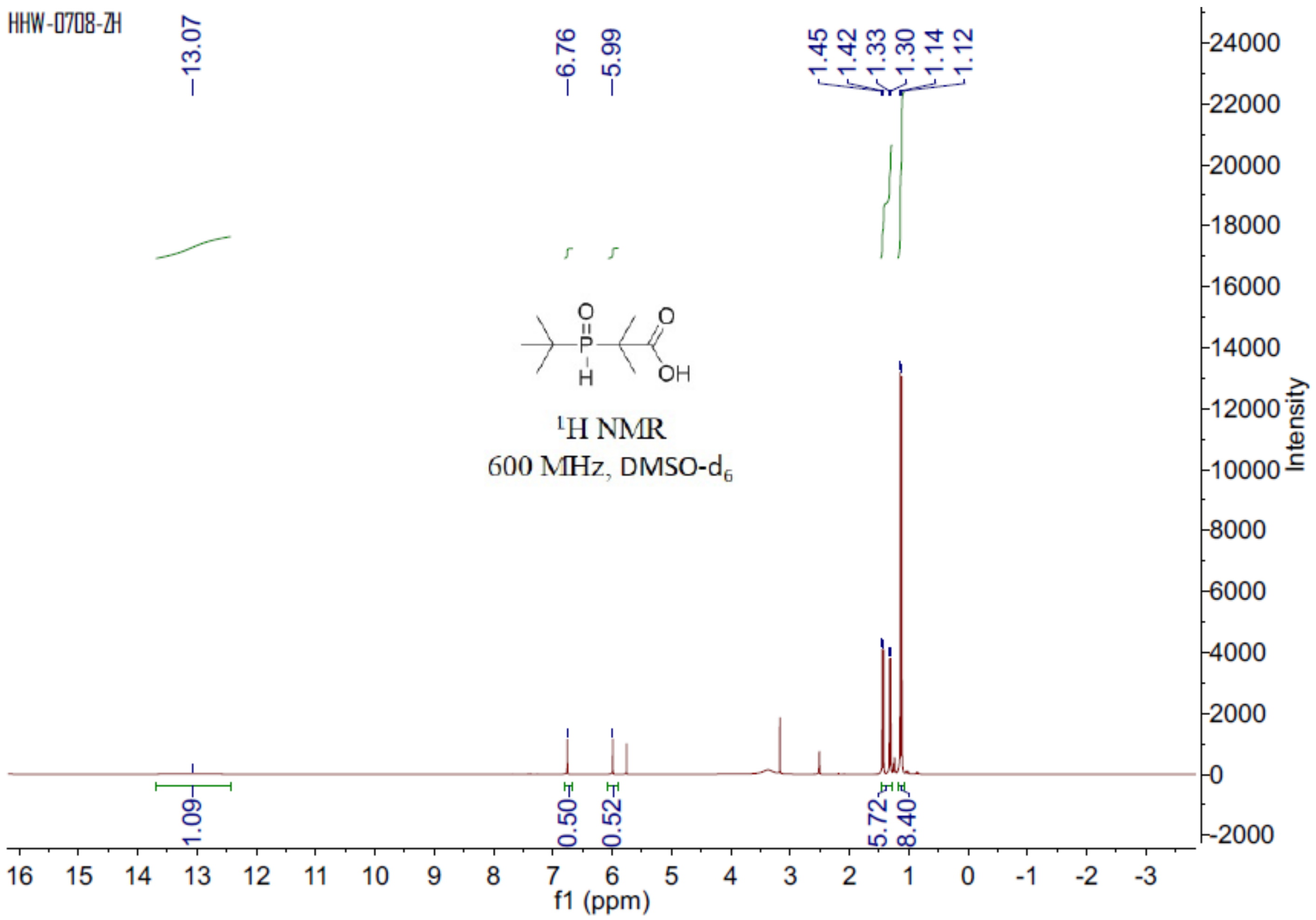

Supplementary Figure 58. ^{1}H NMR spectrum of **9**.

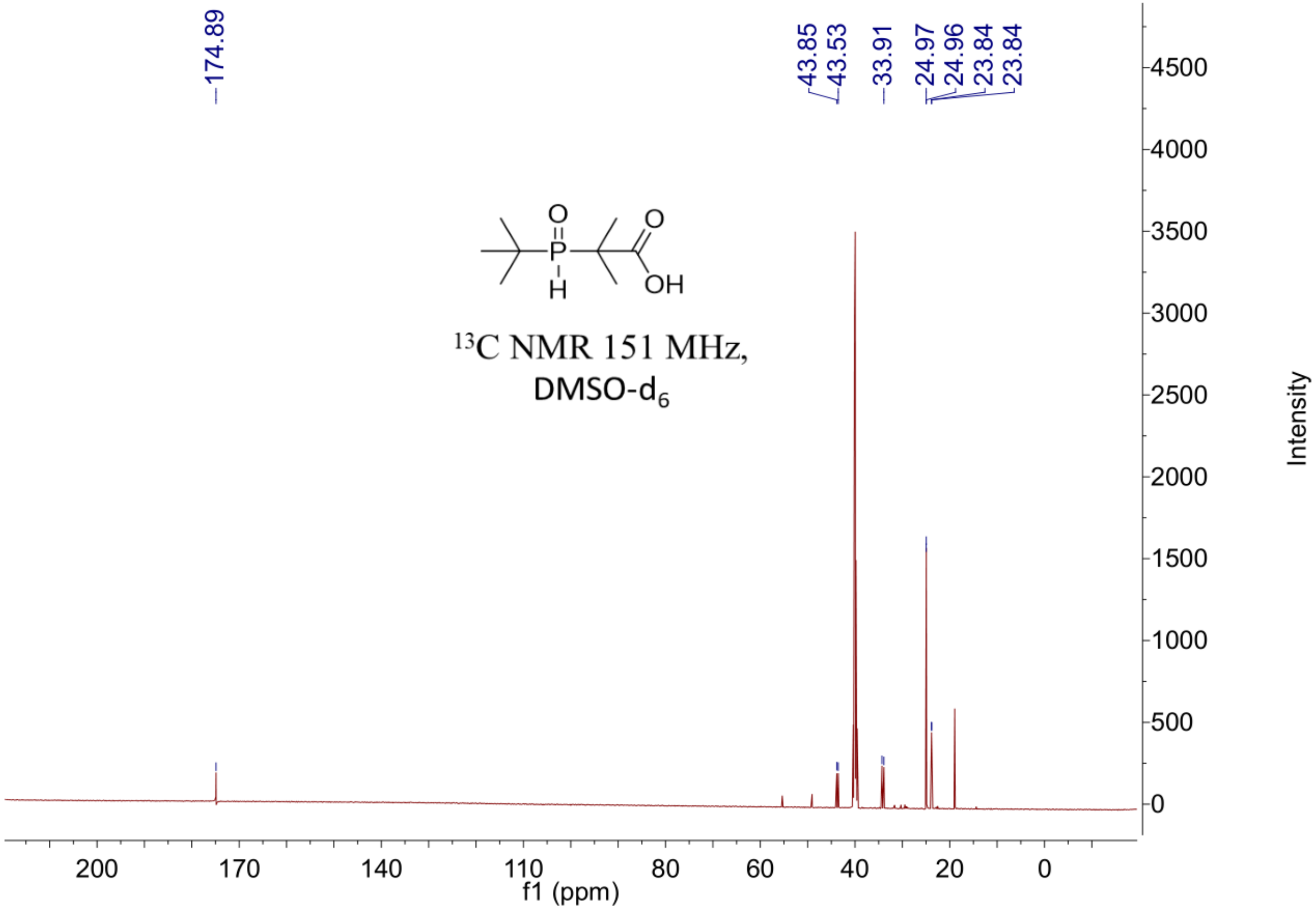

Supplementary Figure 59. ^{13}C NMR spectrum of **9**.

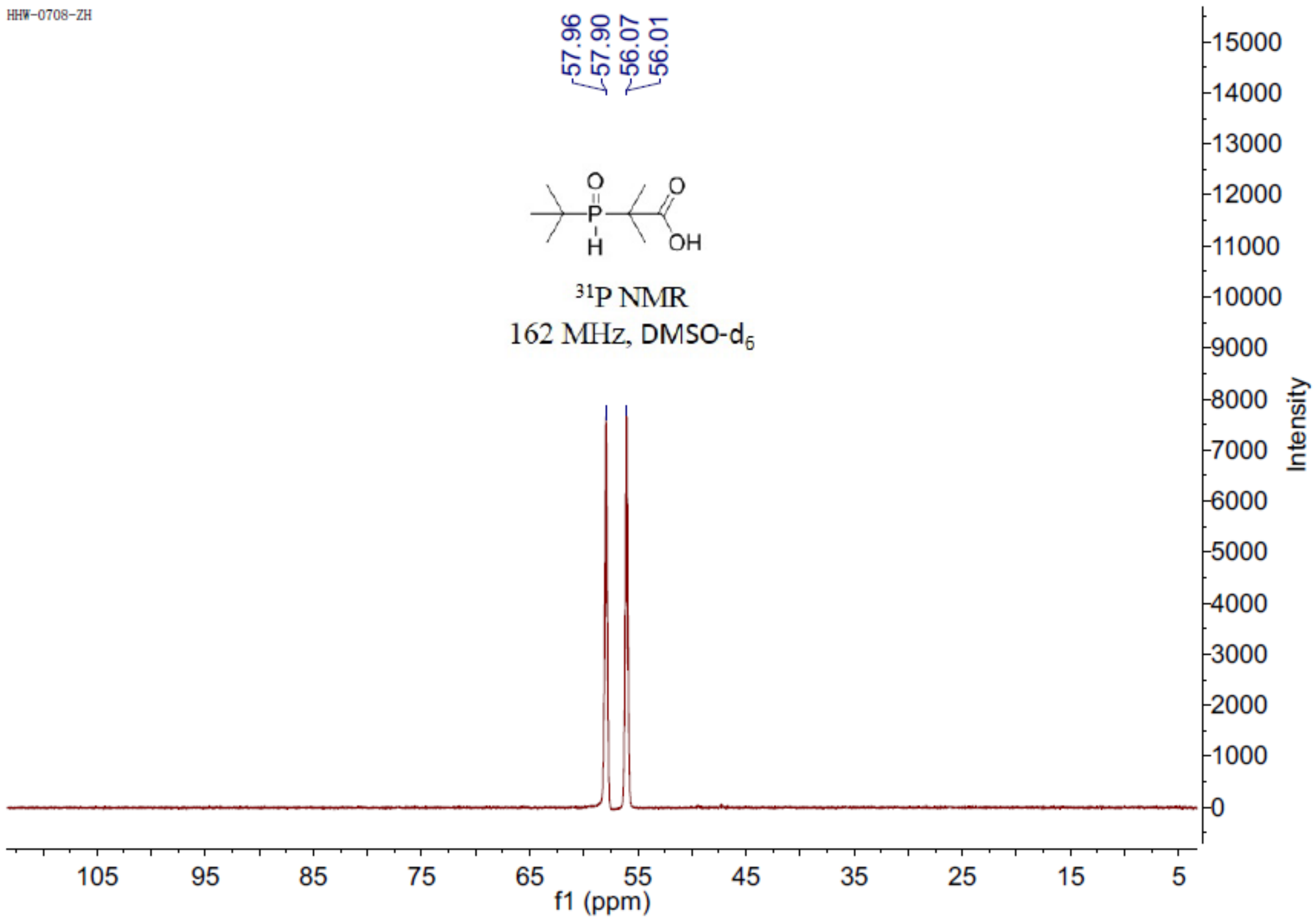

Supplementary Figure 60. ^{31}P NMR spectrum of **9**.

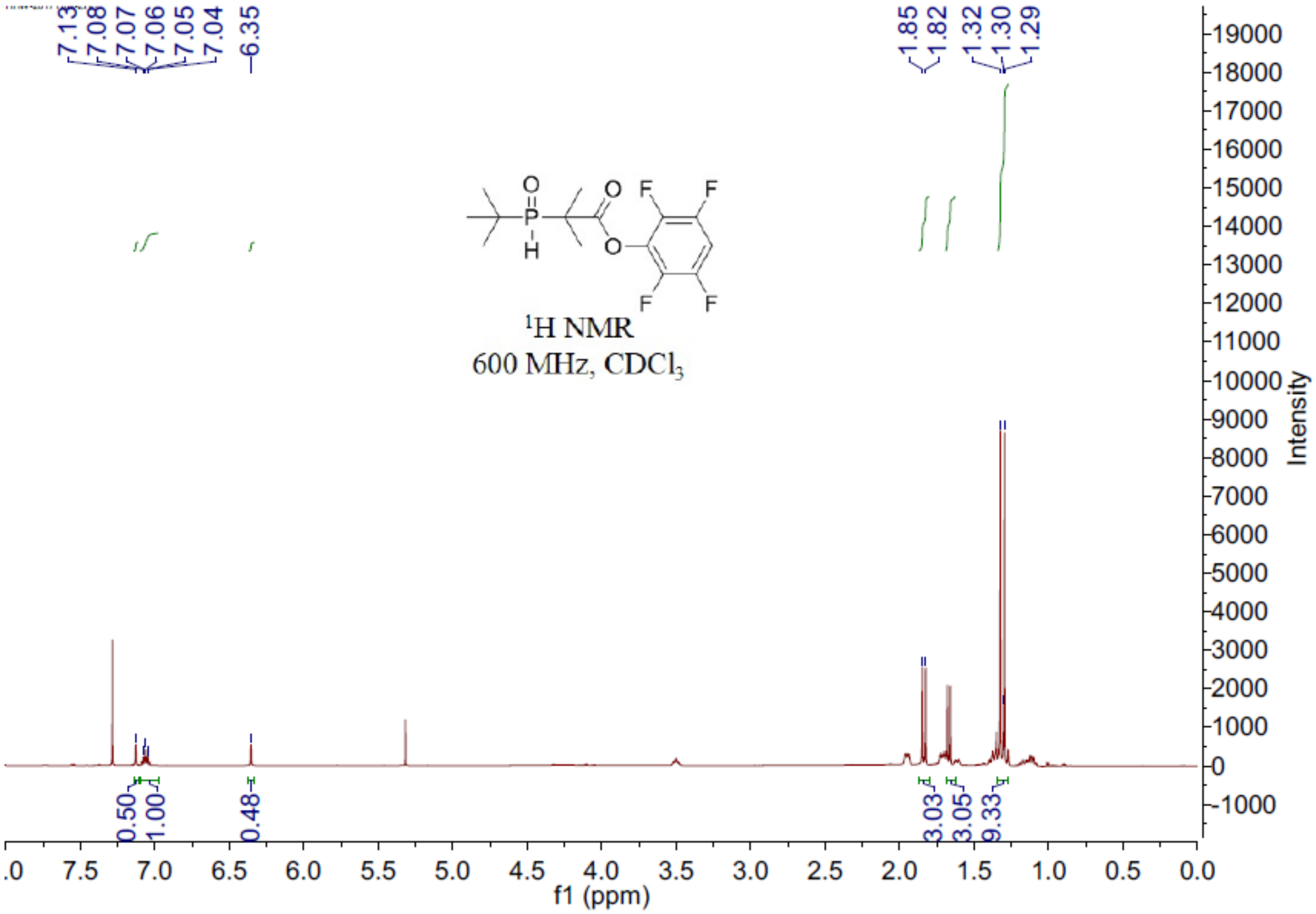

Supplementary Figure 61. ^{1}H NMR spectrum of **10**.

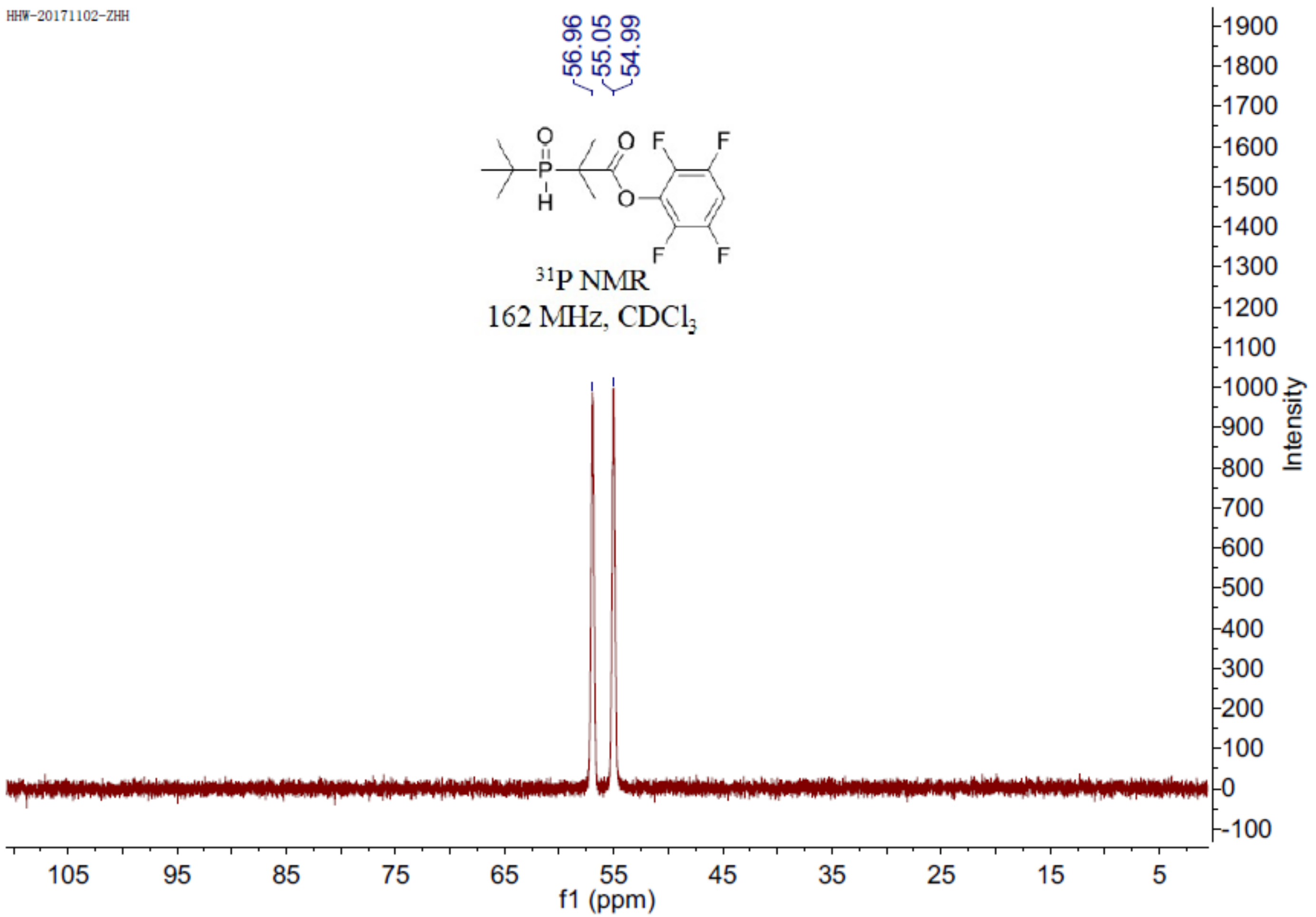

Supplementary Figure 62. ^{31}P NMR spectrum of **10**.

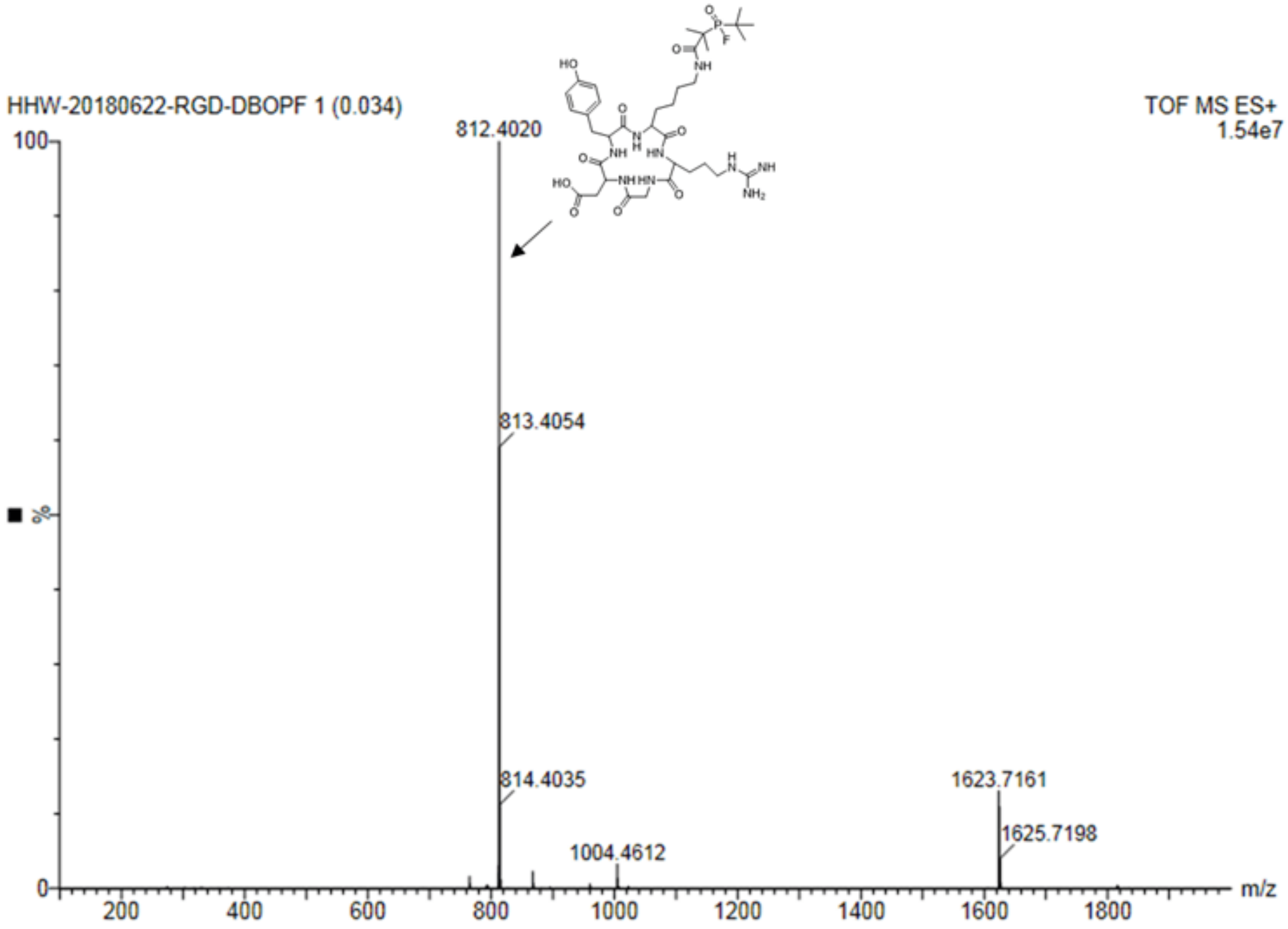

Supplementary Figure 63. Mass spectrum of DBPOF-c(RGDyk).

Supplementary Tables

Supplementary Table 1. RCYs of selected flourophosphine fluoride acceptors. (n = 3)

Precursor	Product	Method	RCY (%)
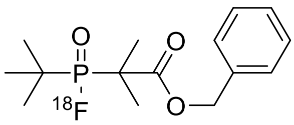 **4**	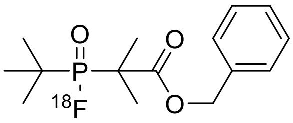 $[^{18}F]$**4**	I	50 ± 5
		II	60 ± 6
		III	80 ± 8
		IV	>97
		V	93 ± 6
		VI	>97
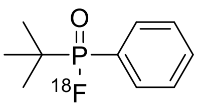 **3**	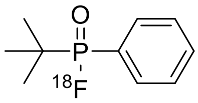 $[^{18}F]$**3**	II	93 ± 3
		IV	89 ± 4
		VI	92 ± 5
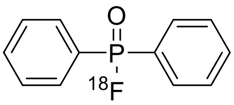 **2**	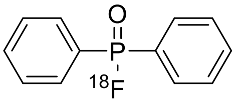 $[^{18}F]$**2**	II	0
		IV	5 ± 4
		VI	27 ± 4

Supplementary Table 2. Summary of radiosynthesis of ^{18}F-DBPOF-c(RGDyk).

Entry	Activity at EOB (mCi)	Precursor (μmol)	Radiolabeled product (mCi, EOS)	Molar activity (Ci/μmol, EOS)	RCY[a]
Automated radiosynthesis	600-1200	1-3	90-300	0.06-0.13	15~25%
Manual radiosynthesis	30-50	1-3	9-15	0.006-0.01	>30%

EOB = end of bombardment, EOS = end of synthesis. [a] The amount of activity in the final product expressed as the percentage of starting activity.

Supplementary Methods

General information

All the reagents we used in the syntheses were commercially available without further purification. The synthesis reactions were carefully monitored by thin layer chromatography (TLC) on TLC Silica gel 60 F254 aluminum sheets (Merck, Germany), since all compounds were visualized when being illuminated with a short wavelength UV lamp (λ = 254 nm). Column chromatography purifications were performed on silica gel (54 - 74 μm, Qingdao Haiyang Chemical Co., Ltd., China).

^{1}H-NMR, ^{13}C-NMR, ^{31}P-NMR and ^{19}F-NMR spectra were obtained at a Bruker NMR spectrometer (Avance II 600 MHz) or a ZhongKeNiuJin NMR spectrometer (AS 400 MHz) with samples dissolved in either $CDCl_3$ or DMSO-d_6 solutions at room temperature. Tetramethylsilane (TMS) was used as the internal standard. All the chemical shifts were reported as δ values relative to the internal TMS. The coupling constants were reported in Hertz (Hz). Multiplicity was defined by s (singlet), d (doublet), t (triplet), and m (multiplet). Mass spectrometry was acquired from a Xevo G2-XS Tof instrument (Waters, USA).

Radiochemical purity (RCY) was evaluated by radio-HPLC analysis on a Thermo Fisher Dionex Ulti-Mate 3000 equipped with a SPD-20A UV detector (λ = 254 nm) as well as a Bioscan Flow Count 3200 NaI/PMT γ-radiation scintillation detector. HPLC separations were achieved on an Ultimate XB-C18 (5 μm, 10 mm × 250 mm, Welch, China) or a Xtimate SEC-300 column (Welch, China). HPLC analyses were achieved on an Ultimate XB-C18 (5 μm, 4.6 mm × 250 mm, Welch) or a Xtimate SEC-300 column (Welch, China) with the Thermo HPLC. The purities of the synthesized standard compounds were determined using analytical HPLC and were found to be higher than 98%. Residual solvent analysis was acquired by Gas chromatography (GC) from a 6890N Network GC System (Agilent, USA). The centrifuge we used in Log *D* determination was a Centrifuge 5424 R (Eppendorf, Germany).

Synthesis of dibutylphosphinic fluoride (1)

0.10 mol **6** was added into a tube charged with stirred suspension of $CuCl_2$ (0.20 mol) and CsF (0.20 mol) in acetone (30 mL) at room temperature in one shot[3]. The resulting mixture was stirred at room temperature for 2 h and monitored by ^{31}P NMR. The crude product was purified by column chromatography on silica gel to give the desired product **1** as an oily liquid (yield 50%). ^{1}H NMR (400 MHz, $CDCl_3$) δ (ppm): 0.89-0.97 (m, 6H); 1.25-1.28 (m, 8H); 1.44-1.49 (m, 4H). ^{19}F NMR (376 MHz, $CDCl_3$) δ (ppm): -76.81~-79.52 (d, 1F, J = 1029.66 Hz). ^{31}P NMR (162 MHz, $CDCl_3$) δ (ppm): 68.95-75.38 (d, 1P, J = 1088.18 Hz).

[I]How $CuCl_2$ and CsF give the phosphonyl fluoride follows Putative reaction mechanism. In the first step, hydrogen of dialkylphosphites was substituted by chlorine of CuCl in an electrophilic displacement reaction with $CuCl_2$[Cu(II)] converted to CuCl[Cu(I)]. In the second step, the chloride of dialkyl chlorophosphate was nucleophilically displaced by fluoride with CsF converted to CsCl.

Synthesis of diphenylphosphinic fluoride (2)

0.10 mol diphenylphosphine oxide was added into a tube charged with stirred suspension of $CuCl_2$ (0.20 mol) and CsF (0.20 mol) in acetone (30 mL) at room temperature in one shot[3]. The resulting mixture was stirred at room temperature for 2 h and monitored by ^{31}P NMR. The crude product was purified by column chromatography on silica gel to give the desired product **2** as a white solid (yield 60%). ^{1}H NMR (400 MHz, $CDCl_3$) δ (ppm): 7.87- 7.84 (m, 4H), 1.73-1.55 (m, 4H), 7.66 - 7.63 (m, J_1 = 7.39 Hz, J_2 = 7.48 Hz, 2H), 7.56-7.52, (m, 4H). ^{31}P NMR (162 MHz, $CDCl_3$) δ (ppm): 40.77. ^{19}F NMR (376 MHz, $CDCl_3$) δ (ppm): -69.51, -72.22 (d, 1F, J = 1019.28 Hz).

Synthesis of *tert*-butyl(phenyl)phosphinic fluoride (3)

7 (0.10 mol) was added to a tube with stirred suspension of $CuCl_2$ (0.20 mol) and CsF (0.20 mol) in acetone (30 mL) at room temperature in one shot[3]. The resulting mixture was stirred at room temperature for 2 h and monitored by MS and ^{31}P NMR. The crude product was purified by flash chromatography on silica gel to give the desired

product **3** as a white solid (yield 60%). ^{1}H NMR (400 MHz, $CDCl_3$) δ (ppm): 1.09 (d, 9H, J = 16.70 Hz); 7.37-7.41 (m, 2H); 7.48-7.52 (m, 1H); 7.64-7.69 (m, 2H). ^{13}C NMR (101 MHz, $CDCl_3$) δ (ppm): 133.16-133.15 (d, ^{1}J = 2.22 Hz), 132.40-132.37, 132.30-132.28 (dd, ^{2}J = 2.48 Hz, ^{3}J = 2.92 Hz), 128.51-128.39 (d, ^{4}J = 13.04 Hz), 33.43-33.27, 32.47-32.32 (dd, ^{5}J = 15.61 Hz, ^{6}J = 15.19 Hz), 23.71(s).^{31}P NMR (162 MHz, $CDCl_3$) δ (ppm): 61.27. ^{19}F NMR (376 MHz, $CDCl_3$) δ (ppm): -94.84.

Synthesis of benzyl 2-(*tert*-butylfluorophosphoryl)-2-methylpropanoate (4)

0.10 mol **8** was added to a tube with stirred suspension of $CuCl_2$ (0.20 mol) and CsF (0.20 mol) in acetone (30 mL) at room temperature in one shot[3]. The resulting mixture was stirred at room temperature for 2 h and monitored by MS and ^{31}P NMR. The crude product was purified by column chromatography on silica gel to give the desired product **4** as a white solid (yield 50%). ^{1}H NMR (600 MHz, $CDCl_3$) δ (ppm): 1.22 (d, 9H, J = 16.64 Hz); 1.61 (d, 6H, J = 14.76 Hz); 5.17 (d, 2H, J = 6.61 Hz); 7.36 (M, 5H). ^{13}C NMR (151 MHz, $CDCl_3$) δ (ppm): 172.07(s), 134.94(s), 128.64-128.15 128.69-128.31 (t, ^{1}J = 20.04 Hz, ^{2}J = 42.23 Hz), 67.58 (s), 46.01-45.92 (d, ^{3}J= 12.83 Hz), 45.46-45.37 (d, ^{4}J= 12.45 Hz), 36.66-36.49 (t, ^{5}J = 8.76 Hz, ^{6}J = 11.90 Hz), 35.92-35.82 (d, ^{7}J = 12.27 Hz), 25.18 (s), 21.99-21.94 (d, ^{8}J = 5.44Hz), 21.54 (s). ^{19}F NMR (564 MHz, $CDCl_3$) δ (ppm): -94.59 (d, 1F, J = 10087.82 Hz). ^{31}P NMR (243 MHz, $CDCl_3$) δ (ppm): 68.72 (d, 1P, J = 350.70).

Synthesis of 2,3,5,6-tetrafluorophenyl 2-(*tert*-butylfluorophosphoryl)-2-methylpropanoate (5)

0.10 mol **10** was added into a tube with stirred suspension of $CuCl_2$ (0.20 mol) and CsF (0.20 mol) in acetone (30 mL) at room temperature in one shot[3]. The resulting mixture was stirred at room temperature for 2 h and monitored by MS and ^{31}P NMR. The crude product was purified by column chromatography on silica gel to using dichloromethane and methanol (5:1) to give the desired products **5** as a white solid (yield 70%) ^{1}H NMR (600 MHz, $CDCl_3$) δ (ppm): 1.13 (d, 9H, J = 17.11 Hz); 1.79 (d, 6H, J_1 = 14.55 Hz, J_2 = 13.28 Hz); 7.01 (m, 1H). ^{13}C NMR (151 MHz, $CDCl_3$) δ

169.29 (s), 146.91-146.67 (m), 145.29-145.13 (m), 103.67 (t, ${}^{1}J$ = 23.63 Hz, ${}^{2}J$ = 23.63 Hz), 46.13-46.03 (d, ${}^{3}J$ = 13.89 Hz), 45.67-45.58 (d, ${}^{4}J$ = 13.43 Hz), 36.93-36.86 (d, ${}^{5}J$ = 11.37 Hz), 36.37-36.29 (d, ${}^{6}J$ = 11.37 Hz), 25.23 (s), 22.35-22.32 (d, ${}^{7}J$ = 4.74 Hz), 21.62-21.62 (d, ${}^{8}J$C = 1.07 Hz). ^{31}P NMR (243 MHz, $CDCl_3$) δ (ppm): 68.07-63.56 (d, J = 1111.31 Hz). ^{19}F NMR (564 MHz, $CDCl_3$) δ (ppm): -138.43--152.35 (d, J = 7811.65 Hz).

Synthesis of dibutylphosphine oxide (6)

To a solution of n-butyllithium (12 mmol of n-butyllithium, 3.0 equiv.) in tetrahydrofuran cooled to -80 °C, diethyl phosphonate (4 mmol, 1.0 equiv.) was added dropwise under at -80 °C. The reaction was allowed to be stirred at room temperature for 5 h[4]. The reaction mixture was quenched with 1 M HCl and extracted with ethyl acetate. The combined organic layers were washed with brine, dried over anhydrous Na_2SO_4, filtered, concentrated and purified with silica gel column chromatography (petroleum ether/ethyl acetate) to afford **6** as an oily liquid (yield 65%) ^{1}H NMR (400 MHz, $CDCl_3$) δ (ppm): 7.33-6.22 (d, J= 442.2 Hz, 1H), 1.73-1.55 (m, 4H), 1.54-1.50 (m, 4H), 1.40-1.33, (m, J = 14.6, 7.1 Hz, 4H), 0.86 (t, J = 7.1 Hz, 6H). ^{13}C NMR (101 MHz, $CDCl_3$) δ (ppm): 28.13-27.48 (d, ${}^{1}J$ = 64.8 Hz), 23.70 (s), 23.66 (s), 13.44 (s). ^{31}P NMR (162 MHz, $CDCl_3$) δ (ppm): 34.81.

Synthesis of *tert*-butyl(phenyl)phosphine oxide (7)

7 was prepared by slowly adding *tert*-butyllithium (0.1 mol) to a suspension of dichlorophenylphosphine (0.1 mol) at -80 °C. The mixture was allowed to warm up to room temperature and kept being stirred overnight[5]. The reaction was quenched with H_2O, the organic layer was separated and the water phase was extracted with ethyl acetate (3 × 50 mL). The combined organic layers were dried with Na_2SO_4 and evaporated under vacuum to yield a crude product (a white solid) which was purified by column chromatography on silica gel to give **7** as a white solid (yield 50%) ^{1}H NMR (400 MHz, $CDCl_3$) δ (ppm): 1.14 (d, 9H, J = 16.77 Hz); 6.46-7.60 (d, 1H, J = 453.96 Hz); 7.47-7.5 (m, 2H); 7.55-7.57 (d, 1H); 7.65-7.70 (m, 2H). ^{13}C NMR (101

MHz, $CDCl_3$) δ) δ (ppm): 132.44 (s), 130.94-130.84 (d, ^{1}J = 10.27 Hz), 128.54-128.42 (d, ^{2}J = 12.04 Hz), 32.31-31.62 (d, ^{3}J = 70.27Hz), 23.44-23.42 (s). ^{31}P NMR (162 MHz, $CDCl_3$) δ (ppm): 47.46.

Synthesis of benzyl 2-(tert-butylhydrophosphoryl)-2-methylpropanoate (8)

A solution of iodine in dry tetrahydrofuran (10 mL) was added in to a round bottom bottle charged with a suspension of Zinc powder (8 mmol) in tetrahydrofuran. A solution of (1-(benzyloxy)-2-methyl-1-oxopropan-2-yl)zinc was then prepared by slowly adding benzyl 2-bromo-2-methylpropanoate (7.5 mmol) to the stirred discolored suspension. 4 h later, t-butyldichlorophosphine (8 mmol) in dry tetrahydrofuran (10 mL) was added into the reaction mixture dropwise, and the reaction was kept stirred at RT for another 10 h[6]. The solution was acidified with 10% HCl (20 mL). The organic layer was separated and aqueous layer was extracted with chloroform (3 × 50 mL). The organic layers were combined and dried over magnesium sulfate, filtered, and evaporated under vacuum to yield a crude product which was purified by column chromatography on silica gel using dichloromethane and methanol (100:1) as eluent to give **8** as a light yellow solid (yield 50%). ^{1}H NMR (600 MHz, $CDCl_3$) δ (ppm):1.14 (d, 9H, J = 16.35 Hz); 1.55 (s, 3H); 1.62 (d, 6H, J_1 = 12.38 Hz, J_2 = 14.47 Hz); 5.14 (d, 2H, J_1 = 12.28Hz, J_2 = 12.28 Hz); 6.51 (d, 1H, J = 464.93 Hz); 7.35 (M, 5H, J_1= 7.31 Hz, J_2 = 8.76 Hz). ^{13}C NMR (151 MHz, $CDCl_3$) δ (ppm): 173.02 (s), 134.94 (s), 128.69-128.31 (d, ^{1}J = 58.46 Hz), 67.32 (s), 44.11-43.78 (d, ^{2}J = 50.62 Hz), 34.23-33.83 (d, ^{3}J = 67.81 Hz), 24.79-24.78 (d, ^{4}J = 1.23 Hz).^{31}P NMR (243 MHz, $CDCl_3$) δ(ppm): 58.60 (d, 1P, J = 464.53 Hz).

Synthesis of 2-(tert-butylhydrophosphoryl)-2-methylpropanoic acid (9)

A solution of charcoal (90wt%/Pd) in 5 mL anhydrous methanol were added to a stirred solution of **8** (5 mmol in methanol, 6.5 mL) at room temperature. H_2 was bubbled into the stirred reaction mixture continuously. 12 h later, the mixture was filtered and concentrated under reduced pressure[7]. The resulting solid was subjected

to a short column chromatography on silica gel using dichloromethane and methanol (20:1) as eluent to give the corresponding white solid product **9** (yield 80%). ^{1}H NMR (600 MHz, DMSO-d_6) δ (ppm):1.13 (d, 9H, J = 15.73 Hz); 1.37 (d, 6H, J_1 = 14.38 Hz, J_2 = 14.38 Hz); 6.37 (d, 1H, J = 459.88 Hz); 13.07 (s, 1H). ^{13}C NMR (151 MHz, DMSO) δ (ppm): 174.89 (s), 43.85-43.53 (d, ^{1}J = 52.01 Hz), 34.31-33.91 (d, ^{2}J= 67.37 Hz), 24.97-24.96 (s), 23.84-23.84 (s). ^{31}P NMR (243 MHz, DMSO-d_6) δ (ppm): 56.94 (d, 1P, J = 459.03 Hz).

Synthesis of 2,3,5,6-tetrafluorophenyl 2-(*tert*-butylhydrophosphoryl)-2-methylpropanoate (10)

Dicyclohexylcarbodiimide (DCC, 268.0 mg, 1.3 mmol) dissolved in 1 mL N,N-dimethylformamide (DMF) was added dropwise into a solution of **9** (192 mg, 1.0 mmol), 4-dimethylaminopyridine (DMAP, 12.2 mg, 0.1 mmol) and 2,3,5,6-tetrafluorophenol (163 mg, 1 mmol) in 3 mL THF at room temperature under the protection of nitrogen. Then the mixture was stirred 12 h at ambient temperature, following which, the mixture was filtered and the filtrate was concentrated under reduced pressure[8]. The resulting residue was purified via preparation silica gel plate with dichloromethane and methanol (40:1) to give product **10** as a white solid (yield 75%). ^{1}H NMR (600 MHz, $CDCl_3$) δ (ppm):1.30 (d, 9H, J = 16.75 Hz); 1.75 (dd, 6H, J_1 = 13.62 Hz, J_2 = 11.75 Hz); 6.37 (d, 1H, J = 464.41 Hz); 7.06 (m, 1H). ^{13}C NMR (151 MHz, $CDCl_3$) δ (ppm): 133.15 (s, CH); 132.34 (d, J = 10.29 Hz, CH, 2C); 128.45(d, J = 12.65, CH, 2C); 125.73 (m, ^{1}J = 19.06 Hz, ^{2}J = 18.88 Hz, Cq); 32.84 (m, ^{1}J = 15.36 Hz, ^{2}J = 15.36 Hz, Cq); 23.71 (s, CH_3, 3C). ^{31}P NMR (243 MHz, $CDCl_3$) δ (ppm): 54.99-56.96 (d, 1P, J = 479.89 Hz).

Synthesis of DBPOF-c(RGDyk)

c(RGDyk) (1.0 mg) and **5** (3.0 mg) were dissolved in 100 μL anhydrous DMF. Then 5 μL triethylamine was added into the solution and reacted 12 h. The crude product was purified by HPLC. MS calcd for $C_{35}H_{55}FN_9O_{10}P^+$: 811.37, found, 812.52 $[M + H]^+$.

Synthesis of DBPOF-HSA

A solution of **5** (0.5 mg) in DMSO (50 μL) was added in a solution of human serum albumin (53 mg, Brussels, Belgium) in 0.01 M sodium bicarbonate (1.5 mL, pH 7.36). The mixture was incubated for 2 h at room temperature. The conjugate was purified by HiTrapTM desalting column (GE, USA) with sodium bicarbonate (0.01 M, pH 7.36). The purified product was analyzed by a Xtimate SEC-300 column (Welch, China). UV detection of the eluate was performed at 280 nm.

Radiosynthesis of ^{18}F-labeled organofluorophosphine

Approximately 1.0 mg of the precursor (*e.g.* **2**, **3** or **4**) was dissolved in 20 μL DMSO in a clean glass vial. No carrier-added [^{18}F]F$^-$ (2-10 mCi, approximately 180 μL, [^{18}F]F$^-$ was dissolved in aqueous solution) from bombardment of $H_2{}^{18}O$ with 18 MeV protons was diluted, distributed and added into the reaction vial. After incubating at room temperature for 5-15 min, the reaction was quenched by adding 10 mL of water. The mixture was passed through a Sep-Pak C18 light cartridge (Waters, USA) and washed with 10 mL of water twice to remove free [^{18}F]F$^-$. The radiolabeled product [^{18}F]**2** (or [^{18}F]**3**, [^{18}F]**4**) was eluted off a Sep-Pak C18 light cartridge (Waters, USA) using 1.0 mL of acetonitrile. RCYs of the purified [^{18}F]**2** (or [^{18}F]**3,** [^{18}F]**4**) were analyzed by a radio-HPLC. The radiolabeling was repeated at least three times for each precursor under respective conditions.

^{8}F-labeling condition optimization

Organofluorophosphine fluoride acceptors, diphenylphosphinic fluoride (**2**), tert-butyl(phenyl)phosphinic fluoride (**3**) and benzyl 2-(tert-butylfluorophosphoryl)-2-methylpropanoate (**4**) were used as substrates to explore the conditions and RCYs of radiofluorination.

Method I: 3.0 μmol precursor was dissolved in 10 μL DMSO. 20 μL aqueous [^{18}F]F$^-$ solution was added to reaction and the total reaction volume was adjusted to 200 μL

with 170 μL pure water. The reaction was gently shaken at room temperature for 5-15 min obtain the desired product.

Method II: 3.0 μmol precursor was dissolved in 10 μL DMSO. 20 μL aqueous $[^{18}F]F^-$ solution was added to reaction and the total reaction volume was adjusted to 200 μL with 170 μL pure water. The reaction was gently shaken at 75 °C for 5-15 min to obtain the desired product.

Method III: 3.0 μmol precursor was dissolved in 100 μL DMSO. 20 μL aqueous $[^{18}F]F^-$ solution was added to reaction and adjust the total reaction volume to 200 μL with 80 μL pure water. The reaction was gently shaken at room temperature for 5-15 min to obtain the desired product.

Method IV: 3.0 μmol precursor was dissolved in 100 μL DMSO. 20 μL aqueous $[^{18}F]F^-$ solution was added to reaction and adjust the total reaction volume to 200 μL with 80 μL pure water. The reaction was gently shaken at 75 °C for 5-15 min to obtain the desired product.

Method V: 3.0 μmol precursor was dissolved in 200 μL DMSO and was added to reaction bottle with dried $[^{18}F]F^-$ anion. The reaction was gently shaken at room temperature for 5-15 min to obtain the desired product.

Method VI: 3.0 μmol precursor was dissolved in 200 μL DMSO and was added to reaction bottle with dried $[^{18}F]F^-$ anion. The reaction was gently shaken at 75 °C for 5-15 min to obtain the desired product.

***In vitro* stabilities of ^{18}F-labeled organofluorophosphine**

Purified $[^{18}F]$**2** (or $[^{18}F]$**3,** or $[^{18}F]$**4**) (~1 mCi in 20 μL ethanol) was added into 180 μL saline or ethanol. The mixture was then incubated at 37 °C. After 2 h incubation, the RCP was assayed by a radio-HPLC. The stabilities in serum were determined in the same way with 180 μL mouse serum for each test. The mixture was precipitated by addition of 200 μL acetonitrile and centrifuged at 6,893 xg for 5 min at room temperature. Filtered by a 0.22 μm Millipore filter, the RCP of the filtrate was then analyzed by a radio-HPLC.

Metabolic stabilities of ^{18}F-labeled organofluorophosphines in normal mice

The metabolic stability study was performed according to a published procedure[9]. [^{18}F]**3** (or [^{18}F]**4**) (2 mCi in 100 μL saline) was i.v. injected into each normal mouse (n = 3). The animals were sacrificed and dissected at 120 min after injection. Blood (500 μL) was collected and acetonitrile (500 μL) was added to precipitate insoluble proteins from the solution. The insoluble material was removed by centrifuging at 6,893 xg for 5 min. The supernatant was passed through a 0.22 μm Millipore filter and 20 μL of the supernatant was analyzed by a radio-HPLC.

MicroPET/CT imaging with [^{18}F]4

Normal ICR mice were employed in microPET/CT imaging (n = 3) and anesthetization was conducted with 2.5% isoflurane during preparation and 1.5% during scans. 50 min (30-80 min post i.v. injection) dynamic whole-body microPET scans were acquired by an Inveon microPET/CT (Siemens, Germany) 30 min after intravenous injection of [^{18}F]**4** (100 μL, 100 μCi). Reconstruction of PET images was performed with three-dimensional ordered subset expectation maximization (3D OPMAP) algorithm.

Automated radiosynthesis of ^{18}F-DBPOF-c(RGDyk)

No-carrier-added [^{18}F]F$^-$ was produced via the [^{18}O (p, n) ^{18}F] nuclear reaction by irradiation of [^{18}O]H_2O on a RDS 111 cyclotron (Siemens, Germany). The automated synthesis of ^{18}F-DBPOF-c(RGDyk) was performed on a commercial multifunction radiosynthesis module PET-MF-2V-IT-I (Beijing PET Technology, China). A schematic layout of the automated synthesis module to produce ^{18}F-DBPOF-c(RGDyk) is shown in Supplementary Figure 4. ^{18}F-DBPOF-c(RGDyk) was prepared following the stepwise procedure:

1. The precursor [0.8-2.4 mg dissolved in 0.5 mL solvent, DMSO:H_2O = 1:1 (v/v), vessel 3] was introduced to the reaction vessel. [^{18}F]F$^-$ (600-1200 mCi) in target water

was added into the reaction vessel which was then kept temperature at 25 °C for fluorination and held at this temperature for 15 min and without stirring.

2. The reaction mixture was diluted with 10 mL of water (vessel 4), before being transferred to pass through a C18 cartridge (Waters, 25 μL). The cartridge was washed out with water (10 mL, vessel 5).

3. The C18 cartridge was then eluted with USP ethanol (1.0 mL, vessel 11) into the product collection vessel pre-charged with 0.9% sodium chloride for injection (9.0 mL), resulting in less than 10% ethanol content overall.

4. The final product was filtered with a 0.22-μm sterile Millex® GV (vented filter, Millipore, Billerica, MA, USA) into a sterile dose vial (Mallinckrodt, Hazelwood, MO, USA) fitted with a vent needle (Sartorius Stedim Biotech GmbH, Göttingen, Germany).

Visual inspection of ^{18}F-DBPOF-c(RGDyk) injection

Using remote handling equipment and appropriate radiation shielding (leaded glass), the vial containing the ^{18}F-DBPOF-c(RGDyk) product was visually inspected under bright light. The product was clear and colorless with no evidence of foreign matter.

Radiochemical identity

Radiochemical identity of ^{18}F-DBPOF-c(RGDyk)

The radiochemical identity of ^{18}F-DBPOF-c(RGDyk) was determined by HPLC using an Agilent 1260 Infinity System incorporating a quaternary pump, HiPALS autosampler, and DAD ultraviolet (UV) detector with a Max-Light flow cell set to 254 nm plus a Bioscan Flow-Count interface with a NaI radioactivity detector. Chromatographic data were acquired and analyzed on an Agilent OpenLAB chromatography data system (Rev.A.04.02). The following chromatographic conditions were used: Kromasil-C18 4.6 × 250 nm analytical column, eluent A: H_2O, eluent B: acetonitrile, gradient started at 90% (v/v) A, ramping up to 80% B over 25 min then held constant throughout the run, flow rate: 1.0 mL/min, UV = 280 nm. The

retention time of the reference material DBPOF-c(RGDyk), as determined by UV detector was consistent with the retention time of the ^{18}F-DBPOF-c(RGDyk), as determined by the radiation detector, with appropriate correction for the offset between the two detector systems.

RCP of of ^{18}F-DBPOF-c(RGDyk) injection

Using the same HPLC system described for the radiochemical identity test, an appropriate volume of ^{18}F-DBPOF-c(RGDyk) was injected at a quantity injected that avoids uncorrected dead-time loss (for main peak) in the radioactive detection system. The RCY of ^{18}F-DBPOF-c(RGDyk) was determined by dividing the radioactivity associated with the ^{18}F-DBPOF-c(RGDyk) peak by total activity assayed in the chromatogram multiplied by 100. The product met this acceptance specification and the radiochemical purity was greater than 95%.

Molar activity of ^{18}F-DBPOF-c(RGDyk)

The molar activity of DBPOF-c(RGDyk) was calculated by dividing the assayed radioactivity of a calibrated aliquot of ^{18}F-DBPOF-c(RGDyk) (mCi/mL at end of synthesis) by the molar amount of carrier DBPOF-c(RGDyk) measured by HPLC-UV [nmol of DBPOF-c(RGDyk)] as interpreted from the standard mass calibration curve.

Chemical purity of ^{18}F-DBPOF-c(RGDyk) injection

Using the same HPLC system described for the radiochemical identity test, the carrier mass of ^{18}F-DBPOF-c(RGDyk) was determined. After the initial HPLC column void volume, all other UV peaks were summed and attributed to by-products and less than 5%. ^{18}F-DBPOF-c(RGDyk) was successfully synthesized and radiolabeled with >95% purity.

Residual solvent analysis of ^{18}F-DBPOF-c(RGDyk) injection

Analyses of residual solvent levels in ^{18}F-DBPOF-c(RGDyk) were conducted using an Agilent 5979I gas chromatograph. The product met this acceptance specification and the DMSO level was less than 10 ppm.

pH of ^{18}F-DBPOF-c(RGDyk) injection

A drop of the ^{18}F-DBPOF-c(RGDyk) final product matrix was applied to pH indicator paper (Newstar pH-Indicator strip, pH 5.5-9.0.). The strip color was matched to an indicator chart. The product met this acceptance specification and the pH was between 7.0 and 7.5.

The standard UV curve of DBPOF-c(RGDyk)

Solutions of DBPOF-c(RGDyk) at graded concentrations (0.6, 0.9, 1.2, 1.5, 2.0, 3.0 nmol/0.1 mL) were prepared and analyzed by an analytical HPLC (isocratic elution, H_2O:methanol = 35:65, flow rate: 1.0 mL/min, UV = 280 nm). The UV absorption peak areas at different concentrations of DBPOF-c(RGDyk) were measured and the relationship between the areas of the absorption peaks and the concentrations of the substance was obtained by linear analysis.

Calculation of molar activities of ^{18}F-DBPOF-c(RGDyk)

A linear standard curve was generated by injecting various quantities of compound DBPOF-c(RGDyk) determined by weight. The specific activity of DBPOF-c(RGDyk) was calculated by dividing the assayed radioactivity of a calibrated aliquot of $[^{18}F]F^{-}$DBPOF-c(RGDyk) (mCi/mL at end of synthesis) by the molar concentration of carrier DBPOF-c(RGDyk) measured by HPLC-UV (nmol of DBPOF-c(RGDyk) per mL) as interpreted from the standard mass calibration curve. The molar amount of ^{18}F-DBPOF-c(RGDyk) was directly measured by correlating UV-absorbance to a standard curve in Supplementary Figure 8.

For example, for the automated radiosynthesis in Supplementary Figure 5, integration of the visible peak (280 nm) that eluted at 11.4 min provided a quantitative molar

value of 3-5 nmol based on the fitted linear standard curve. Meanwhile, 300-400 μCi radioactivity was collected from HPLC. Hence, the molar activity was measured to be activity at the time of collection/molar amount.

Molar activity of ^{18}F-DBPOF-c(RGDyk) = activity of ^{18}F-DBPOF-c(RGDyk)/ molar amount of DBPOF-c(RGDyk) = 300-400 μCi/3-5 nmol = 0.06-0.13 Ci/μmol.

For manual radiosynthesis,

Molar activity of ^{18}F-DBPOF-c(RGDyk) = activity of ^{18}F-DBPOF-c(RGDyk)/molar amount of DBPOF-c(RGDyk) = 37-50 μCi/5-6 nmol = 0.006-0.01 Ci/μmol.

In vitro stability of ^{18}F-DBPOF-c(RGDyk) in rat plasma

A volume of 100 μL of purified ^{18}F-DBPOF-c(RGDyk) was added to 1.0 mL freshly isolated ethanol, serum, or saline respectively. The mixtures were incubated at 37 °C for 2 h and then analyzed by a radio-HPLC equipped with a Kromasil-C18 column.

In vitro stability of ^{18}F-DBPOF-HAS in rat plasma

A volume of 100 μL of purified ^{18}F-DBPOF-HSA (22 MBq/0.6 mCi) was added to 900 μL serum. The mixtures were incubated at 37 °C and analyzed at 1, 2 and 3 h time points by a radio-HPLC equipped with Xtimate SEC-300 column (Welch, China).

Glioblastoma tumor mouse model

All animal studies were performed under the animal use and care regulations approved by Center of Animal Care and Use Committee, Xiamen University. BALB/c nude mice (18-22 g) were purchased from Beijing Vital River Laboratory Animal Technology (China).

The origins of U87MG cell lines used in the Methods section were from ATCC (cell line name U-87MG ATCC). Human glioblastoma U87MG cell line was cultured at

37 °C in a humidified atmosphere containing 5% CO_2 in Dulbecco's Modified Eagle Medium (DMEM, high glucose) supplemented with 10% fetal bovine serum. Each male BALB/c nude mouse was injected subcutaneously in the right foreleg with about 2×10^6 U87MG cells. At 4-5 week after inoculation, when the tumors reached about 0.5 cm in diameter, the mice were used for PET/CT imaging studies.

MicroPET/CT imaging with ^{18}F-DBPOF-c(RGDyk)

Glioblastoma tumor mice were employed in microPET/CT imaging (n = 3) with ^{18}F-DBPOF-c(RGDyk). Anesthetization was conducted with 2.5% isoflurane during preparation and 1.5% during scans. 10 min dynamic microPET-CT scans were acquired by an Inveon microPET/CT (Siemens, Germany) 20 min post i.v. injection of ^{18}F-DBPOF-c(RGDyk) (100 µL, 100 µCi). Reconstruction of PET images was performed with 3D OPMAP algorithm.

In order to study the specific tumor uptake, mice were pre-blocked by injection of 10 µmol DBPOF-c(RGDyk) 30 min prior to injection of ^{18}F-DBPOF-c(RGDyk) as the control group. Then 60 min dynamic images were acquired to provide a control group (n = 3). This study showed tumor uptake（SUV）in the unblocked mice of 1.04~2.41 while the average tumor uptake（SUV） in the blocked mice was only 0.32~1.12. The tumor uptake of unblocked mice is 1.39~2.51 times higher than blocked mice. The tumor was clearly observable whereas blocked mice had much less tumor uptake as indicated in Supplementary Figure 13.

MicroPET/CT imaging with ^{18}F-DBPOF-HSA

Healthy female Wistar rats were employed in microPET/CT imaging (n = 3) with ^{18}F-DBPOF-HSA. Anesthetization was conducted with 2.5% isoflurane during preparation and 1.5% during scans. 60 min dynamic microPET scans were acquired by an Inveon microPET/CT (Siemens, Germany) after intravenous injection of ^{18}F-DBPOF-HSA (200 µL, 200 µCi). Reconstruction of PET images was performed with 3D OPMAP algorithm.

Log *D* of ^{18}F-DBPOF-c(RGDyk)

Log *D* of ^{18}F-DBPOF-c(RGDyk) was measured in a pre-saturated 1-octanol/PBS (pH = 7.4) system to estimate the hydrophobicity changes between c(RGDyk) and ^{18}F-DBPOF-c(RGDyk) that may influence biological function of c(RGDyk). 1-Octanol (2.0 mL) and phosphate buffer (2.0 mL) were pipetted into four 12 mL test tubes containing 5-10 mL of radiotracer. The test tubes were stoppered, mechanically shaken for 10 min, and centrifuged (5 min). From each tube, approximately 0.5 mL of buffer was transferred into a pre-weighed test tube for counting. The remaining buffer was discarded and approximately 0.5 mL of octanol phase was also transferred into a separate pre-weighed test tube. The amount of radioactivity in each tube was measured by a γ counter and corrected for decay. Accurate volumes of each counted phase were determined by weight differences and known densities. The partition coefficient was calculated using Supplementary Equation 1:

$$\text{Log } D = \frac{\text{Counts/mL in octanol}}{\text{Counts/mL in buffer}} = -0.59 \pm 0.09 \qquad (1)$$

Log *D* of -0.59 ± 0.09 for ^{18}F-DBPOF-c(RGDyk) falls in an ideal range to ensure high affinity for the integrin receptor[10].

Cell cytotoxic analysis of DBPOF-c(RGDyk)

Photosensitizers were added at concentrations 0, 0.01, 0.05, 0.1, 0.5, 1 and 5 mM. The cell cytotoxic effect on the U87MG cells were determined using the CCK8 assay. After 12 h and 24 h of incubation after the treatment we added 10 μL of CCK8 and incubated the cells for another 1 h at 37 °C and in 5% CO_2. The measurement of absorbance was carried out on a 96-well microplate reader Synergy HT at 450 nm. The cell viability of the samples was determined as a percentage of the control cell viability (100 × average of test group/average of control group). Using the Phototox version 2.0 software data were calculated for the determination of cell activity[11].

Theoretical calculation

1. Mechanism of H_2O-resistant F/F exchange interpreted by free-energy parameters in DFT experiments.

The mechanism for quick substitution of the covalently bonded F on the organophosphine prosthesis by F^- ($F^-\cdot H_2O$) in the water phase was rationally investigated with density functional theory. Two reaction pathways with either F or O as the attacking atom were examined respectively (shown in Figure 6 and Supplementary Figure 16).

The concerted mechanisms may occur via TS1 and TS2 two steps. To simply mimic the solvation effect of water, the one-water-containing anionic species $[H_2O\cdot F]^-$ was modeled as the initial nucleophile. The first step of F-F exchange was that $[H_2O\cdot F]^-$ attacked P via F to reach the transition state TS1-F-H_2O. Ten coordination intermediates INT-F-H_2O were therefore formed. In the second step, the other side F acted as a leaving group. Over the transition state TS2-F-H_2O, F-F exchange products generated.

OH-F exchange also followed a two-step mechanism. The O from H_2O attacked P to form a P-OH bond while the H_2O took off a proton. The first step was nucleophilic attack of O, undergoing transition state TS1-OH-H_2O to give intermediate pentacoordinate INT-OH-H_2O. In the second step, F on the other side acted as a leaving group to form OH-F exchange products.

2. Charge distribution of organofluorophosphine fluoride acceptors (**1** - **5**)[12] was showed in Supplementary Figure 18.

Supplementary References

1 Becke, A. D. Density‐functional thermochemistry. III. The role of exact exchange. *J Chem Phys.* **98**, 5648-5652 (1993).

2 Yanai, T., Tew, D. P. & Handy, N. C. A new hybrid exchange–correlation functional using the Coulomb-attenuating method (CAM-B3LYP). *Chem Phys Lett.* **393**, 51-57 (2004).

3 Purohit, A. K. *et al.* A single-step one pot synthesis of dialkyl fluorophosphates from dialkylphosphites. *Tetrahedron Lett.* **56**, 4593-4595 (2015).

4 Molitor, S., Becker, J. & Gessner, V. H. Selective dehydrocoupling of phosphines by lithium chloride carbenoids. *J Am Chem Soc.* **136**, 15517-15520 (2014).

5 Schweizer, S., Becht, J.-M. & Le Drian, C. Highly efficient reusable polymer-supported Pd catalysts of general use for the Suzuki reaction. *Tetrahedron.* **66**, 765-772 (2010).

6 Kielbasinski, P. & Mikolajczyk M. Novel-approach to the synthesis of alkoxycarbonylmethylphosphine and bis(alkoxycarbonylmethyl)phosphine pxides based on a Reformatsky-type reaction. *Synthesis-Stuttgart* **2**,144-146 (1995).

7 Felpin, F. X. & Fouquet, E. A useful, reliable and safer protocol for hydrogenation and the hydrogenolysis of O-benzyl groups: the in situ preparation of an active Pd(0)/C catalyst with well-defined properties. *Chemistry.* **16**, 12440-12445 (2010).

8 Kolodych, S. *et al.* CBTF: new amine-to-thiol coupling reagent for preparation of antibody conjugates with increased plasma stability. *Bioconjug Chem.* **26**, 197-200 (2015).

9 Mou, T. *et al.* Synthesis and preliminary evaluation of 18F-labeled pyridaben analogues for myocardial perfusion imaging with PET. *J nucl med.* **53**, 472-479 (2012).

10 Alan A. Wilson. *et al.* An admonition when measuring the lipophilicity of radiotracers using counting. *Appl Radiat Isot.* **54**, 203-208(2001).

11 Malina, L. *et al.* The in vitro cytotoxicity of metal-complexes of porphyrin sensitizer intended for photodynamic therapy. *Toxicol In Vitro.* **34**, 246-256 (2016).

12 Lu, T. & Chen, F. Multiwfn: a multifunctional wavefunction analyzer. *J Comput Chem.* **33**, 580-592 (2012).
